# A comprehensive single cell transcriptional landscape of human hematopoietic progenitors

**DOI:** 10.1038/s41467-019-10291-0

**Published:** 2019-06-03

**Authors:** Danilo Pellin, Mariana Loperfido, Cristina Baricordi, Samuel L. Wolock, Annita Montepeloso, Olga K. Weinberg, Alessandra Biffi, Allon M. Klein, Luca Biasco

**Affiliations:** 1 Gene Therapy Program, Dana-Farber/Boston Children’s Cancer and Blood Disorders Center, Harvard Medical School, Boston, MA 02115 USA; 2 Department of Systems Biology, Harvard Medical School, Boston, MA 02115 USA; 3 Department of Pathology, Boston Children’s Hospital and Harvard Medical School, Boston, MA 02115 USA; 4 University College of London (UCL), Great Ormond Street Institute of Child Health Faculty of Population Health Sciences, London, WC1N 1EH UK

**Keywords:** Computational biology and bioinformatics, Haematopoietic stem cells, Stem-cell differentiation

## Abstract

Hematopoietic Stem/Progenitor cells (HSPCs) are endowed with the role of maintaining a diverse pool of blood cells throughout the human life. Despite recent efforts, the nature of the early cell fate decisions remains contentious. Using single-cell RNA-Seq, we show that existing approaches to stratify bone marrow CD34+ cells reveal a hierarchically-structured transcriptional landscape of hematopoietic differentiation. Still, this landscape misses important early fate decisions. We here provide a broader transcriptional profiling of bone marrow lineage negative hematopoietic progenitors that recovers a key missing branchpoint into basophils and expands our understanding of the underlying structure of early adult human haematopoiesis. We also show that this map has strong similarities in topology and gene expression to that found in mouse. Finally, we identify the sialomucin CD164, as a reliable marker for the earliest branches of HSPCs specification and we showed how its use can foster the design of alternative transplantation cell products.

## Abstract (web summary)

Human Hematopoietic stem and progenitor cells (HSPCs) are commonly defined by CD34 expression. Here, the authors map single-cell RNA states both inside and outside the CD34 compartment, uncovering previously unappreciated branchpoints and validating CD164 as an efficient marker for early HSPCs.

## Introduction

In humans, there have been conflicting proposals for the hierarchical relationships linking different hematopoietic progenitors^1–7^. In the conventional depiction of human haematopoiesis, supported by lineage-tracing studies in the mouse^8^, the earliest branching splits lymphoid vs myelo/erythroid fate commitment. Conversely, in a recent challenge of the classical view, it has been suggested that multipotent progenitors could undergo a very early fate decision towards the megakaryocyte lineage followed by a single step-wise transition to either erythroid, myeloid, or lymphoid commitment^9^. The advent of single-cell RNA sequencing (scRNA-Seq) has not only created an opportunity to improve our understanding of the nature of human haematopoiesis through the study of transcriptional single-cell states^10–12^, but also generated conflicting observations. Initial use of this technology in humans led to an alternative view that early haematopoiesis is composed by a continuum of low-primed undifferentiated haematopoietic stem and progenitor cells (CLOUD-HSPCs) from which unilineage-restricted cells emerge^10^. Recently, scRNA-Seq data combined with assays of chromatin accessibility supported instead the notion of a structured hierarchy, revealing a variegated hematopoietic landscape^13^, the existence of lineage-biased stem cells in mice^14,15^ and of different stages of human lymphoid commitment in humans^16,17^.

Human HSPCs are commonly identified by expression of the antigen CD34^18^. CD34+ cells are heterogeneous, and there are ongoing efforts to classify their substructure by immunophenotyping and according to their differentiation and in vivo survival potential^5^. The CD34+ cell population structure is unresolved, with recent studies showing that the current immunophenotypically defined CD34+ subsets could be more heterogeneous than previously thought^9,19^. A possible reason for the lack of resolution is that enrichment methods for CD34+ cells may bias the representation of cell states during early hematopoietic commitment, as the CD34 marker is downregulated at different rates along commitment to different cell fates^20,21^. In this regard, one should note that previous single-cell studies on human hematopoiesis focused exclusively on the whole CD34+ population (comprising both Lin− and Lin+ cells)^11^, or on in silico modeling of the fate commitment of the CD34+ fraction containing the least differentiated HSPCs^10^.

We here aim at providing insights on the population structure of early hematopoietic commitment, by profiling human HSPCs with high-throughput scRNA-Seq^22,23^. Differently from previous works, in the present study we not only isolate the immature cells expressing the CD34 antigen, but we also extend our analysis to the whole bone marrow (BM) fraction lacking the main markers of terminal differentiation (Lineage negative, Lin− cells). The resulting scRNA maps provide a comprehensive transcriptional snapshot of the early human hematopoietic cell fates, shedding light on the origin of the basophil branching and on a previously unappreciated surface marker for fractionating the HSPCs cell product.

## Results

### Generating a high-resolution scRNA map of CD34+ progenitors

**Fig. 1:**
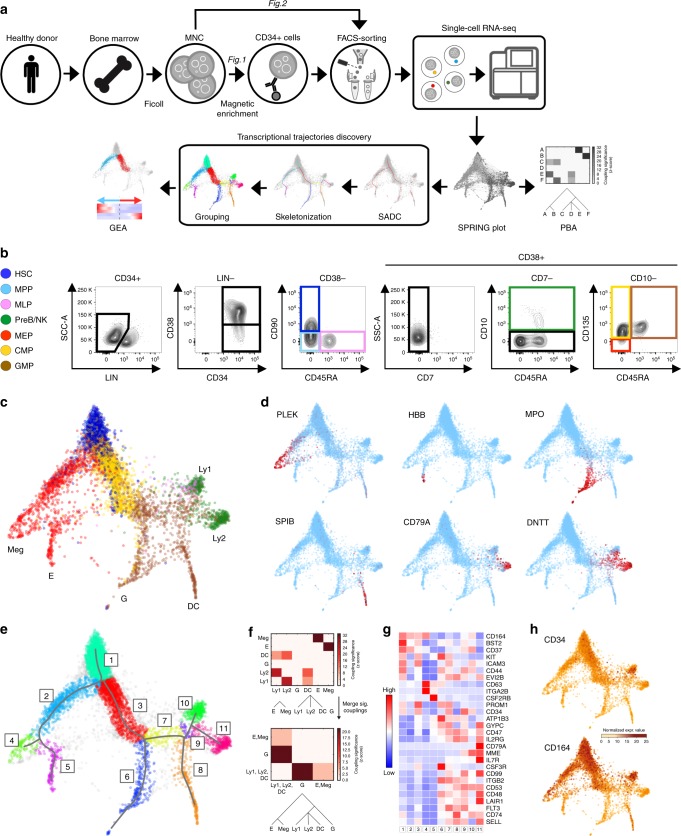
Experimental workflow and transcriptional map for human HSPCs. **a** Schematic for experimental design and workflow of data analysis. Two experiments have been performed on two separate healthy donors to generate two single-cell transcriptome maps (MNC, mononuclear cells; PBA, population balance analysis; GEA, Gene expression analysis). **b** Gating strategy used for the FACS sorting of seven HSPC subsets from magnetic beads purified CD34+ cells of a healthy donor BM (HSC, hematopoietic stem cells; MPP, multipotent progenitors; MLP, multi-lymphoid progenitors; Pre-B/NK, Pre-B lymphocytes/natural killer cells; MEP, megakaryocyte-erythroid progenitors; CMP, common myeloid progenitors; GMP, granulocyte–monocyte progenitors). **c** SPRING plot of the seven HSPCs single-cell transcriptomes. Each point is one cell. Labels at the edges represent the transcriptional states associated to early lineage commitment (Meg, megakaryocytes; E, erythroid cells; G, granulocytes; DC, dendritic cells; Ly1/Ly2, lymphoid B, T, NK cells). Color legend as in **b**. **d** Representative gene expression maps of lineage defining genes (PLEK, Meg; HBB, E; MPO, G; SPIB, DC; CD79A, and DNTT, Ly1/2). **e** Classification of individual cells into homogenous transcriptional groups numbered from 1 to 11, based on inferred principal trajectories (Supplementary Fig. 3a for details). **f** Predicted hierarchy based on two steps PBA. **g** Heatmap showing the expression average in groups shown in **e** for statistically significant genes coding for CD markers (likelihood ratio test [LRT] adjusted *p* value < 0.05). **h** Gene expression maps of CD34 and CD164

To establish a reference data set and to address the heterogeneity and fate potential of the known CD34+ subsets, our first investigations aimed at mapping at high-resolution the single-cell transcriptional states of cells commonly defined as human HSPCs (Fig. 1a). To this goal, we separated CD34+ cells purified by magnetic beads selection into seven subpopulations^5^, marking cells of differing fate potential (Fig. 1b) and tagged and sequenced the transcriptome of 6011 single cells (Supplementary Fig. 1a and Supplementary Table 1). We then used the scRNA-Seq data to infer the structure of cell states in high-dimensional gene expression space (Fig. 1c). We applied a visualization method previously developed for mouse hematopoietic progenitors^24^, whereby each cell represents a graph node, with graph edges linking nearest neighbor cells. The scRNA-Seq graph, visualized using SPRING force-directed layout^25^, shows a hierarchical, tree-like continuum of states, with branches that terminate at cells expressing recognizable transcriptional signatures of lineage commitment before the expression of final maturation markers (Fig. 1c, d) (megakaryocytes (Meg), erythroid cells (E), granulocytes (G), dendritic cells (DC), lymphoid cells (Ly1-2)). The structure of the single-cell data broadly partitions based on immunophenotypic subpopulations, but, significantly and in line with recent suggestions^9^, we observed that previously defined HSPC subpopulations hide substantial transcriptional heterogeneity (Supplementary Fig. 2a).

Our scRNA-Seq map of CD34+ subpopulations suggests that HSPCs do not undergo a single-step transition from CLOUD-HSPCs to unilineage states. Instead, they form a structured hierarchy (Fig. 1c). The earliest fate split separates erythroid–megakaryocyte progenitors from lymphoid–myeloid progenitors (LMPs), which separate further into lymphoid, DC and granulocytic progenitors. This hierarchy is highlighted by both inferred transcriptional trajectories (Fig. 1e and Supplementary Fig. 3a) and formal high-dimensional analysis of graph structure using the population balance analysis (PBA) algorithm^24^ (Fig. 1f)^24^. We conclude that human HSPCs are more organized than recently hypothesized and show more structure than appreciated by classical immunophenotyping.

### Extending the scRNA profiling to all BM progenitors

**Fig. 2:**
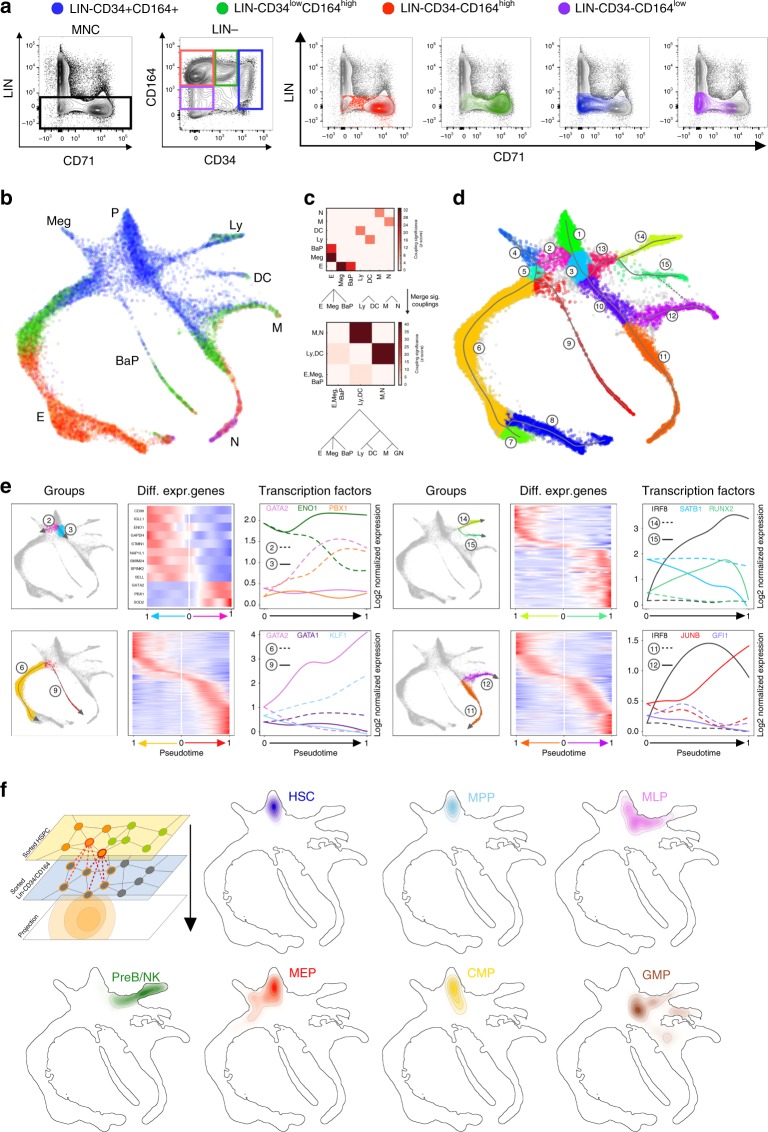
Human Lin− compartment investigation by means of CD34/CD164 fractionating. **a** Gating strategy for the FACS sorting of four subsets inside the Lin—fraction of a healthy donor BM, according to CD34 and CD164 expression (left panels). Relative contribution of CD71+ progenitors is shown in the right panels. **b** SPRING plot of the four Lin−CD34/CD164 subsets single-cell transcriptomes. Each point is one cell. Labels at the edges represent the transcriptional states associated to early lineage commitment (P, early progenitor cells; Meg, megakaryocytes; E, erythroid cells; BaP, basophil progenitors; N, neutrophils; M, monocytes; DC, dendritic cells; Ly-T/B/NK, lymphoid T/B/NK cells). Color legend as in **a**. Gene expression maps are available in Supplementary Fig. 4. **c** Predicted hierarchy based on two steps PBA. **d** Classification of individual cells into homogenous transcriptional groups numbered from 1 to 15, based on inferred principal trajectories. Solid lines show results based on final converged iteration (Supplementary Fig. 3b for details). Dashed lines added manually to highlight a potential additional trajectory not present in final iteration and inferred by visual inspection (DC-M). **e** Gene dynamics associated to branching and fate decisions. Plots on the left, branching and groups; mirror heatmaps, expression of statistically significant genes differentially expressed along each branch pseudotime (LRT adjusted *p* value < 0.05). Plots on the right, a selection of three transcription factors differentially expressed along each branch (LRT adjusted *p* value < 0.05). **f** Projection of the transcriptional states of the seven HSPCs onto the Lin−CD34/CD164 map

In the 1980 s, the wide adoption of monoclonal antibodies for immunophenotyping revealed that the CD34 antigen is an effective marker to isolate immature HSPCs from humans^18^. Since then, efforts have been made to define the hierarchical structure of HSPCs purified from immunomagnetic-selected CD34+ cells, under the assumption that this cell population effectively captures all early fate choices. Although our above analysis supports such efforts, we reasoned that a focus on CD34+ cells purified with magnetic beads enrichment might provide an incomplete view of the earliest branching events in haematopoiesis. We noted, for example, that branches towards basophils/eosinophils/mast cells and monocytes commitments were missing in our initial scRNA-Seq analysis of CD34 cells, despite these appearing as early events in mouse haematopoiesis^24^. In addition, many cells negative for mature lineage markers in human BM are CD34^low/−^ and could account for additional transitional states at which CD34 expression is rapidly downregulated, thus greatly reducing their probability of capture. Therefore, to generate a complete landscape of early haematopoiesis, we extended our analysis to encompass human CD34^low^ and CD34− cells. To this aim, we collected from a second healthy donor four fractions of BM Lin− cells, covering different degrees of maturation (Fig. 2a). The graded fluorescence-activated cell (FACS) sorting used in this analysis corrects for expansion of cells as they differentiate, allowing examination of early states alongside later ones that comprise the vast majority of Lin− progenitors. In fractionating the cells by maturity, we made use of a cell surface marker, CD164, that we identified from the initial data set as expressed by cells that are multipotent until just beyond the first E/Meg–LMP branchpoint (Fig. 1g, h). This fractionation strategy allowed us to preserve resolution of the single-cell events of the more-primitive compartments, whereas at the same time maintaining a full representation of the late cell fate branching (Fig. 2b; Supplementary Figs. 1b, 2b).

As predicted, the transcriptional map of the Lin− fraction, derived from the high-throughput clustering of 15,401 single cells (Fig. 2b; Supplementary Fig. 4 and Supplementary Table 1), revealed important early features that were missing from the analysis of the immune-selected CD34+ population. Using the same graph-based technique as for CD34+ cells, we could now identify later cell fate decisions. Monocyte progenitors seem to emerge from a common neutrophil/monocyte precursor later in the myeloid commitment and after the branching decision towards DC progenitors, with a possible contribution from DC progenitors as recently shown in the mouse^24,26^. These data also suggest that the identity of the remaining CD34− Lin− cells consists mostly of late neutrophil progenitors, and of a continuum of differentiating states towards erythroid commitment. Our results could be formalized, on a computational basis, by both high dimensions (using PBA algorithm, Fig. 2c) and inferred transcriptional trajectories (Fig. 2d and Supplementary Fig. 3b) and were confirmed upon analyzing the data with an independent method (Diffusion Maps^27^, Supplementary Fig. 5, 6) that does not rely on a limited amount of *k*-nearest neighbors (*k*NN) for data-embedding calculation. To generate a resource for further studies, we investigated the association between gene expression dynamics and cells progression along the estimated differentiation paths. We identified putative transcriptional switches occurring during early hematopoietic cell fate choices and genes exhibiting significant variations during lineage commitments (Fig. 2e; Supplementary Fig. 7 and Supplementary Information for the complete lists). This analysis contains valuable information for in vitro reprogramming efforts and for investigations into the origin of blood cell differentiation disorders and cancers.

To understand how the enrichment of CD34+ subsets could limit our view of early haematopoiesis, we projected the CD34+ HSPCs subpopulations onto the Lin− state map (Fig. 2f). The analysis confirmed that large portions of the Lin− map are strongly under-represented upon the magnetic pulldown of the CD34+ population (namely the ones identifying basophils, monocytes progenitors, and the stages of late erythroid differentiation). This supports the concept that the Lin− population structure provides a more complete view of key cell fate decisions along human hematopoietic commitment and suggests that, for a complete classification of HSPCs, analyses should be performed on FACS-sorted CD34+, CD34^low^ and CD34− compartments. Finally, with this projection we could appreciate the heterogeneous nature of the currently defined HSPC subsets, showing that they can be further fractionated into distinct and more homogenous transcriptional states (Supplementary Fig. 8).

### Exploring the origin of the human basophilic branch

**Fig. 3:**
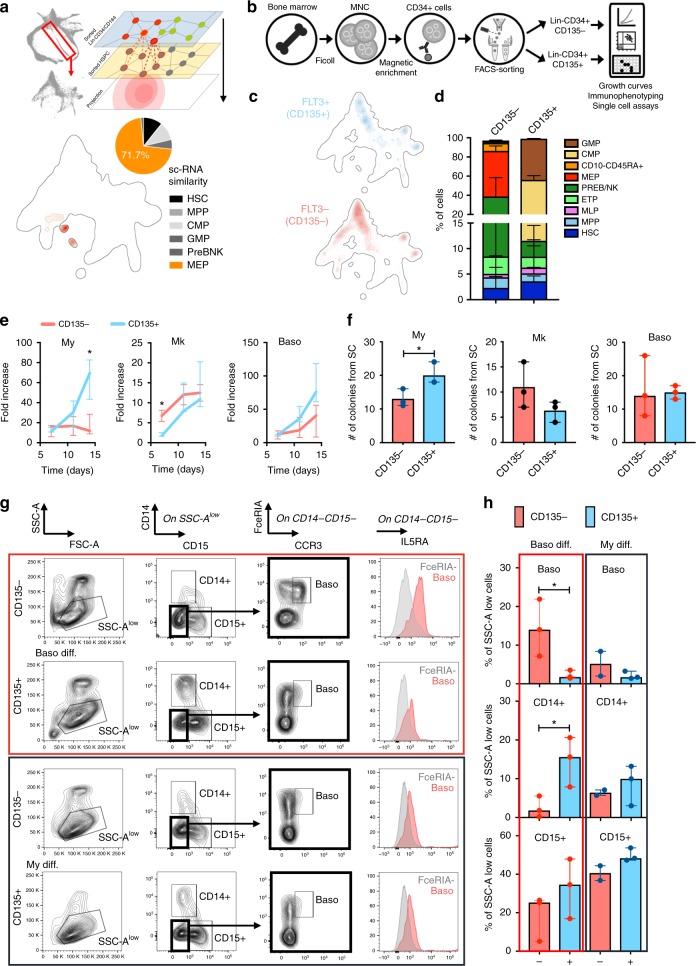
Cell fate analyses of Lin−CD34+CD135− cells support the MEP-associated origin of basophil progenitors. **a** Projection of the transcriptional profile of cells belonging to group 9 in Lin−CD34/CD164 data set onto sorted HSPCs map. Pie chart on the bottom represents the immunophenotipic characteristic for HSPC cells identified as most similar. **b** Experimental design. Lin−CD34+CD135− and Lin−CD34+CD135+ populations were sorted from the BM CD34+ cells of three healthy donors and their lineage potential was investigated through in vitro functional assays. **c** Spatial distribution estimated by using a two-dimensional kernel density estimator for cell exhibiting: top graph, high expression (at RNA level) of FLT3 gene (normalized expression > 0.9); bottom graph, no expression of FLT3 gene (normalized expression = 0). **d** Bar graph showing the content of HSPCs in CD135− and CD135+ fractions. Values are proportions estimates ± SE, estimated using method of moments and Dirichlet-Multinomial model. Hypothesis testing has been performed by means of independent samples, heteroscedastic, two-tailed Student’s *t* test. Details are provided in Supplementary Table 3. **e** Growth curves from three different culture conditions. My, Myeloid differentiating culture; Mk, Megakaryocyte differentiation culture; Baso, Basophil differentiation culture. Values are median ± error. Statistics by independent samples, two-tailed Student’s *t* test for each time point considered independently from the others (*p < 0.05). **f** Single-cell (SC) assay showing the total number of colonies obtained from CD135− and CD135+ fractions at the end of the three different culture conditions. Shown are median ± error. Statistics by independent samples, two-tailed Student’s *t* test (*p < 0.05). **g** FACS analysis of bona fide Basophils (Baso) defined as CD14−CD15−FceRIA+CCR3+IL5RA+ cells on CD135− and CD135+ populations upon basophil (upper panel) and myeloid (lower panel) differentiation culture. FceRIA− pick indicates the negative control. **h** Bar graphs summarizing the cytometric analysis described in **g**. Shown are the percentage of Baso, CD14+ cells and CD15+ cells on CD135− and CD135+ populations from the basophil (left panel) and myeloid (right panel) differentiation culture. Values are median ± error. Statistics by independent samples, two-tailed Student’s *t* test (*p < 0.05)

The most-notable result emerging from the exploration of our BM Lin− map was the identification of a branch toward cells carrying a transcriptional profile of early basophils specification. Strikingly, this class of basophils progenitors (BaP) was found to associate with erythroid and megakaryocyte fates and not with granulocytes precursors. Our data, generated on adult human BM, align with and expand on preliminary observations done in human cord blood CD34+ cells^11,28^, and in murine haematopoiesis^29^. To elaborate on this observation, we computationally projected the Basophil branch of our BM Lin− map onto the Lin− HSPC map to identify which, among the HSPC single cell states, had the highest scRNA similarity to this branch. The topological origin of the early basophil cell specification in the HSPC map was in striking accordance with what observed in the BM Lin− map and the highest level of similarity was detected with respect to the CD135− progenitors with known megakaryo–erythroid potential (MEP) (Fig. 3a). Building on these results, we thus designed and conducted a series of in vitro differentiation assays starting from FACS sorting Lin−CD34+ cells into CD135+ (FLT3+) (by definition containing common myeloid progenitors (CMP) and granulocyte–monocyte progenitors; GMP) and CD135−(FLT3−) (by definition containing MEP) cells (Fig. 3b–d and Supplementary Fig. 9c). These two groups of cells were separately put in culture in myeloid-, megakaryocytes (MK)-, and basophil-differentiating conditions under the hypothesis that if basophils are generated by CMP or GMP (as suggested by the classical model of haematopoiesis) the CD135+ fraction should be the one capable of differentiating into basophils after culture. As reported in Fig. 3, the Lin−CD34+4+CD135− and Lin−CD34+CD135+ populations had, as expected, specific growth preferences toward MK (the former) and myeloid (the latter) cell fates (Fig. 3e, f). The two populations grew at similar rate in basophilic conditions, but while the Lin−CD34+CD135+ fraction generated mostly CD14+ monocytes (Fig. 3g, h), the Lin−CD34+CD135− fraction emerged as the only population capable of giving rise with high efficiency to bona fide basophils (Fig. 3g, h; Supplementary Fig. 9b, d, e) defined as SSC-A^low^CD14-CD15-FceRIA+ CCR3+ IL5RA+ cells (as in Mori et al. 2009^30^ and in our immunophenotyping on human peripheral blood reported in Supplementary Fig. 9a). This observation is in line with our scRNA data showing that the basophil branch emerges from CD135− cells already committed toward a mixed MK/Erythroid/Basophil potential. Notably, because our experimental design purposely included also CD38− multipotent progenitors, one could have expected that basophils would have been generated at similar rates by the CD38− HSC/MPP that were present in both CD135+ and CD135− cell fractions (Fig. 3d). Conversely, the observation that only the CD135− cells were endowed with substantial basophilic potential strongly support the notion that the Lin−CD34+CD38−CD135− population might be already enriched in stem cells with very early priming towards a basophilic cell fate.

### Comparing human vs mouse hematopoietic scRNA-seq profiles

**Fig. 4:**
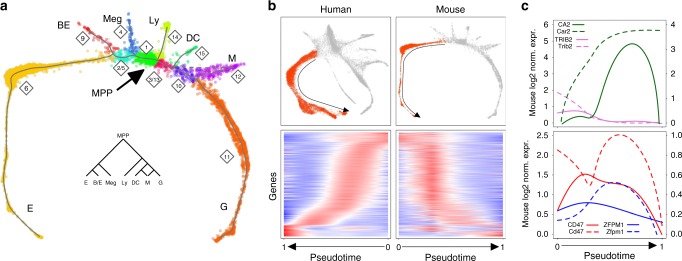
Human Lin−CD34/CD164 versus mouse Kit+ transcriptome map and gene expression dynamics analysis. **a** Classification of individual cells into 11 homogenous transcriptional groups, based on inferred principal trajectories on mouse Kit+ transcriptome data (Supplementary Fig. 3c for details). Group labels and colors have been set to highlight similarities with Lin−CD34/CD164 fractionating map. Solid lines show results based on final converged iteration (Supplementary Fig. 3c for details). Dashed lines added manually to highlight a potential additional trajectory not present in final iteration and suggested by PBA analysis reported in the middle (DC-M). MPP, MultiPotent progenitor cells; Meg, megakaryocytes; BE, baso/eosinophils; E, erythroid cells; Ly, lymphoid cells; DC, dendritic cells; M, monocytes; G, granulocytes. **b** Comparison of human and mouse transcriptional states during erythropoiesis. Upper panels, schemes of the comparison. Mirror heatmaps, expression of the 721 orthologous genes selectively expressed along the human and mouse erythroid differentiation (LRT adjusted *p* value < 0.05). **c** Representative comparable dynamics of the orthologues TRIB2/Trib2 and CA2/Car2 reported in Tusi et al.^24^ vs divergent dynamics of the orthologues CD47/Cd47 and ZFPM1/Zfpm1 reported in Pishesha et al.^31^

Another question of practical interest for modeling human disease is the relationship between human and mouse haematopoiesis. Although cell surface markers used to isolate HSPC subpopulations are known to differ between the two species, scRNA-Seq provides an opportunity to link population structure using whole-transcriptome information. We compared the scRNA-Seq map of the human Lin− population to that of mouse HSPCs, using data that we recently published on Kit+ mouse BM progenitors^24^. This analysis unveiled a strong similarity among the branching structures of haematopoiesis in the two organisms, with almost a 1:1 correspondence between hierarchies of cell states (Fig. 4a vs Fig. 2d, Supplementary Figs. 3c, 10). Furthermore, by comparing branch-specific gene signatures, we could identify that the vast majority of gene orthologous in the erythroid branch were equivalently expressed in human and mouse (Fig. 4b). Recently, we showed that erythroid progenitors in the mouse can be classified as early, which uniquely give rise to burst-forming units (BFU-E) and are marked by *Trib2*; and as committed, which give rise to colony-forming units (CFU-E) and express *Car2*. Notably, we see the same progression from *TRIB2*-expressing to *CA2*-expressing erythroid progenitors (Fig. 4c), suggesting the existence of the same two precursors subclasses in humans. In this regard, our data also confirm and expand the information on the divergence of human and mouse erythropoiesis^31^ (Fig. 4c). Of note, when analyzing the human–mouse orthologous that are differently expressed along the erythroid branch, we discovered that the most significant distinction is the expression of genes involved in the molecular apparatus supporting protein translation (Supplementary Fig. 10). This difference in the expression of the machinery of ribosome biogenesis during erythropoiesis could explain why mouse models of red blood cells disorders caused by a partial loss of ribosomal function, such as Diamond–Blackfan anemia, are not able to recapitulate the human phenotype^32^.

### Exploring CD164 as a marker of early human HSPC

**Fig. 5:**
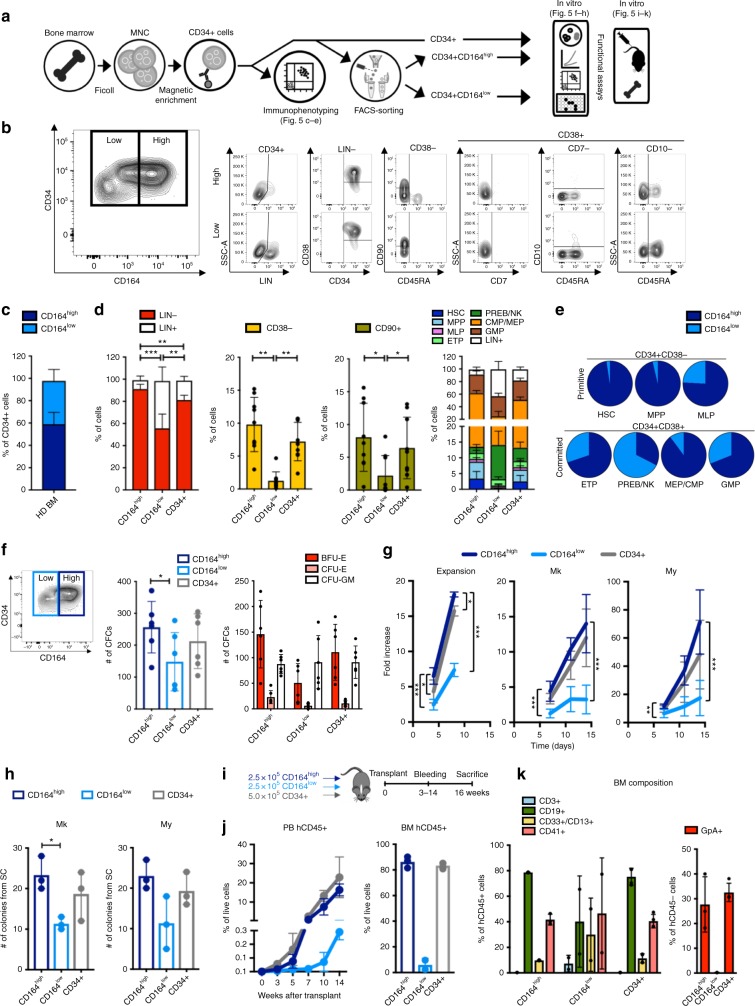
Immunophenotyping and in vitro/in vivo functional assays of CD164 expressing subsets in BM CD34+ cells. **a** Experimental design. **b** Representative FACS plots showing the contribution of Lin−/+ cells and HSPC subsets in CD164^high^ and CD164^low^ fractions of CD34+ cells. **c** Percentage of CD164^high^ and CD164^low^ fractions in CD34+ cells. Shown are Mean ± SD from nine independent BM. **d** Bar graphs showing the content of Lin−/+, CD38−, CD90+ cells and HSPCs in CD164^high^ and CD164^low^ fractions, and in CD34+ cells. Values are Mean ± SD from nine independent BM. Statistics by independent samples, heteroscedastic, two-tailed Student’s *t* test (*p < 0.05, **p < 0.0005, ***p < 0.0001). For the HSPCs bar graph, plotted values are proportion estimates ± SE, estimated using method of moments and Dirichlet-Multinomial model. Details are provided in Supplementary Table 4. **e** Pie chart distribution of CD164^high^ and CD164^low^ fractions on HSPC subsets from nine independent BM. **f** Bar graphs showing the total number (left) and type of colonies (right) scored at day 14 in a methylcellulose-based colony-forming unit (CFU) assay. Top left, sorting gating strategy (CFCs, colony-forming cells, BFU-E, burst-forming unit-erythroid cells, CFU-E, colony-forming unit-erythroid cells, CFU-GM,colony-forming unit-granulocyte/macrophages). Shown are mean ± SD from six independent BM. Statistics by independent samples, heteroscedastic, two-tailed Student’s *t* test (*p < 0.05). **g** Growth curves from three different culture conditions. Mk, Megakaryocyte; My, Myeloid. Values are mean ± SD from nine independent BM. Statistics by independent samples, heteroscedastic, two-tailed Student’s *t* test (*p < 0.05, **p < 0.0005, ***p < 0.0001). **h** Single-cell (SC) assay showing the total number of colonies obtained from each population in the Mk (left) and My (right) differentiating culture. Shown are median ± error from three independent BM. Statistics by independent samples, two-tailed Student’s *t* test (*p < 0.01). **i** Experimental design. Sorted CD164^high^ and CD164^low^ populations were transplanted in NBSGW mice each at the dose of 2.5 × 10^5^ cells/mouse. In order to reflect the real proportions in the human BM, immunomagnetic-selected CD34+ cells were transplanted at the dose of 5.0 × 10^5^ cells/mouse. The human engraftment was evaluated in the murine peripheral blood at different time points, and in BM and spleen at 16 weeks post transplant. **j** Human CD45+ cell engraftment in murine PB (left; CD164^high^, *n* = 3; CD164^low^, *n* = 3; CD34+, *n* = 4 mice) and BM (right; CD164^high^, *n* = 3; CD164^low^, *n* = 2; CD34+, *n* = 4 mice). **k** Relative contribution of human cell populations inside the hCD45+ and hCD45− compartments in murine BM. (CD164^high^, *n* = 3; CD164^low^, *n* = 2; CD34+, *n* = 4 mice)

We next asked whether we could take advantage of the data to rationally select a cell surface marker to fractionate human HSPCs for transplantation and gene therapy (Fig. 5). To date, the CD38 antigen has served to negatively enrich for the primitive progenitors for transplantation. Yet this marker suffers three shortcomings and thus motivated us to search for an alternative from the data. First, there is no consensus on the gating strategy to be used for CD38 expression to define CD38− primitive cells^33^, resulting in variable efficiencies of progenitor cell enrichment. Second, in strategies proposing a CD38− cells selection for transplantation, CD38+ myeloid progenitor cells (CMP and GMP) must be provided separately to support short-term granulopoiesis in conditioned neutropenic patients^33,34^. Third, we show here that expression of CD38 is rapidly lost in culture upon cytokine exposure (Supplementary Fig. 11), meaning that the viability and composition of early progenitors cannot be verified in transplantation products after in vitro expansion using the CD38− cytometric gating. We propose here that the cell surface antigen CD164 could be used to overcome all three of these shortcomings.

The CD164 gene encodes for a membrane-associated sialomucin, endolyn, whose function is that of an adhesion receptor^35^. The few investigations on the membrane expression of this protein in the human blood cell population suggest that CD164 could have a role in early erythropoiesis, stem cell maintenance and homing capacity^36,37^. An early study showed that the CD164 population is enriched in CD34+CD38− progenitors^38^, but following these investigations the use of CD164 for defining primitive progenitors was abandoned. Up until now, the expression of CD164 inside the currently defined HSPC subpopulations and upon in vitro manipulation of CD34+ cells, were not appreciated.

Interrogating scRNA-Seq data for enrichment of transcripts encoding for surface antigens in early progenitors (Fig. 1g), CD164 emerged as the gene whose expression displayed the most-pronounced difference in early vs late progenitors. By contrast, neither CD38 nor CD90 (common marker used for identification of primitive HSPCs) stood out as genes whose transcripts strongly discriminate between early vs late stages of blood cell fate commitment. Although mRNA abundances do not necessarily correlate with protein abundance, we found that the CD34+ population can be split into two sub-fractions on the basis of two clearly distinct levels of CD164 transcript abundances (Fig. 1h), which tracked fractionation by CD164 antibody-based sorting. The CD164 RNA is selectively expressed at high level not only in CD38− multipotent progenitors (as previous studies suggested) but also in CD90+ precursors (which in humans comprise both HSC and early CMP), in the most primitive fraction of MEP and to a lesser extent, in MLP (Fig. 1g). During later stages of commitment, the CD164 mRNA and protein surface expressions levels begin to diverge (e.g., in the CD34–CD164^high^ erythroid-committed cells).

### CD164 selects an alternative CD34+ cell product

To investigate the utility of CD164 role in fractionating early hematopoietic progenitors, we performed a series of immunophenotypic and functional assays on human BM CD34+ cells (Fig. 5a). In line with scRNA-Seq results, a cytometric analysis combining anti-CD164 antibody with the other classical HSPCs markers, confirmed that the CD34+ population contains two clearly distinct fractions of CD164^high^ and CD164^low^-expressing cells, the first of which was highly enriched in cells with cytometric markers of primitive progenitors, MEPs and early CMPs and, notably, was almost entirely depleted of pre-B-NK and Lin+ cells (Fig. 5b–e and Supplementary Fig. 12a). Importantly, we could show that this differential composition between CD164^high^ and CD164^low^ populations in the human BM is not merely owing to the differences in the relative CD34 surface expression or in the Lin+ cell content. Indeed, we obtained the same results upon analyzing CD34+ cells from G-CSF- and plerixafor-mobilized peripheral blood where the CD34 expression is uniform in both CD164^high^ and CD164^low^ cell fractions and where the contribution of the Lin+ population is negligible (Supplementary Figs. 13, 14). To date, the literature reports only the results of a clonogenic assay as test of the in vitro differentiation potential of CD34+CD164+ cells^38^. To integrate these data, we here conducted a set of functional tests on FACS-sorted CD34+CD164^high^ and CD34+CD164^low^ cells from the BM of several healthy donors (Fig. 5f–h). The CD34+CD164^high^ population displayed a superior in vitro differentiation potential as compared with the CD34+CD164^low^ fraction and even to the total CD34+ population, showing higher rate of colonies generation (confirming previously published results^38^) and of expansion not only in Myeloid but also in MK-differentiating conditions (Fig. 5f–h). Cytometric analysis of differentiation states after culture confirmed the more primitive nature of CD34+CD164^high^ cells (Supplementary Figs. 12, 15). At last, the CD34+CD164^high^ cells expanded more rapidly in culture conditions used in clinical gene therapy for in vitro stem cell enrichment prior to autologous transplantation^39^ (Fig. 5g). Importantly, in this context we observed that CD164 allows, as compared with CD38, a more robust cytometric estimation of the primitive progenitor content upon in vitro manipulation of CD34+ cells, as its loss of expression coincides with the progressive cell differentiation upon cytokine exposure (Supplementary Fig. 11). This is a major advantage over the use of the classical CD38 marker whose expression dynamics were instead not consistent with the expected phenotype changes of differentiating cells.

Another key surface marker used for the identification of stem/multipotent vs committed progenitor is CD90^40^. We thus conducted additional differentiation assays on three healthy donors comparing the performance of FACS-sorted CD34+CD90+ cells to CD34+CD164^high^ population. The results displayed in Supplementary Figs. 16 and 17 show that the CD34+CD164^high^ fraction has a much higher discriminatory potential, as compared with the CD34+CD90+ selection, for cells capable of growing in myeloid- and MK-differentiating conditions and for clonogenic progenitors (Supplementary Figs. 16g, 17c). Furthermore, as in the case of CD38, the CD90 marker presented inconsistent expression dynamics in culture, being upregulated (and not downregulated) upon cell differentiation (Supplementary Figs. 18–20), again pointing to the superior performance of CD164 in allowing a more reliable evaluation of the stem cell content of in vitro manipulated CD34+ cell products (Supplementary Fig. 21).

We have shown above that the CD34+CD164^high^ population contains both multipotent progenitors and early CMP. On the basis of the model of hematopoietic reconstitution emerging from our recent clonal tracking data in humans^6,41^, we reasoned that the CD34+CD164^high^ fraction might constitute a suitable self-sufficient cell product for transplantation that would not require the co-infusion of other cells to support recovery from neutropenia and early myelopoiesis. To test this hypothesis, we sorted and transplanted CD34+CD164^high^ vs CD34+CD164^low^ populations into NOD.Cg-Kit^W-41J^Tyr^+^Prkdc^scid^Il2rg^tm1Wjl^/ThomJ (NBSGW) mice (Fig. 5i–k, Supplementary Fig. 22). The results confirmed that the CD34+CD164^high^ cell product is capable of sustaining both the early and late phases of hematopoietic reconstitution, whereas the CD34+CD164^low^ population did not have a role in blood cell production at either stage, making its use in transplantation virtually dispensable. Remarkably and in line with this observation, the dynamics and size of human lymphoid and myeloid cells output in the mice infused with FACS-sorted CD34+CD164^high^ cells was comparable to the mice infused with CD34+ cells, despite the latter receiving twice the amount of cells. Overall, our data clearly highlight the biological relevance of the CD164 gene in early haematopoiesis, reviving the use of this marker for the study of human HSPC and setting the basis for exploring the potential use of the CD34+CD164^high^ fraction in clinical transplantation and gene therapy, where there is a high demand for reducing the production costs for genetic engineering.

## Discussion

We here report the generation of high-resolution scRNA maps of human hematopoietic cell fate commitment and the interrogation of our transcriptional profiling for conducting investigations into the basic biology of early hematopoiesis. Our fractionation strategy of the BM Lin− cells extended outside the CD34+ compartment constitutes a main advance over previous studies in that allowed us to preserve high resolution at both primitive and lineage-primed progenitors level. The results of the in silico, in vitro, and in vivo analyses reported in this work strongly suggests that human haematopoiesis develops along early cell fate bifurcations occurring in a continuum of states forming a hierarchical-like structure.

Our investigations into the origin of the basophil branch suggest that a very early priming of CD38− progenitors might be in place toward either the MK/erythroid/basophil or the lymphoid/granulo/DC/monocyte commitment and that this might be dependent on the expression of the CD135 surface marker. This observation calls for further studies into the potential heterogeneous composition of the CD34+CD38− compartment. In this regard, we would like to raise awareness on the arbitrary nature of the current strategies for the cytofluorimetric identification of the CD38− compartment. Indeed, despite using a very stringent CD38− sorting strategy we still observed an overlap of transcriptional states between CD38− HSC/MPP and CD38+ CMP/MEP (Supplementary Fig. 2a). This is owing to the continuum of CD38 expression, which does not provide a clear-cut way to isolate with high purity primitive progenitors. We therefore suggest that, upon validating potential early lineage priming of human HSC or MPP, one should commit to the use of an extremely conservative CD38− gate in order to obtain high purity of bona fide multipotent progenitors.

Our in vitro and in vivo data support the hypothesis that the CD34+CD164^high^ population might have a clinical relevance for transplantation purposes. Among the advantages of using such fraction of CD34+ cells in the clinic we would like to underline the following: (1) it excludes Pre-B precursors and CD34+ Lin+ cells (in large part composed by CD34+CD19+ cells), providing a system worth exploring for the potential exclusion of residual leukemic cells with early B-cell commitment in transplantation products for B-cell leukemia; (2) it could reduce of about a half the number of target cells needed for genetic engineering in clinical gene therapy, in turn reducing of 50% the costs for manufacturing of gene transfer/gene editing platforms. Notably, because it combines only two surface markers (CD34 and CD164), this fractionation method allows designing strategies based on magnetic beads selection, a more suitable and scalable approach for the clinical arena than FACS sorting. We also show here that this fraction might constitute a self-sufficient product capable of sustaining both early and late phases of hematopoietic reconstitution^41^, another advantage over the currently proposed selection strategies that would likely require co-transplantation of committed precursors to sustain early myelopoiesis^33,34^. Before suggesting its use in the clinic, further investigations are underway in our laboratory to test the safety and efficacy performance of the CD34+CD164^high^ population upon different in vitro manipulation protocols and after transplantation in multiple recipient animal models.

Despite the high-resolution achieved upon our scRNA profiling it should be reminded that such analytical method generates a static snapshot of the transcriptional landscape and cannot provide, as such, conclusive information on the dynamics occurring along cell state transitions. Ongoing and future efforts toward fate mapping in vitro and in vivo will be required to confirm or refine the inferences of our study. In this regard, our results in humans align with the ones of Tusi et al. in the mouse in supporting the hypothesis of an early separation of cells with erythroid vs neutrophil potential, a concept that would challenge some earlier deductions made in other studies of murine hematopoietic cell dynamics^8,42^.

In conclusion, we show here that the transcriptional information represented in our hierarchy fosters basic investigation into human hematopoiesis and enables the identification of human HSPCs subsets potentially suitable for clinical application.

## Methods

### Cell preparation

BM samples were collected from adult healthy donors at Children’s Hospital in Boston with the approval of the Committee on Clinical Investigations Children’s Hospital Boston and consent from the subjects under the protocol #09-04-0167. Mononuclear cells (MNCs) were isolated using Ficoll-Hypaque gradient separation (Lymphoprep, STEMCELL Technologies). CD34+ cells were purified from MNCs with the human anti-CD34 MicroBeads Isolation Kit (Miltenyi Biotec) according to the manufacturer’s specifications or were purchased from commercial sources (AllCells).

### Cell sorting and immunophenotyping

Seven HSPC subpopulations were purified from the CD34+ fraction of a healthy donor BM cells through a two-step four-way sorting using FACSAria II (BD Biosciences) and processed to generate the transcriptome network in Fig. 1. The following combinations of cell surface markers were used to identify and separate the HSPC subsets. Hematopoietic stem cells (HSC): Lin−CD34+CD38-CD90+CD45RA-; multipotent progenitors (MPP): Lin−CD34+CD38−CD90−CD45RA−; multi-lymphoid progenitors (MLP): Lin−CD34+CD38−CD90−CD45RA+; pre-B lymphocytes/natural killer cells (PREB/NK): Lin−CD34+CD38+CD7−CD10+; MEP: Lin−CD34+CD38+CD7−CD10−CD135−CD45RA−; CMP: Lin−CD34+CD38+CD7−CD10−CD135+ CD45RA−; GMP: Lin−CD34+CD38+CD7−CD10−CD135−CD45RA+.

For the generation of the transcriptome network in Fig. 2, four cell fractions were purified from a healthy donor BM MNCs through a four-way sorting using the following combinations of cell surface markers: Lin−CD34+CD164+; Lin−CD34^low^CD164^high^; Lin−CD34–CD164^high^; Lin−CD34–CD164^low^. CD71 was included to identify erythroid progenitors.

For in vitro functional assays, Lin−CD34+CD135− and Lin−CD34+CD135+ fractions were purified from the CD34+ cells of three independent BM through a two-way sorting. The cell subsets CD34+CD164^high^ and CD34+CD164^low^ were FACS-sorted from the CD34+ cells of nine independent BM. Of these, three BM were also used to purify CD34+CD90+ and CD34+CD90− cells.

For in vivo studies, CD34+CD164^high^ and CD34+CD164^low^ cells were FACS-sorted and purified from a pool of BM CD34+ cells from two additional healthy donors.

Immunophenotyping was performed on BM CD34+ cells labeled with CD135 or CD164 in combination with HSPC subsets markers by using LSR Fortessa (BD Biosciences). CD15 and CD19 were included to identify the lineage positive cells. Flow cytometry data were analyzed with FlowJo 10.2 (Tree Star). The antibodies were as follows: CD34 PB (1:40, #343512), CD38 PE/Cy5 (1:40, #303508), CD90 APC (1:33, #328114), CD10 PE/Cy7 (1:33, #312214), CD135 PE (1:10, #313306), Lin BV510 (1:10, #348807), CD15 BV510 (1:50, #323028), CD164 FITC (1:20, #324806 clone 67D2), CD164 PE (1:10, #324808 clone 67D2), CD71 PerCP/Cy5.5 (1:20, #334114), CD41 APC (1:20, #303710), CD19 PE/Cy7 (1:20, #302216), all Biolegend. CD45RA APC-H7 (1:17, #560674), CD7 AF700 (1:20, #561603), CD15 FITC (1:20, #555401), CD15 PE (1:10, #555402), all BD Biosciences. Glycophorin A APC-Vio770 (1:11, #130-100-268), Miltenyi Biotec.

To characterize the basophils contribution in the human peripheral blood and upon in vitro differentiation, the gating strategy reported in Supplementary Fig. 9a has been set using the following antibodies: CD34 PB (1:40, #343512), FceRIA APC (1:10, #334612), CD14 AF700 (1:10, #367114), CD19 PE/Cy7 (1:20, #302216), CD15 FITC (1:20, #555401), CCR3 PerCP/Cy5.5 (1:10, #310718), all Biolegend. IL5RA PE (1:10, #555902), BD Biosciences.

To evaluate the human cell engraftment in the murine peripheral blood, BM and spleen the antibodies were as follows: CD33 PE (1:40, #561816), CD13 PE (1:40, #555394), CD3 V500 (1:20, #561416), CD19 PE/Cy7 (1:80, #557835), mCD45 APC (1:100, #561018), mCD45 PE (1:100, #553081), 7-AAD (1:12, #559925), all BD Biosciences. CD45 PB (1:40, #368540) and CD41 APC (1:50, #303710) all Biolegend. Glycophorin A APC-Vio770 (1:22, #130-100-268), Miltenyi.

### In vitro functional assays

For the in vitro functional assays, sort-purified populations and CD34+ cells were seeded in the different culture conditions with a starting cell number of 20,000 cells, unless otherwise indicated.

To test for basophil potential, cells were cultured in Iscove's Modified Dulbecco's medium (IMDM) containing 1% P/S/Glu, 20% FBS (Gemini) and supplemented with IL-3 (20 ng/ml), IL-5 (20 ng/ml), SCF (20 ng/ml), GM-CSF (50 ng/ml) for 3 days, whereas supplemented only with IL-3 (20 ng/ml) and IL-5 (20 ng/ml) from day 4 to day 14. Cells were counted on days 7,11,14. Fresh medium was added as needed, to keep the cell concentration at 0.5 × 10^6^/mL. At the end of the culture, cells were analyzed by flow cytometry for the basophil markers and mounted on cytospin preparation to define the presence of basophils by Giemsa staining.

Myeloid potential was evaluated in IMDM medium containing 1% P/S/Glu and 10% FBS (Gemini) and supplemented with IL-3 (60 ng/ml), SCF (300 ng/ml), IL-6 (60 ng/ml) for 2 weeks. Cells were counted on days 7,11,14. Fresh medium was added as needed, to keep the cell concentration at 1 × 10^6^/mL. At the end of the culture, cells were analyzed by flow cytometry for immunophenotyping and lineage-positive markers CD15 and CD19, and for basophil markers.

Expansion culture was set up in serum-free CellGro SCGM medium (Cell Genix) containing 1% penicillin/streptomycin/glutamine (P/S/Glu, Lonza) and supplemented with FLT3-L (300 ng/ml), IL-3 (60 ng/ml), SCF (300 ng/ml), TPO (100 ng/ml) for 8 days. Cells were counted on days 4 and 8. Immunophenotyping and flow cytometric analysis for lineage-positive markers CD15 and CD19 were performed at day 4. All growth factors and cytokines were purchased from Peprotech.

Megakaryocyte potential was assessed in StemSpan SFEM II serum-free medium supplemented with StemSpan Megakaryocyte Expansion Supplement (STEMCELL Technologies) for 2 weeks. Cells were counted on days 7,11,14. Fresh medium was added as needed, to keep the cell concentration at 1 × 10^6^/mL. Immunophenotyping and flow cytometric analysis for CD41, CD71, and Glycophorin A were performed at the end of the culture.

To test the clonogenic potential of sort-purified populations and CD34+ cells, single-sorted cells were deposited in 96-well plates in different culture conditions. Medium was added at day 7 and colonies were scored at day 14. From CD34+ cells and each freshly sorted CD164^high^ and CD164^low^ populations, the clonogenic potential was also assessed by seeding 3500 cells with 2.4 ml of Methocult medium (H4434, STEMCELL Technologies) for 2 weeks. Erythroid (BFU-E or CFU-E) and granulocyte–macrophage (GM) colonies were scored from duplicate plates on day 14.

### Transplantation into humanized mouse model

NOD.Cg-Kit^W-41J^Tyr^+^Prkdc^scid^Il2rg^tm1Wjl^/ThomJ (NBSGW) mice were purchased from the Jackson Laboratory. All animal procedures were performed according to ethical regulations for animal testing and research, upon approval by the Institutional Care and Use Committee (IACUC) at the Dana-Farber Cancer Institute. Six-week-old mice were transplanted with human HSPCs by tail injection without undergoing irradiation or other conditioning regimen. Mice were randomized in the following transplantation groups: sorted purified CD34+CD164^high^ (2.5 × 10^5^ cells/mouse) and CD34+CD164^low^ (2.5 × 10^5^ cells/mouse), immunomagnetic-selected CD34+ (5 × 10^5^ cells/mouse). For each sorted population, three mice were transplanted (four mice for the whole CD34+ population). Human cell engraftment was assessed by serial bleeding and immunophenotyping at 3, 5, 7, 10, 14 weeks post transplant and in BM and spleen at sacrifice 16 weeks post transplant. The CD34+CD164^high^ selection method is under provisional Patent Application U.S. Serial No.: 62/737,483 filed in the United States Patent and Trademark Office (USPTO).

### InDrops scRNA-Seq and data analysis

Single-cell mRNA barcoding and preparation of libraries for sequencing were performed following the inDrop protocol previously described in Zillionis et al.^22^, with modifications as described for the FACS subsets samples in Tusi et al.^24^. FACS-sorted subpopulations were individually processed for droplet barcoding (Supplementary Table 1). Emulsions were split in aliquots each containing ~ 2500 single-cell barcoded transcriptomes. Libraries generated from each FACS sorting were prepared in parallel and sequenced on Illumina NextSeq 500 using a NextSeq High Output 1 × 75 cycle kit. Raw sequencing data (FASTQ files) were processed using the previously described inDrops.py bioinformatics pipeline^24^ (available at https://github.com/indrops/indrops). Bowtie v.1.1.1 was used with parameter -e 100. All ambiguously mapped reads were excluded from analysis and reads were aligned to the Ensemble GRCh38.85 version of human genome.

### Cell filtering and data normalization

Each library of sorted HSPCs or CD34/CD164 cells was processed according to the following procedure. Upon inspection of the histograms reporting the total reads per cell, barcodes were initially filtered according to a customized threshold in order to include only the most abundant ones (transcript counts threshold used for the sorted HSPC: HSC, 1000; CMP, 800; MEP, 1000; GMP, 1000; Pre-B-NK, 800; MLP, 2000; MPP, 2000; transcript counts threshold used for the sorted CD34/CD164 cells: Lin−CD164^high^ CD34^low^Rep(Replicate)1, 1000; Lin−CD164^high^CD34^low^Rep2, 1000; Lin−CD164^high^CD34-Rep1, 800; Lin−CD164^high^ CD34-Rep2, 800; Lin−CD164^high^ CD34+ Rep1, 1000; Lin−CD164^high^ CD34+ Rep2, 800; Lin−CD164^low^CD34-, 700). Next, for all samples we excluded the cells with > 25% of their transcripts coming from mitochondrial genes as this is a marker of stressed or dying cells. The final number of barcodes used in the downstream analysis is summarized in Supplementary Table 1. The gene expression counts of each cell were normalized using a total-count normalization variant that avoids distortion from very highly expressed genes, as in Klein et al.^43^. Specifically, we calculated $\hat x_{i,j}$, the normalized transcript counts for gene *j* in cell *i*, from the raw counts *x*_*i,j*_ as follows: $\hat x_{i,j} = x_{i,j}\bar X/X_i$, in which $X_i = {\sum} {x_{i,j}}$and $\bar X$is the average of *X*_*i*_ over all cells. To prevent very highly expressed genes from correspondingly decreasing the relative expression of other genes, we excluded genes comprising > 5% of the total counts of any cell when calculating $\bar X$ and *X*_*i*_.

### Data visualization and *k*NN graphs

After filtering, the data were used to construct a *k*-NN graph, in which cells correspond to graph nodes and edges connect cells to their nearest neighbors. An independent *k*NN graph was generated for each data set as follows. Genes were further filtered by selecting only genes with Fano factor^43^ (measure of dispersion) above a mean dependent threshold (median value) and requiring at least three UMIFM (Unique Molecular Identifiers Filtered Mapped) to be detected in at least three cells (sorted Lin−HSPCs, *n* = 5596 genes; sorted Lin−CD34/CD164 cells, *n* = 7156 genes). Expression values for each gene were standardized independently by applying *Z* score transformation. Unless otherwise stated, for all the analyses and graphical representations throughout the paper, *z* scores have been used as a measure of gene activity. From previous experiments^24^, we found that cell cycle and ribosomal associated genes can have a significant impact on the definition of cell clustering and on cell-to-cell transcriptional distance. For this reason, we defined a G2/M genes set (*UBE2C, HMGB2, HMGN2, TUBA1B, MKI67, CCNB1, TUBB, TOP2A, TUBB4B*) and ribosomal genes set (*RPL− and RPS−*). We then constructed a G2/M and a ribosomal signature score by summing the average *z* score of respective genes sets and removing genes that were highly correlated (Pearson *r* > 0.2) with these signatures (sorted Lin−HSPC, *n* = 117 genes; sorted CD34/CD164 cells, *n* = 304 genes). Finally, we performed dimensionality reduction by principal component analysis (PCA). *K*NN graphs were constructed by setting *k*, number of neighbors, equal to four, using the first 40 principal components and a Euclidean metric to measure distance between transcriptomes. The *k*NN graphs were visualized by means of a force-directed layout using the custom interactive software interface SPRING^25^. The final layout, corresponding to a minimal free-energy configuration, showed a high degree of robustness with respect to different initialization (except for layout rotation that do not affect subsequent analyses). No manual adjustments were performed on the visualizations. Visual inspection on SPRING plot for Lin−CD34/CD164 transcriptome data set showed the presence of a cluster of cells (860 barcodes), highly interconnected and very poorly linked to the rest of the layout. Investigating for the presence of a particular gene expression signature characterizing this subpopulation, we observed high level of expression for mitochondrial genes (MT.CYB, MT.ATP6, MT.ND4, MT.ND1, MT.CO3, MT.ND3). We concluded that these events had a peculiar transcriptional profile indicator of stressed or dying cells, which was not detected upon the dedicated filtering step and we therefore manually removed them from the final *k*NN graph.

### Projection of scRNA data across experiments

To project subsets of cells from one map to the other, we first needed to define a common lower dimensional space to be used as reference to compare expression profiles, calculate transcriptional distances and locate cells with an high degree of similarity among the two maps. For this reason, we identified the intersection set among genes used to generate the two *k*NN graphs (*n* = 5116 genes). Given that Lin−CD34/CD164 map represents a broader view on Lin− compartment with respect to the sorted Lin−HSPC data set, we performed PCA on Lin−CD34/CD164 reduced expression matrix retaining the first 40 principal components. Sorted Lin−HSPCs were projected on the Lin−CD34/CD164 principal component space upon *z* score transformation of genes expression data with gene specific centering and scaling parameters derived from Lin−CD34/CD164 data. With this procedure we obtained a common 40-dimensional support that allows for a direct comparison among transcriptome data derived from the two experiments. For each cell belonging to a specific group (FACS-sorted subpopulation or computationally identified group) in either sorted Lin−HSPC or Lin−CD34/CD164 map, we identified the *k* = 4 most similar cells in the other map, using PCA scores and Euclidean distance. The graphical representations in Fig. 2f and Supplementary Fig. 8 show sorted HSPC cell groups projection into Lin−CD34/CD164 map and have been generated by rescaling the two-dimensional Lin−CD34/CD164 SPRING layout to a unit squared area. We calculated cell spatial distribution using a two-dimensional kernel density estimator (bandwidths for *x* and *y* directions both set to 0.035) and use a contour plot for density level 1e-05 to highlight areas characterized by a non-negligible probability. We then overlaid a density estimation (bandwidths for *x* and *y* directions both set to 0.1) for the spatial distribution of cells selected as most similar. In Fig. 3a, Lin−CD34/CD164 group 9 have been projected into sorted Lin−HSPC layout. Settings used to generate graphs have been kept equal to those aforementioned. In addition we also investigate the immunophenotypic distribution for sorted Lin−HSPCs, reported as piechart in Fig. 3a

### Observed and adjusted cell-density estimations

The transcriptional state related to small subpopulations such as the most-primitive ones, are difficult to investigate by means of single-cell profiling on bulk heterogeneous populations. Introducing a fractionation strategy through FACS sorting before inDrops barcoding, we were able to overcome this limitation by artificially over-representing primitive fractions inside the CD34+ and Lin− compartments. This aspect is shown in the two-dimensional density estimation plotted in Supplementary Fig. 1a, b (left plots) where high-density values can be found in graph areas associated to both primitive and committed cells. To provide a representation of what would have been instead the expected contribution of single cell events to the bulk human CD34+ and Lin− population we assigned to each cell a weight, defined according to the proportion of events observed in the corresponding FACS gate. The graphs of Supplementary Fig. 1a, b (right), show densities obtained by keeping cells location constant and taking into account the calculated cell weights. Details for the calculation of weights are provided in the Supplementary Table 2.

### Transcriptional principal trajectories identification

Both the topologies generated with SPRING reveal the presence of a continuum of transcriptional states connecting the most primitive subpopulations to more committed ones. Although some degree of variability is observed, layout topologies also suggest the presence of principal transcriptional trajectories during the differentiation process. We considered that the estimation and characterization of these trajectories could potentially allow us to: (a) establish an order among transcriptional states with respect to differentiation process; (b) group together cells with a common fate; (c) investigate the gene regulatory dynamics underlying fate decision and lineages commitment. For these purposes, we implemented a procedure composed by the following main steps: (1) structure-aware filtering performed on transcriptome graph; (2) branching reconstruction by minimum spanning tree on reduced consolidating points; (3) association and ordering of cells according to inferred branching structure. To follow the description of these steps.

(1) Structure-aware filtering. The structure-aware technique that we adopted in this paper work is aimed to at revealing and consolidating continuous, low-dimensional, and high-density structures in the underlying higher-dimensional data, whereas ignoring noise and outliers. The theory, proof of convergence to the exact underlying data manifolds (under Gaussian noise assumption) and an investigation of its performance under different scenario can be found in Wu et al.^44^. Here we will briefly describe its discretized version formulation, i.e., representing densities by sets of sample points. Observed data points, *p*_*i*_, are considered sampled from an underlying *n*-dimensional density *f*_*p*_*(z)*, supposed to have been generated by adding noise to an underlying lower (*m* < *n*) *m*-dimensional data manifold. Consolidation points, **x**_*i*_*(t)*, are considered to be sampled from a time-dependent distribution *f*_*x*_*(***z***,t)*, initialized as *f*_*x*_*(***z***,0)* = *f*_*p*_*(***z***)*, that changes over time (iterations) guided by a time-dependent velocity field that gradually remove noise while revealing the underlying *m*-dimensional structure in the input density *f*_*p*_*(***z***)*. Initially, consolidation points can be either a random sample of data points or, as we did, the whole data set. Although data points are fixed in the *n*-dimensional manifold, the position of every consolidation point is iteratively updated according to the following formula:

$${\bf{x}}_i\left( {t + 1} \right)\prime =\, {\bf{x}}_i\left( t \right)\frac{{\mathop {\sum }\nolimits_j {\mathbf{p}}_j{\mathrm{K}}\prime \left( {\frac{1}{2}\left\| {{\bf{p}}_j - {\bf{x}}_i\left( t \right)} \right\|^2} \right)}}{{\mathop {\sum }\nolimits_j {\mathrm{K}}\prime \left( {\frac{1}{2}\left\| {{\bf{p}}_j - {\bf{x}}_i\left( t \right)} \right\|^2} \right)}} \\ - \mu \frac{{\mathop {\sum }\nolimits_k {\bf{A}}\prime {\bf{A}}\left( {{\bf{x}}_k\left( t \right) - {\bf{x}}_i\left( t \right)} \right){\mathrm{L}}\prime \left( {\frac{1}{2}\left\| {{\bf{A}}\left( {{\bf{x}}_k\left( t \right) - {\bf{x}}_i\left( t \right)} \right)} \right\|^2} \right)}}{{\mathop {\sum }\nolimits_k {\mathrm{L}}\prime \left( {\frac{1}{2}\left\| {{\bf{A}}\left( {{\bf{x}}_k\left( t \right) - {\bf{x}}_i\left( t \right)} \right)} \right\|^2} \right)}}$$

where K′ and L′ are first derivatives of a standard and modified Gaussian smoothing kernels defined as

$${\mathrm{K}}\prime \left( {\frac{1}{2}\left\| {{\bf{p}}_j - {\bf{x}}_i\left( t \right)} \right\|^2} \right) = e^{ - \left( {\left\| {{\bf{p}}_j - {\bf{x}}_i\left( t \right)} \right\|^2} \right)/2r^2}$$

and

$${\mathrm{L}}\prime \left( {\frac{1}{2}\left\| {{\bf{A}}\left[ {{\bf{x}}_k\left( t \right) - {\bf{x}}_i\left( t \right)} \right]} \right\|^2} \right) = \frac{{e^{ - \left( {\left\| {{\bf{A}}\left[ {{\bf{x}}_k\left( t \right) - {\bf{x}}_i\left( t \right)} \right]} \right\|^2} \right)/2r^2}}}{{\left( {\left\| {{\bf{A}}\left[ {{\bf{x}}_k\left( t \right) - {\bf{x}}_i\left( t \right)} \right]} \right\|^2} \right)}};$$

*j* and *k* mark, respectively, data and consolidation points within a radially symmetric, *n*-dimensional neighborhood of user-defined radius *r* centered on **x**_*i*_(*t*); 0 < *μ* < 1 is a user-defined constant; ${\bf{A}}_i = \left[ {\lambda _i^1v_i^1;\lambda _i^2v_i^2; \ldots ;\lambda _i^mv_i^m} \right]$ is a *n* × *m* matrix with $\{ \lambda _i^1;\lambda _i^2; \ldots ;\lambda _i^m\}$ and $\{ v_i^1;v_i^2; \ldots ;v_i^m\}$, respectively, first *m* eigenvectors and eigenvalues of the *k* × *n* matrix in which the *k-*th row is equal to the n-dimensional vectors **x**_*k*_(*t*) − **x**_*i*_(*t*). The iterative procedure continues until the sum of consolidation points displacement, ${\mathrm{\Delta }}{\bf{X}} = {\sum} {[{\bf{x}}_i\left( {t - 1} \right) - {\bf{x}}_i\left( t \right)]^2}$ is greater than a given small ${\it{\varepsilon }}$ (0.001). In the updating formula it is possible to recognize two components: the first one, called the data-term, pulls consolidation points toward local extrema (high-density regions) of the noisy input density. The second, called repulsion-term, prevents clumping of consolidation points by pushing them along locally optimal directions, enhancing latent continuous m-dimensional structures. A graphical representation is given in Supplementary Fig. 23a. In this work, we performed structure-aware filtering on the two-dimensional representation of SPRING generated layouts, upon rescaling to the unit square two-dimensional space as previously described. The goal was to highlight the underlying one-dimensional (curves) representations (*m* = 1). In general, given a value for radius size *r*, it returns an estimated optimal structure providing an accurate representation of data layout complexity and allowing for an interpretation in biological terms. In their paper^44^, authors suggest a method to help user in setting this critical parameter. Under the assumption of Gaussian distributed input data with known variance, this method estimates a lower bound for *r* able to guarantee convergence to the true *m*-dimensional manifold. We chose algorithm input parameters complying with these indications, setting, respectively, for the sorted Lin−HSPC and sorted Lin−CD34/CD164 cell graphs: *r* equal to 0.05 and 0.02; *μ* equal to 0.3 for both. To ensure reproducibility of the results, we initialized the set of consolidation points with the whole set of data points. In Supplementary Fig. 3, initial, temporary (2nd and 10th iterations) and the final configurations are shown.

(2) Branching reconstruction by minimum spanning tree on reduced consolidating points. Structure-aware filtering returns coordinates of consolidation points in the *n*-dimensional input space such that they describe a continuum of locally optimal m-dimensional structures. In order to infer the principal transcriptional trajectories, we proceed as follows. We first reduced the set of consolidation points by iteratively averaging points closer than 0.01 (Supplementary Fig. 3, Merging plots). This step has a regularization goal and allows for a considerable reduction of the data set size for downstream analyses. To connect points and design the graph skeleton, we opted for the minimum spanning tree algorithm, with Euclidean distance based edges weighting. Only in the sorted HSPC analysis we left unconnected the small cluster located between erythroid and neutrophils due to its large distance from others consolidation points. The minimum spanning tree on reduced points is visible in Supplementary Fig. 3, MST plots.

(3) Branch association and cells ordering. Through the identification of bifurcation nodes, we subdivided the minimum spanning tree in segments (or trajectories, or branches) as shown in Supplementary Fig. 3, Principal trajectories plots. Each cell has been associated to one segment, based on minimum distance criteria. In order to exclude cells with a transcriptional profile too different from those captured by the principal trajectories, cells more distant than 0.05 from any of the branches remained unlabeled. To order cells along the corresponding trajectory, we calculated the distance between the initial node (marked with 0 in Supplementary Fig. 23b) and the projection of each cell onto the trajectory. Rescaled distances (0–1 interval), have been calculated and used as pseudotime values in all gene expression analyses described in the next session and discussed in the manuscript.

All the algorithms have been implemented in R^45^ and are made available for download at https://github.com/BiascoLab/PrincipalDevelopementalTrajectories.

### Generation of Diffusion map

In order to verify the robustness of our results with respect to the adopted data analysis approach, we compared the Lin−CD34/CD164 *k*NN-based transcriptome topology and inferred differentiation trajectories to those derived from an alternative method, not relying on *k*NN, such as Diffusion map^27^. We took advantage of R implementation of diffusion map available in package destiny^46^, that is specifically designed for scRNA-seq data. We passed as input argument to DiffusionMap function the matrix reporting the 40 principal components representation of filtered-and-normalized expression data, obtained as described in the Data visualization and construction of *k*-nearest neighbors graphs section. All other DiffusionMap settings have been kept to default configurations. The diffusion map for Lin−CD34/CD164 is shown in Supplementary Fig. 5. We confirmed the transcriptional principal trajectories identified starting from SPRING layout (Fig. 2d) by applying our algorithm to the three-dimensional diffusion map. The results are reported in Supplementary Fig. 6.

### Gene expression analysis

Throughout the manuscript, different types of gene expression analysis have been shown. The statistical model underlying each of them has been defined according to the specific question of interest. The analyses can be grouped in the following categories with related examples shown in Supplementary Fig. 23c: (1) differentially expressed genes across cell groups (Fig. 1c; Supplementary Fig. 5a, c); (2) identification of genes with a significant association between expression level and branch-specific pseudotime ordering (Fig. 3b, c; Supplementary Fig. 7); (3) investigation of differences in gene expression dynamics among trajectories (Fig. 2e; Supplementary Fig. 5b). Similarly to what proposed in Trapnell et al.^47^, we opted for a Generalized Additive Models^48^ approach, that allows to test the dependence between the response variable and different types of predictors in a more flexible manner. For example, by estimating regression coefficients by using different loss functions (M-estimators) or by modeling trend with nonparametric functions. To prevent the potentially high impact of expression value outliers and dropouts, frequently observed in single cell RNA-Seq data, in all fitting procedures we employed the Huber loss function for regression. Huber loss function is commonly used in robust regression and consists in a piecewise penalty function in which a quadratic penalization is replaced by a linear one for large differences. Its tuning constant has been set to *k* = 0.862, meaning that the linear loss is applied to differences below the 10th and above 90th percentile, assuming a central Gaussian part of the distribution of residuals.

Differentially expressed genes across cell groups have been identified by fitting and comparing the two following models for each gene separately. The full model assumes gene expression averages to be group-dependent. From a practical view-point, model likelihood and coefficients have been calculated by using group labels $G_i,i = 1, \ldots,k$, where *k* is the number of groups, as dummy variables, ${\mathrm{M}}_1:{\mathrm{\mu }}\left( Y \right) = {\mathrm{\beta }}_0 + {\mathrm{\beta }}_1G_1 + \ldots + {\mathrm{\beta }}_{\mathrm{k}}G_k$, where μ(*Y*) is the average expression value for a gene. The restricted (null) model ${\mathrm{M}}_0:{\mathrm{\mu }}\left( Y \right) = {\mathrm{\beta }}_0$ instead, assumes no-differences in mean expression values among groups and considers variation only owing to the intrinsic noise of expression measurements. Derived from this analysis are heatmaps in Fig. 1g and Supplementary Fig. 5a,c where statistically significant genes within specific subsets (CD marker genes^49^, Human and Mouse transcription factors^50^, blood cancer associated proto-oncogenes^51^) are shown. Information regarding all significant genes are available in Supplementary Information. Detection of genes that significantly change as a function of pseudotime, *t*, has been done by comparing the likelihood of the model ${\mathrm{M}}_1:{\mathrm{\mu }}\left( {Y,t} \right) = {\mathrm{\beta }}_0 + {\mathrm{s}}\left( t \right)$, where expression value trend μ(*Y*, *t*) varies according to a cubic splines (with four degree of freedom), s(*t*), to a flat null hypothesis ${\mathrm{M}}_0:{\mathrm{\mu }}\left( {\it{Y}} \right) = {\mathrm{\beta }}_0$ in which expression is assumed to randomly fluctuates around a constant value along the whole branch. All genes have been tested for association with respect to each branch and all estimated regression functions are available in Supplementary Information. Panels in Fig. 3b, c and Supplementary Fig. 7 are based on this modeling approach. Finally, to find differences in gene expression dynamics underlying fate decisions and divergent differentiation trajectories, we proceed as follows. As aforementioned, cell pseudotime value can be interpreted as a measure of cell degree of maturation along a specific segment of the differentiation process. Even though it is difficult to make a direct comparison among the regulatory dynamics underlying commitment toward different lineages, by rescaling the branch total length to the unit interval, it is possible to test whether a gene behaves differently among branches. This is a simplistic approach that only partially takes into account the potential presence of different maturation paces or other confounding factors such as varying duplication/differentiation/death rates. In the formulation of the full model employed in this gene expression analysis, we also assumed that cells belonging to trajectories stemming from a common bifurcation node, exhibit an expression pattern highly similar for pseudotime values close to 0, that will then eventually progressively diverge toward more branch-specific transcriptional states. This assumption motivated the formulation of the model ${\mathrm{M}}_1:{\mathrm{\mu }}\left( {Y,t} \right) = {\mathrm{\beta }}_0 + {\mathrm{s}}_{\mathrm{i}}\left( t \right)G_i + {\mathrm{s}}_{\mathrm{j}}\left( t \right)G_j$, in which branch-specific gene regression curves can evolve according to distinct pseudo-temporal dynamics s_i_(*t*) and s_j_(*t*), constrained to have the same expression value for *t* = 0 (common intercept). The reduced model ${\mathrm{M}}_0:{\mathrm{\mu }}\left( {Y,t} \right) = {\mathrm{\beta }}_0 + {\mathrm{s}}\left( t \right)$, allows gene expression average to vary over pseudotime according to a non-linear function, but assumes a common s(*t*) for both groups. In Fig. 2e, Supplementary Fig. 5b and tables in Supplementary Information significant fate associated genes are reported. Transcription factors shown in Fig. 2e, Supplementary Fig. 5b have been selected (among those significant) because already proposed in the literature has correlated with lineage committed.

In all cases, the differences in explanatory power between M_1_ and nested model M_0_, have been tested by Chi-squared likelihood ratio test (LRT). Statistic value, along with associated *p* values, gene name, and estimated mean/regression curves are reported in Supplementary Information only for those genes with adjusted *p* value *α* < 0.05 (Holm method^52^ for multiple comparisons). All the analyses have been performed by means of custom R^45^ scripts available at https://github.com/BiascoLab/PrincipalDevelopementalTrajectories. For regression fitting and model testing, we used the VGAM library^53^, and in particular vgam(), huber1() and sm.bs(), respectively, for estimate, loss function and splines interpolation and lrtest() for testing.

### Comparison of human vs mouse erythropoiesis

In order to compare the gene expression dynamics associated to human and mouse erythropoiesis, we took advantage of data generated by using inDrops technology on mouse Kit+ cells^24^. For mouse data set, differentiation trajectories were identified and cells labeled (Fig. 3a) according to the methodology afore described. We considered as representative of erythroid commitment subgroup 6 in mouse and subgroups 6, 7, 8 in human Lin−CD34/CD164 map (Fig. 3b top). Genes were tested for association to pseudotime in the two organisms separately (human: 3821; mouse: 1071 statistically significant genes, LRT adjusted *p* value < 0.05; complete lists and details are available in Supplementary Information). Among those significant, we retrieved 720 orthologous genes based on Mouse Genome Database (MGD)^54^ (Mouse Genome Informatics website, The Jackson Laboratory, Bar Harbor, Maine, http://www.informatics.jax.org), for which behavior is plotted by means of symmetric heatmap in Fig. 3b (bottom). We further investigated dis/similarities calculating Pearson correlations coefficients for each couple of human/mouse homologous genes (Supplementary Fig. 10, table available in Supplementary Information), and performed pathway enrichment analysis using Reactome database^55^ on the 89 human genes exhibiting a low-or-negative correlation (Pearson correlation < 0.5).

### Population balance analysis

To infer the structure of the hematopoietic lineage tree from the scRNA-seq data, we applied PBA (Weinreb et al.^56^) and calculated couplings between each pair of fates. For the Lin−HSPC subset data set, PBA was run on the merged data, using the *k*NN graph constructed as above (Data visualization and construction of *kNN* graphs). PBA was run as in Tusi et al.^24^. In brief, we assigned negative values of *R* (the local imbalance between cell division and cell loss) to the five cells with highest gene signature score for each fate (see next paragraph) and a single positive value to the remaining cells such that $\mathop {\sum}\nolimits_i {R_i = 0}$. Setting the diffusion constant to 1, we fit the exit rates for each fate such that the five cells with highest HSC signature had average fate probabilities within 1% of uniform. A similar procedure was carried out for the Lin−CD34/CD164 data set. Here, we restricted the analysis to the CD164^high^CD34^+^ population, as this contained all uncommitted progenitors and the earliest unilineage progenitors. The *k*NN graph for PBA was constructed setting *k* to 40 to improve the robustness of the analysis, and we set the diffusion constant to 0.5 and used 10 cells per fate and 10 HSCs to fit the exit rates.

We used the following gene sets to define the lineage-specific signatures for the Lin−HSPC subset data set:

- Meg: *ITGA2B, PF4, VWF*
- E: *CA1, HBB, KLF1, TFR2*
- DC: *CCR2, IRF8, MPEG1*
- G: *ELANE, MPO, LYZ, CSF1R, CTSG, PRTN3, AZU1*
- Ly1: *RGS1, NPTX2, DDIT4, ID2*
- Ly2: *DNTT, RAG1, RAG2*
- HSC: *CRHBP, HLF, DUSP1*
  And for the Lin−CD34/CD164 data set:
- E: *KLF1, CA1*
- Meg: *ITGA2B, PLEK*
- BEM: *CLC, CPA3, HDC*
- Ly: *DNTT, CD79A, VPREB1*
- DC: *IRF8, SPIB, IGKC*
- M: *LYZ, MS4A6A, ANXA2*
- N: *ELANE*
- HSC: *HLF, ADGRG6, CRHBP, PCDH9*

For both data sets, we used the PBA-predicted fate probabilities to infer a differentiation hierarchy, as in Tusi et al.^24^ (Figs 1f, 2c). A fate coupling score (see next paragraph) was computed for each pair of fates, and pairs with scores significantly higher than expected under the null model were joined and their fate probabilities merged by addition. This process was carried out iteratively until all fates were joined.

The coupling score between two fates *A* and *B* is the number of cells with P(*A*)P(*B*) > *ε*, using *ε* = 1/14 throughout. We generated a null distribution for each pair of fates by computing the coupling scores for 1000 permutations of the original fate probabilities, re-normalizing each cell’s probabilities at each randomization. The significance of the observed couplings was measured using the *z* score with respect to the null distribution.

### Reporting summary

Further information on research design is available in the Nature Research Reporting Summary linked to this article.

## Supplementary information

Supplementary Information

Reporting Summary

Source Data

## Data Availability

Raw data are available with GEO accession code GSE117498 [https://www.ncbi.nlm.nih.gov/geo/query/acc.cgi?acc=GSE117498]. SPRING plots are available for inspection at the following links: Mouse Kit+, [https://kleintools.hms.harvard.edu/tools/springViewer_1_6_dev.html?datasets/mouse_HPCs/basal_bone_marrow/full]; Human Lin− CD164/CD34, [https://kleintools.hms.harvard.edu/tools/springViewer_1_6_dev.html?datasets/CD34_CD164/CD34_CD164]; Human sorted HSPC, [https://kleintools.hms.harvard.edu/tools/springViewer_1_6_dev.html?datasets/sortedHSPC/sortedHSPC]. The source data underlying Figs. 1g, 2e, 3, 4b,c, 5, Supplementary Figs. 7, 10, 12, 13, 15–17, 21–23 are provided as a Source Data File.

## Code availability

SPRING software is available at https://github.com/AllonKleinLab/SPRING. PBA algorithm is available at https://github.com/AllonKleinLab/PBA. Structure-aware data consolidation algorithm described in Wu, Shihao et al.^44^ has been implemented in R is available at https://github.com/BiascoLab/PrincipalDevelopementalTrajectories.

## References

[1] Kawamoto H, Ikawa T, Masuda K, Wada H, Katsura Y (2010). A map for lineage restriction of progenitors during hematopoiesis: The essence of the myeloid-based model. Immunol. Rev..

[2] Ema H, Morita Y, Suda T (2014). Heterogeneity and hierarchy of hematopoietic stem cells. Exp. Hematol..

[3] Eaves C (2015). Hematopoietic stem cells: concepts, definitions, and the new reality. Blood.

[4] Laurenti E (2013). The transcriptional architecture of early human hematopoiesis identifies multilevel control of lymphoid commitment. Nat. Immunol..

[5] Doulatov S, Notta F, Laurenti E, Dick JE (2012). Hematopoiesis: a human perspective. Cell. Stem. Cell..

[6] Biasco L (2015). In vivo tracking of human hematopoiesis reveals patterns of clonal dynamics during early and steady-state reconstitution phases. Cell Stem Cell.

[7] Haas S, Trumpp A, Milsom MD (2018). Causes and consequences of hematopoietic stem cell heterogeneity. Cell Stem Cell.

[8] Pei W (2017). Polylox barcoding reveals haematopoietic stem cell fates realized in vivo. Nature.

[9] Notta F., Zandi S., Takayama N., Dobson S., Gan O. I., Wilson G., Kaufmann K. B., McLeod J., Laurenti E., Dunant C. F., McPherson J. D., Stein L. D., Dror Y., Dick J. E. (2015). Distinct routes of lineage development reshape the human blood hierarchy across ontogeny. Science.

[10] Velten L (2017). Human haematopoietic stem cell lineage commitment is a continuous process. Nat. Cell Biol..

[11] Zheng S, Papalexi E, Butler A, Stephenson W, Satija R (2018). Molecular transitions in early progenitors during human cord blood hematopoiesis. Mol. Syst. Biol..

[12] Hay SB, Ferchen K, Chetal K, Grimes HL, Salomonis N (2018). The Human Cell Atlas bone marrow single-cell interactive web portal. Exp. Hematol..

[13] Buenrostro JD (2018). Integrated single-cell analysis maps the continuous regulatory landscape of human hematopoietic differentiation. Cell.

[14] Sanjuan-Pla A (2013). Platelet-biased stem cells reside at the apex of the haematopoietic stem-cell hierarchy. Nature.

[15] Carrelha J (2018). Hierarchically related lineage-restricted fates of multipotent haematopoietic stem cells. Nature.

[16] Karamitros D (2018). Single-cell analysis reveals the continuum of human lympho-myeloid progenitor cells article. Nat. Immunol..

[17] Goardon N (2011). Coexistence of LMPP-like and GMP-like leukemia stem cells in acute myeloid leukemia. Cancer Cell..

[18] Civin CI (1984). Antigenic analysis of hematopoiesis. III. A hematopoietic progenitor cell surface antigen defined by a monoclonal antibody raised against KG-1a cells. J. Immunol..

[19] Psaila B (2016). Single-cell profiling of human megakaryocyte-erythroid progenitors identifies distinct megakaryocyte and erythroid differentiation pathways. Genome Biol..

[20] Basso-Ricci L (2017). Multiparametric whole blood dissection: a one-shot comprehensive picture of the human hematopoietic system. Cytom. Part A.

[21] De Jong MO, Wagemaker G, Wognum aW (1995). Separation of myeloid and erythroid progenitors based on expression of CD34 and c-kit. Blood.

[22] Zilionis R (2017). Single-cell barcoding and sequencing using droplet microfluidics. Nat. Protoc..

[23] Klein AM, Macosko E (2017). InDrops and Drop-seq technologies for single-cell sequencing. Lab. Chip..

[24] Tusi BK (2018). Population snapshots predict early haematopoietic and erythroid hierarchies. Nature.

[25] Weinreb, C., Wolock, S. & Klein, A. M. SPRING: a kinetic interface for visualizing high dimensional single-cell expression data. Bioinformatics **34**, 1246–1248 (2017). 10.1093/bioinformatics/btx792 PMC6030950 29228172

[26] Yáñez A (2017). Granulocyte-monocyte progenitors and monocyte-dendritic cell progenitors independently produce functionally distinct monocytes. Immunity.

[27] Haghverdi L, Buettner F, Theis FJ (2015). Diffusion maps for high-dimensional single-cell analysis of differentiation data. Bioinformatics.

[28] Görgens A (2013). Revision of the human hematopoietic tree: granulocyte subtypes derive from distinct hematopoietic lineages. Cell Rep..

[29] Drissen, R. et al. Europe PMC Funders Group Distinct myeloid progenitor differentiation pathways identified through single cell RNA sequencing. *Nat. Immunol.* **17**, 666–676 (2016). 10.1038/ni.3412 PMC4972405 27043410

[30] Mori Y (2009). Identification of the human eosinophil lineage-committed progenitor: revision of phenotypic definition of the human common myeloid progenitor. J. Exp. Med..

[31] Pishesha N (2014). Transcriptional divergence and conservation of human and mouse erythropoiesis. Proc. Natl. Acad. Sci..

[32] McGowan KA, Mason PJ (2011). Animal models of diamond Blackfan anemia. Semin. Hematol..

[33] Masiuk KE (2017). Improving gene therapy efficiency through the enrichment of human hematopoietic stem cells. Mol. Ther..

[34] Zonari E (2017). Efficient ex vivo engineering and expansion of highly purified human hematopoietic stem and progenitor cell populations for gene therapy. Stem Cell Rep..

[35] Watt SM (1998). Cd164, a novel sialomucin on cd34+ and erythroid subsets, is located on human chromosome 6q21. Blood.

[36] Watt SM (2000). Functionally defined CD164 epitopes are expressed on CD34(+) cells throughout ontogeny but display distinct distribution patterns in adult hematopoietic and nonhematopoietic tissues. Blood.

[37] Forde S (2007). Endolyn (CD164) modulates the CXCL12-mediated migration of umbilical cord blood CD133+ cells. Blood.

[38] Zannettino aC (1998). The sialomucin CD164 (MGC-24v) is an adhesive glycoprotein expressed by human hematopoietic progenitors and bone marrow stromal cells that serves as a potent negative regulator of hematopoiesis. Blood.

[39] Aiuti, A. et al. Lentiviral hematopoietic stem cell gene therapy in patients with wiskott-aldrich syndrome. Science **341**, 1233151 (2013). 10.1126/science.1233151 PMC4375961 23845947

[40] Notta F (2011). Isolation of single human hematopoietic stem cells capable of long-term multilineage engraftment. Science.

[41] Scala S (2018). Dynamics of genetically engineered hematopoietic stem and progenitor cells after autologous transplantation in humans. Nat. Med..

[42] Rodriguez-Fraticelli AE (2018). Clonal analysis of lineage fate in native haematopoiesis. Nature.

[43] Klein AM (2015). Droplet barcoding for single-cell transcriptomics applied to embryonic stem cells. Cell.

[44] Wu Shihao, Bertholet Peter, Huang Hui, Cohen-Or Daniel, Gong Minglun, Zwicker Matthias (2018). Structure-Aware Data Consolidation. IEEE Transactions on Pattern Analysis and Machine Intelligence.

[45] Team, R. Core. R.: a language and environment for statistical computing. R Foundation for Statistical Computing, Vienna, Austria. https://www.R-project.org/ (2013).

[46] Angerer Philipp, Haghverdi Laleh, Büttner Maren, Theis Fabian J., Marr Carsten, Buettner Florian (2015). destiny: diffusion maps for large-scale single-cell data in R. Bioinformatics.

[47] Trapnell Cole, Cacchiarelli Davide, Grimsby Jonna, Pokharel Prapti, Li Shuqiang, Morse Michael, Lennon Niall J, Livak Kenneth J, Mikkelsen Tarjei S, Rinn John L (2014). The dynamics and regulators of cell fate decisions are revealed by pseudotemporal ordering of single cells. Nature Biotechnology.

[48] Hastie, Trevor J. Generalized additive models. *Statistical models in S. Routledge* 249–307 (2017).

[49] Uhlen, Mathias et al. The human protein atlas. http://www.proteinatlas.Org (2015).

[50] Ravasi, Timothy et al. An atlas of combinatorial transcriptional regulation in mouse and man. *Cell* **140**, 744–752 (2010). 10.1016/j.cell.2010.01.044 PMC2836267 20211142

[51] Forbes Simon A., Beare David, Boutselakis Harry, Bamford Sally, Bindal Nidhi, Tate John, Cole Charlotte G., Ward Sari, Dawson Elisabeth, Ponting Laura, Stefancsik Raymund, Harsha Bhavana, Kok Chai Yin, Jia Mingming, Jubb Harry, Sondka Zbyslaw, Thompson Sam, De Tisham, Campbell Peter J. (2016). COSMIC: somatic cancer genetics at high-resolution. Nucleic Acids Research.

[52] Holm, S. A simple sequentially rejective multiple test procedure. *Scand. J. Stat.* **2**, 65–70 (1979).

[53] Yee, Thomas W. Vector generalized linear and additive models: with an implementation in R. XXIV, 589 (Springer-Verlag, New York, 2015).

[54] Smith Cynthia L, Blake Judith A, Kadin James A, Richardson Joel E, Bult Carol J (2017). Mouse Genome Database (MGD)-2018: knowledgebase for the laboratory mouse. Nucleic Acids Research.

[55] Croft David, Mundo Antonio Fabregat, Haw Robin, Milacic Marija, Weiser Joel, Wu Guanming, Caudy Michael, Garapati Phani, Gillespie Marc, Kamdar Maulik R., Jassal Bijay, Jupe Steven, Matthews Lisa, May Bruce, Palatnik Stanislav, Rothfels Karen, Shamovsky Veronica, Song Heeyeon, Williams Mark, Birney Ewan, Hermjakob Henning, Stein Lincoln, D'Eustachio Peter (2013). The Reactome pathway knowledgebase. Nucleic Acids Research.

[56] Weinreb Caleb, Wolock Samuel, Tusi Betsabeh K., Socolovsky Merav, Klein Allon M. (2018). Fundamental limits on dynamic inference from single-cell snapshots. Proceedings of the National Academy of Sciences.

