# Supplementary material for: A comprehensive single cell transcriptional landscape of human hematopoietic progenitors
Source: Nat Commun. 2019 Jun 3;10:2395. doi: 10.1038/s41467-019-10291-0 (PMC6546699; doi:10.1038/s41467-019-10291-0)
Supplement: Supplementary file 1 — Supplementary Information [file 41467_2019_10291_MOESM1_ESM.pdf]

Supplementary Information for manuscript:
"A comprehensive landscape of the single-cell transcriptome of human hematopoietic progenitors".

Pellin et al.

Supplementary Figures

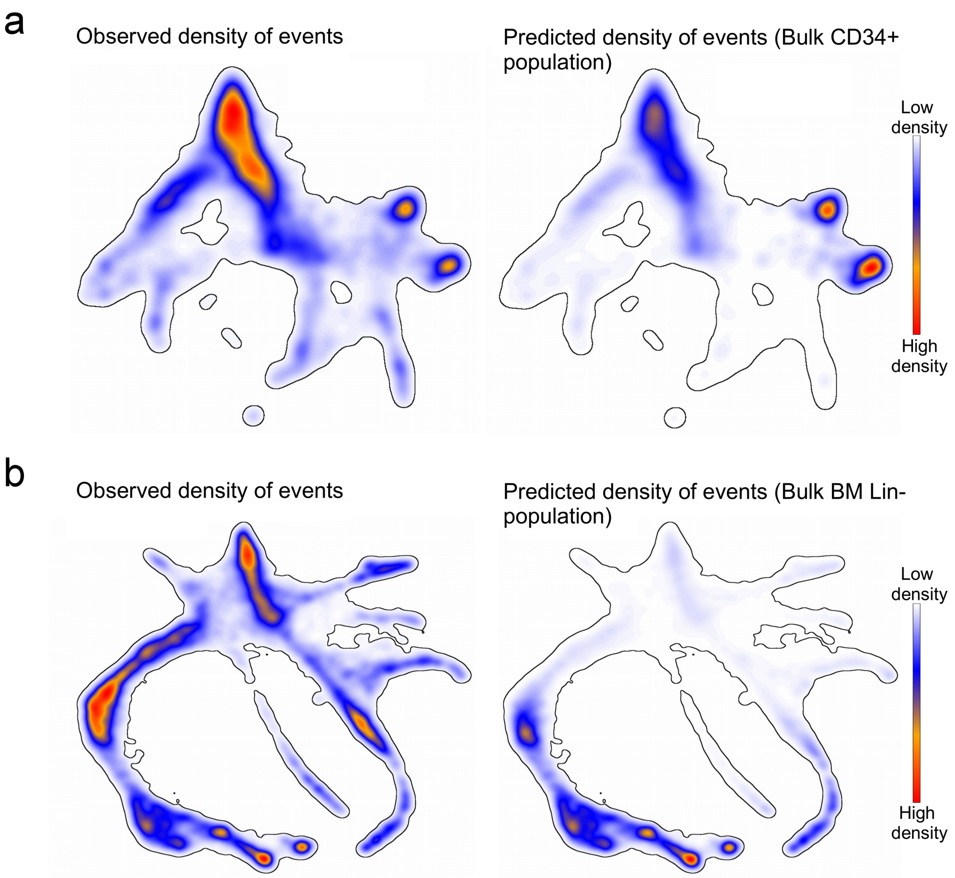

Supplementary Figure 1 | Observed and predicted cell density estimations by means of nonparametric kernel method. Observed (left) and predicted (right) cell density estimation for the transcriptome maps of a) sorted HSPCs and b) sorted Lin-CD34/CD164 cells. Details for the generation of this figure are provided in the Supplementary Table 2.

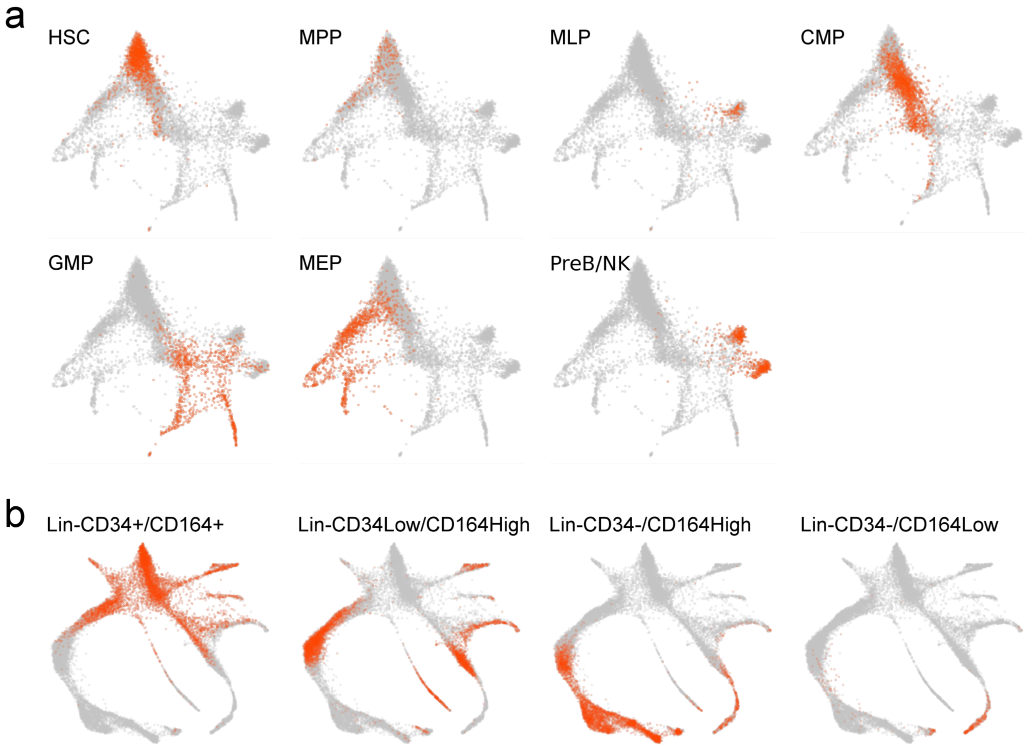

Supplementary Figure 2 | Visualization of the sorted subpopulations on the SPRING graphs. The FACS-sorted cell fractions have been individually highlighted in orange on the corresponding SPRING graphs. a) The seven sorted HSPC subpopulations. b) The four fractions isolated in Lin-CD34/CD164 cells.

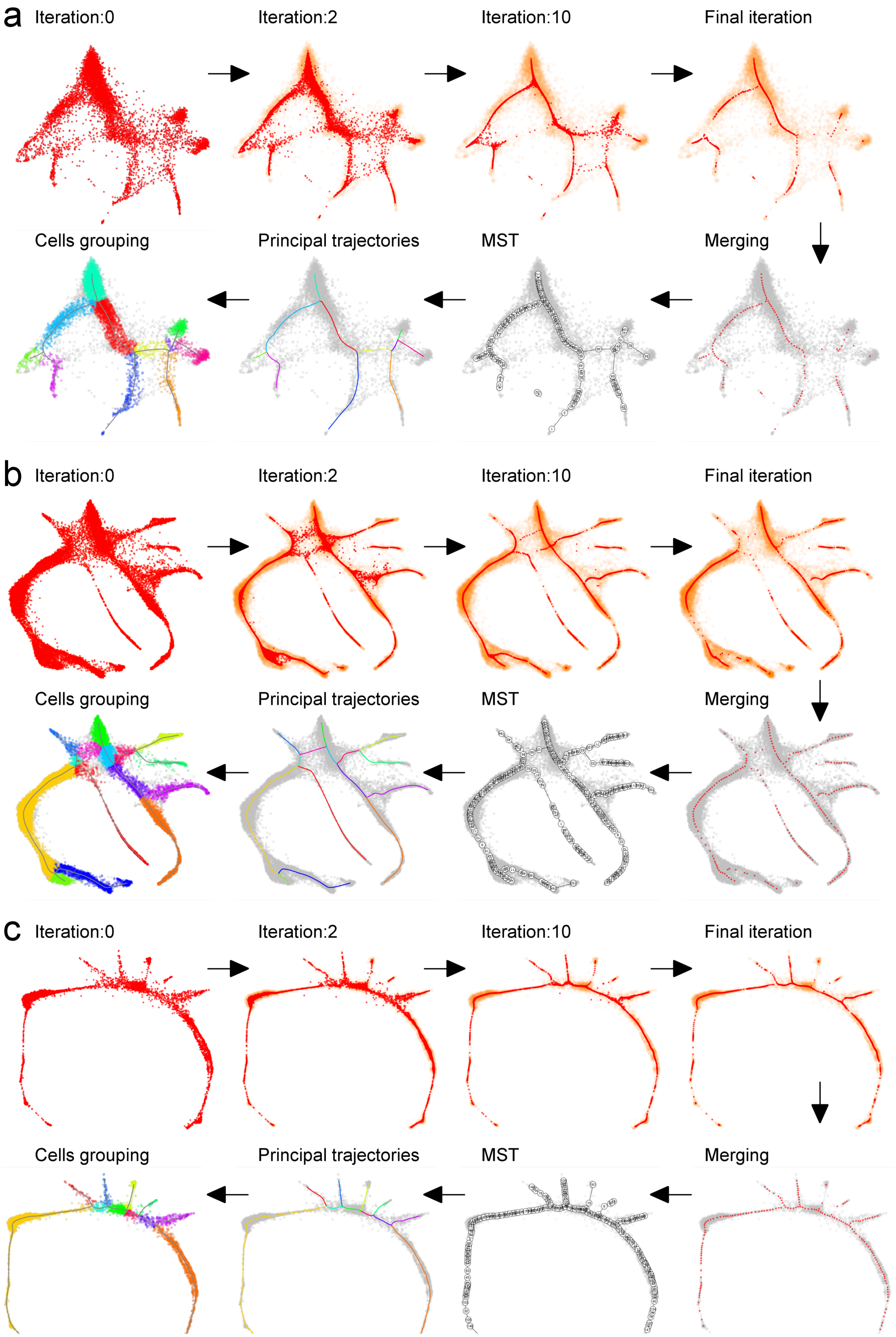

a
Iteration:0
Iteration:2
Iteration:10
Final iteration
Cells grouping
Principal trajectories
MST
Merging
b
Iteration:0
Iteration:2
Iteration:10
Final iteration
Cells grouping
Principal trajectories
MST
Merging
c
Iteration:0
Iteration:2
Iteration:10
Final iteration
Cells grouping
Principal trajectories
MST
Merging

Supplementary Figure 3 | Transcriptional principal trajectories identification procedure. Graphical representation of key intermediate steps underlying the estimation of principal trajectories for the transcriptomes of a) sorted HSPCs, b) sorted Lin-CD34/CD164 cells and c) mouse Kit+ cells. Graphs showing *Iteration:0*: consolidation points initialization; *Iteration:2:* and *Iteration:10*: consolidation points distribution after 2 and 10 iterations; *Final iteration*: estimated consolidation points distribution returned by structure-aware filtering algorithm; *Merging*: consolidation point set reduction by iterative merging; *MST*: branching reconstruction by Minimum Spanning Tree; *Principal trajectories*: segmentation of reconstructed skeleton; *Cells grouping*: cells-branch association.

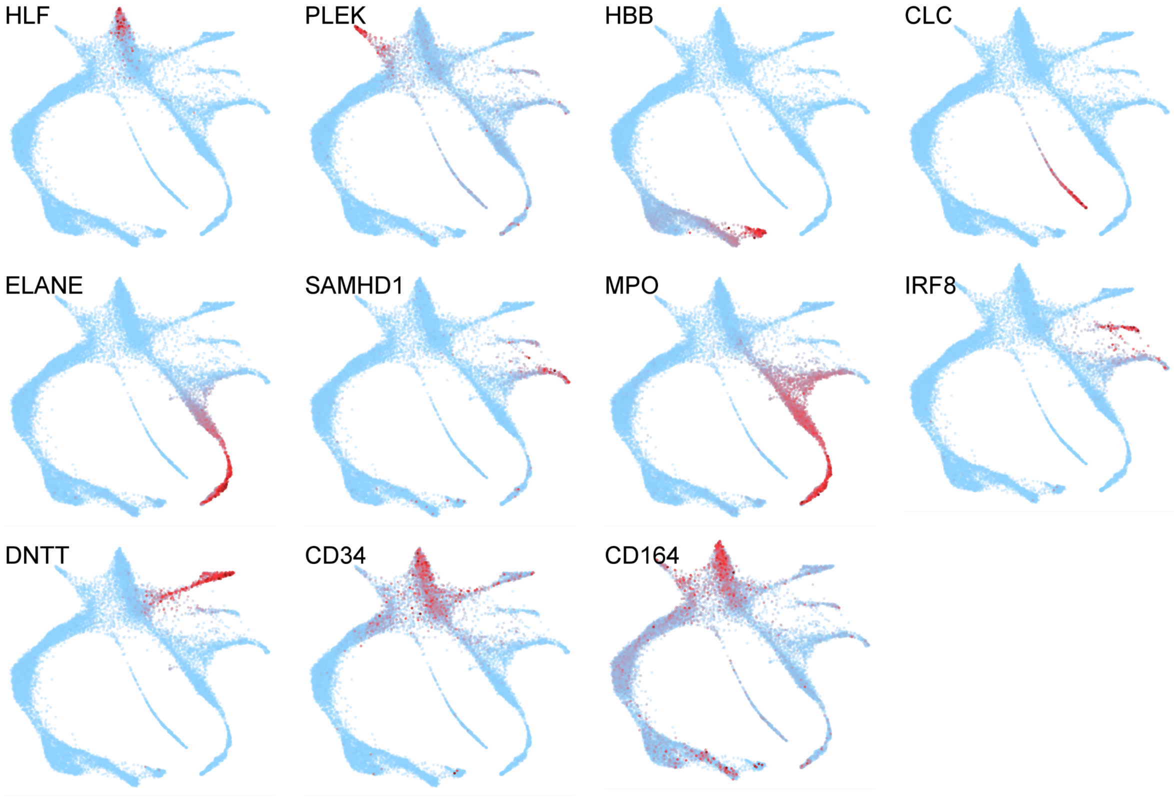

Supplementary Figure 4 | Gene expression maps for relevant genes. Lineage defining genes of the sorted Lin-CD34/CD164 cells: HLF, P; PLEK, Meg; HBB, E; CLC, BEMP; ELANE, N; SAMHD1, M; MPO, undifferentiated granulocytes; IRF8, DC; DNTT, Ly. Other genes: CD34; CD164.

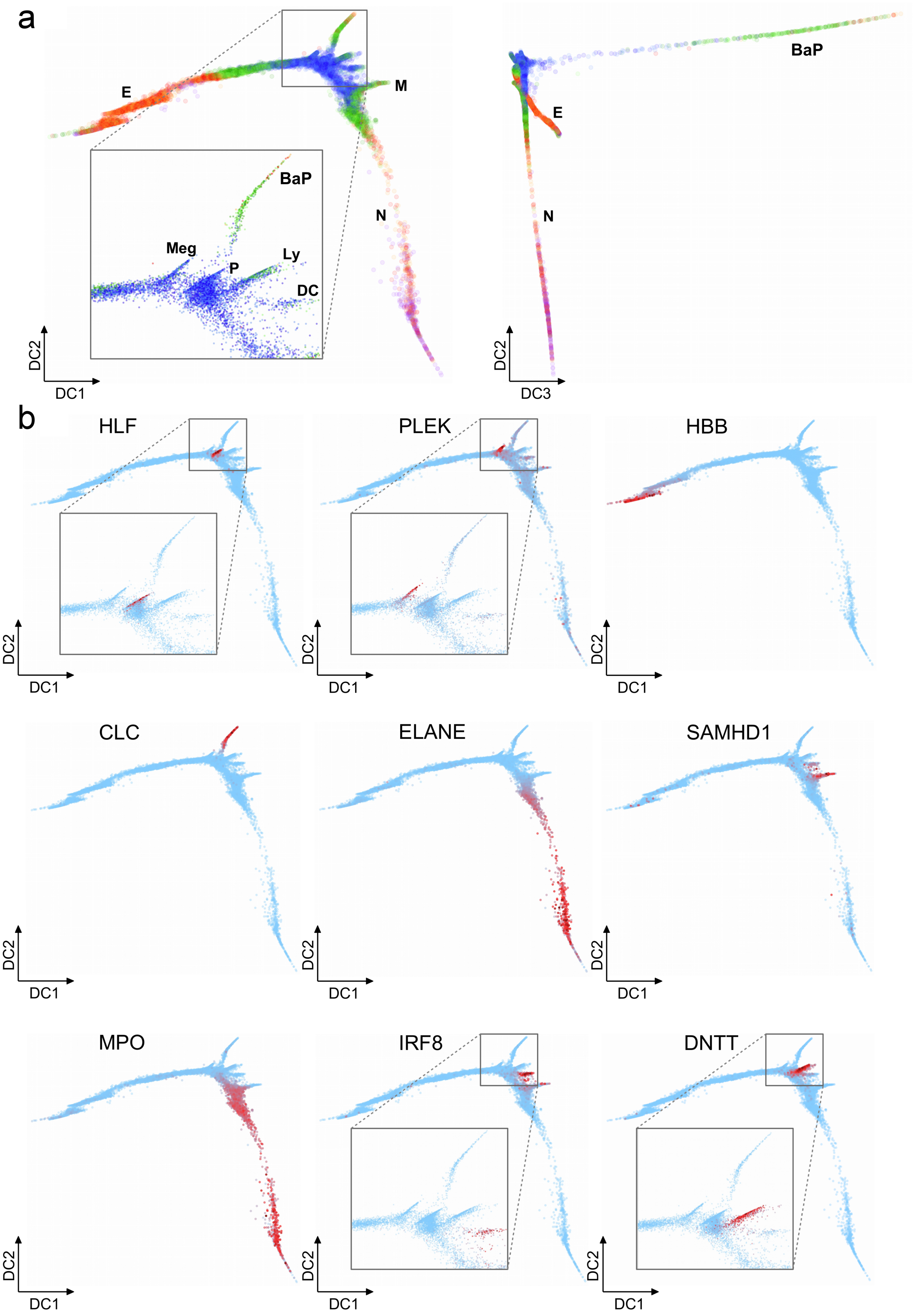

a
E
M
BaP
Meg
P
Ly
DC
N
DC2
DC1
BaP
E
N
DC2
DC3
b
HLF
PLEK
HBB
CLC
ELANE
SAMHD1
MPO
IRF8
DNTT
DC2
DC1

Supplementary Figure 5 | Diffusion map representation for Lin-CD34/CD164 dataset. a) Two views on the 3-dimensional diffusion map calculated. Diffusion map[1] tips have been labeled according to specific gene expression signatures reported in section **b)** and used also in Fig 2b and Supplementary Figure 4. Diffusion components 1 and 2 (DC1, DC2) capture lympho-myeloid and erythroid heterogeneity, whereas DC3 describes Baso-Eosinophil differentiation progression.

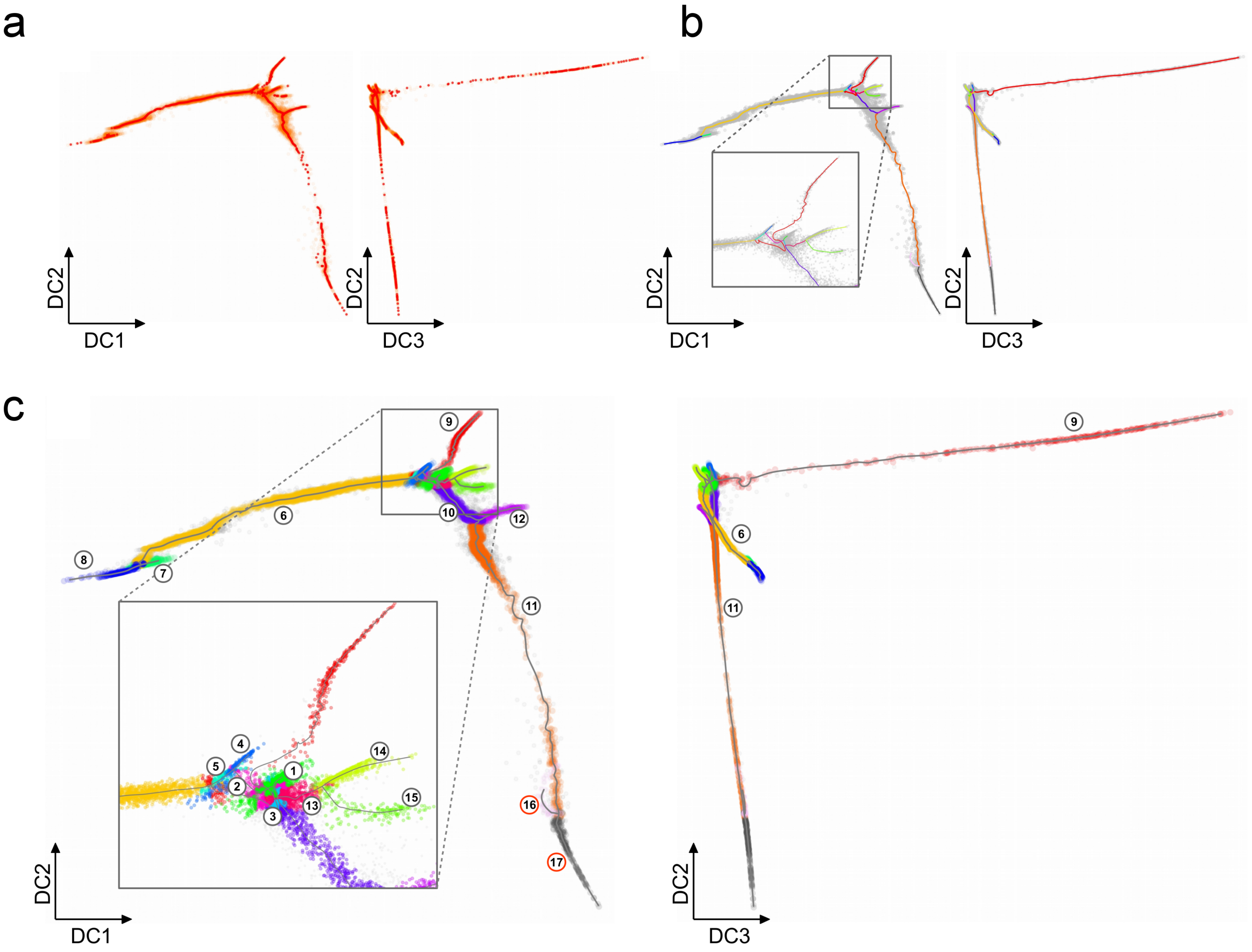

Supplementary Figure 6 | Transcriptional principal trajectories identification procedure on diffusion map for Lin-CD34/CD164 dataset. a) Estimated consolidation points distribution returned by structure-aware filtering algorithm; b) segmentation of reconstructed skeleton; c) cells-branch association. The procedure identifies 17 segments in total. Inferred skelethon and groups 1-15 closely recapitulate the results obtained starting from SPRING topology shown in Fig. 2d. Groups color code has been chosen according to Fig. 2d in order to facilitate the comparison.

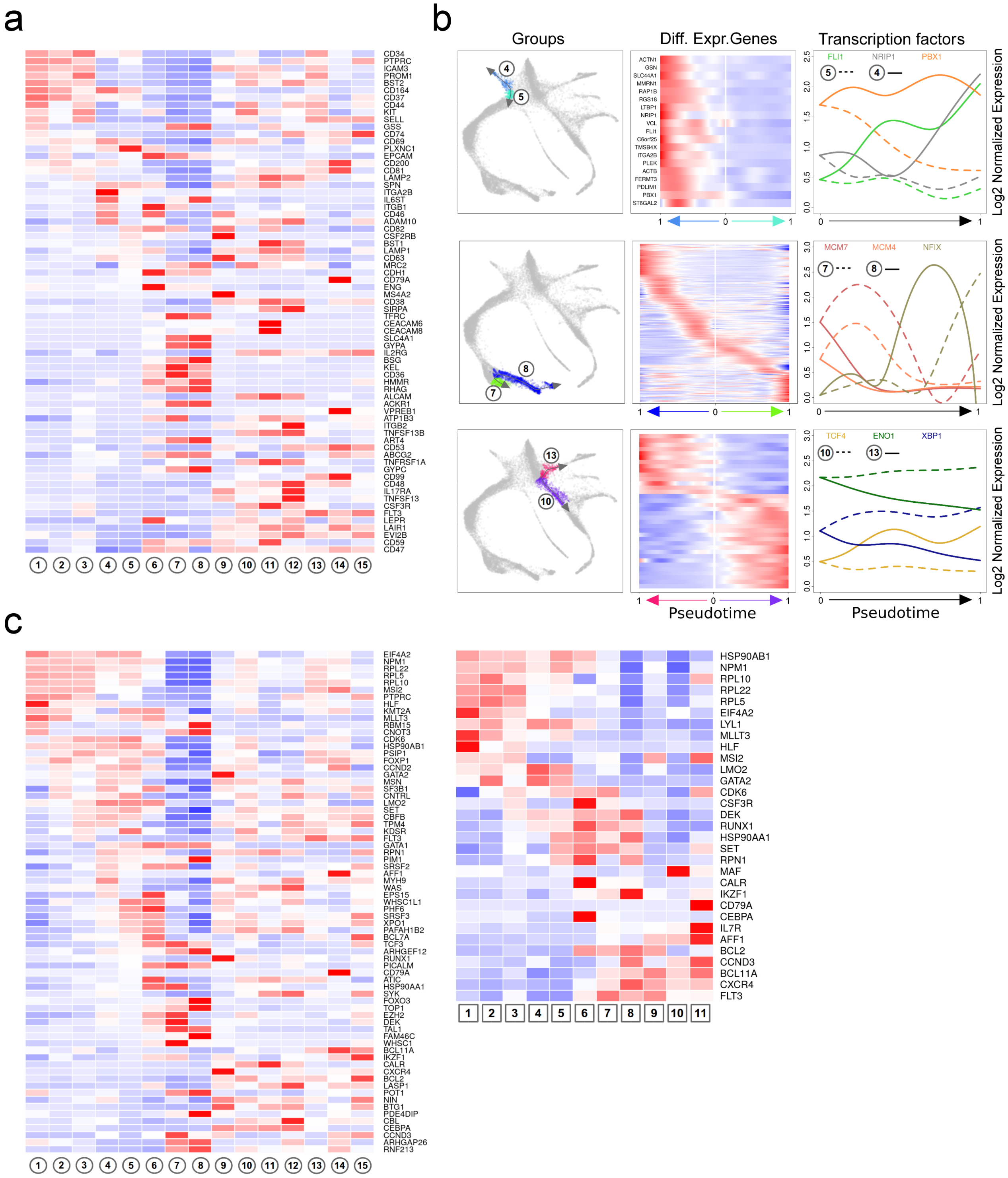

a
CD34
PTPRC
ICAM3
PROM1
BST2
CD164
CD37
CD44
KIT
SELL
GSS
CD74
CD69
PLXNC1
EPCAM
CD200
CD81
LAMP2
SPN
ITGA2B
IL6ST
ITGB1
CD46
ADAM10
CD82
CSF2RB
BST1
LAMP1
CD63
MRC2
CDH1
CD79A
ENG
MS4A2
CD38
SIRPA
TFRC
CEACAM6
CEACAM8
SLC4A1
GYPA
IL2RG
BSG
KEL
CD36
HMMR
RHAG
ALCAM
ACKR1
VPREB1
ATP1B3
ITGB2
TNFSF13B
ART4
CD53
ABCG2
TNFRSF1A
GYPC
CD99
CD48
IL17RA
TNFSF13
CSF3R
FLT3
LEPR
LAIR1
EVI2B
CD59
CD47
1 2 3 4 5 6 7 8 9 10 11 12 13 14 15
b
Groups
Diff. Expr.Genes
Transcription factors
ACTN1
GSN
SLC44A1
MMRN1
RAP1B
RGS18
LTBP1
NRIP1
VCL
FLI1
C6orf25
TMSB4X
ITGA2B
PLEK
ACTB
FERMT3
PDLIM1
PBX1
ST6GAL2
FLI1
NRIP1
PBX1
5
4
7
8
MCM7
MCM4
NFIX
13
10
TCF4
ENO1
XBP1
Log2 Normalized Expression
Pseudotime
c
EIF4A2
NPM1
RPL22
RPL5
RPL10
MSI2
PTPRC
HLF
KMT2A
MLLT3
RBM15
CNOT3
CDK6
HSP90AB1
PSIP1
FOXP1
CCND2
GATA2
MSN
SF3B1
CNTRL
LMO2
SET
CBFB
TPM4
KDSR
FLT3
GATA1
RPN1
PIM1
SRSF2
AFF1
MYH9
WAS
EPS15
WHSC1L1
PHF6
SRSF3
XPO1
PAFAH1B2
BCL7A
TCF3
ARHGEF12
RUNX1
PICALM
CD79A
ATIC
HSP90AA1
SYK
FOXO3
TOP1
EZH2
DEK
TAL1
FAM46C
WHSC1
BCL11A
IKZF1
CALR
CXCR4
BCL2
LASP1
POT1
NIN
BTG1
PDE4DIP
CBL
CEBPA
CCND3
ARHGAP26
RNF213
1 2 3 4 5 6 7 8 9 10 11 12 13 14 15
HSP90AB1
NPM1
RPL10
RPL22
RPL5
EIF4A2
LYL1
MLLT3
HLF
MSI2
LMO2
GATA2
CDK6
CSF3R
DEK
RUNX1
HSP90AA1
SET
RPN1
MAF
CALR
IKZF1
CD79A
CEBPA
IL7R
AFF1
BCL2
CCND3
BCL11A
CXCR4
FLT3
1 2 3 4 5 6 7 8 9 10 11

Supplementary Figure 7 | Gene expression variation among branches and fate decision signatures in Lin-CD34/CD164 transcriptome. a) Heatmap representation of gene expression levels among cells groups shown in Fig 2d for significant genes (LRT adjusted p-value <0.05, more details in Supplementary Information) known to code for CD markers[2]. Individual gene expression data have been row normalized among groups during heatmap generation. b) Gene dynamics associated to branching and fate decisions. The following comparisons are shown: 4 vs 5, 7 vs 8 and 10 vs 13. Columns report respectively: branching and groups considered (left); heatmaps of expression regression curves for genes showing a statistically significant difference (central); three significant transcription factors (right). c) Heatmaps for significant proto-oncogenes (LRT adjusted p-value <0.05, more details in Supplementary Information) with documented activity relevant to blood cancer according to COSMIC catalogue[3] among groups identified in sorted Lin-CD34/CD164 cells (left) and sorted HSPCs (right).

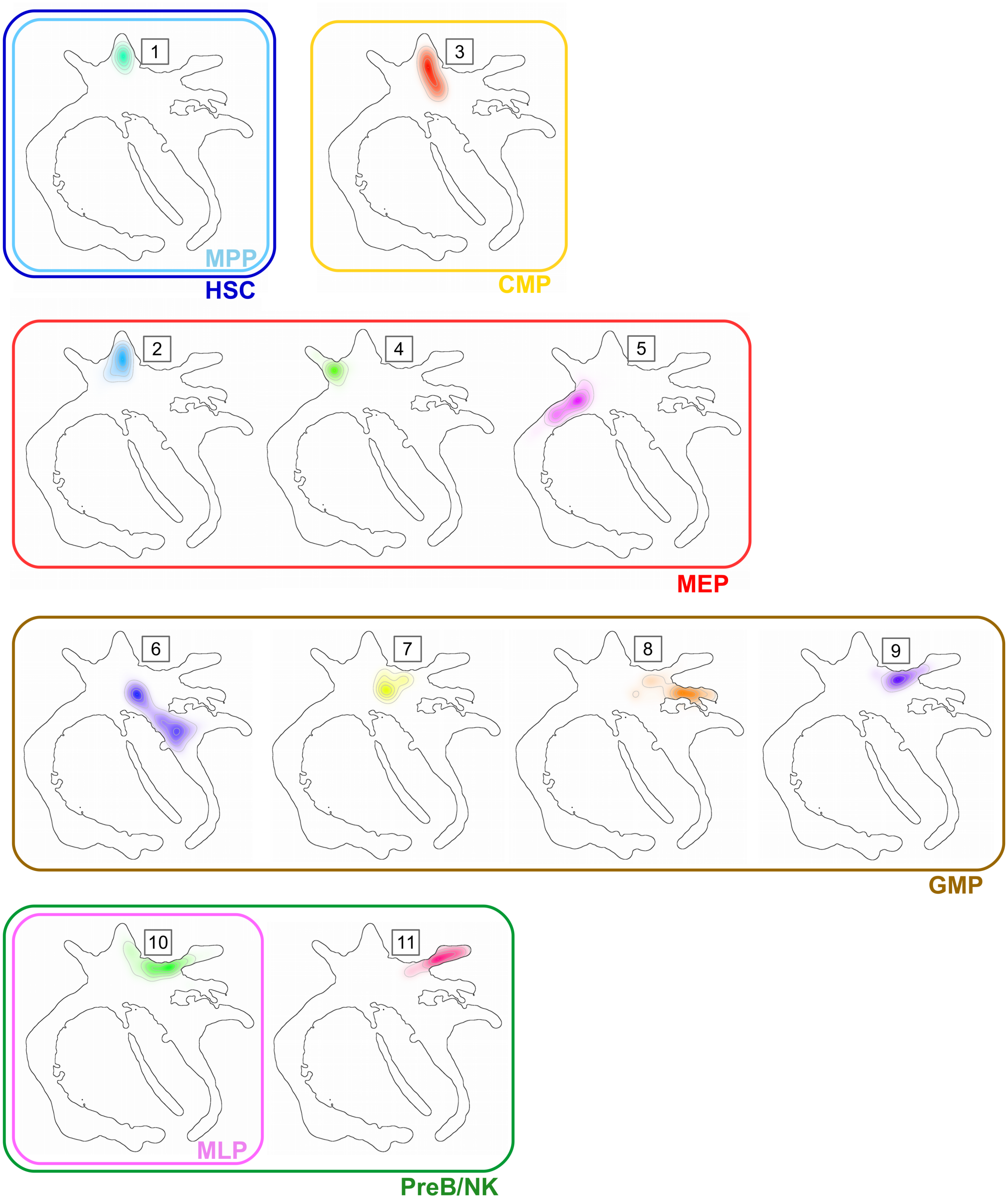

1
MPP
HSC
3
CMP
2
4
5
MEP
6
7
8
9
GMP
10
MLP
11
PreB/NK

Supplementary Figure 8 | Projection of computationally identified groups from the HSPCs map onto the Lin-CD34/CD164 topology. Each cells group derived from principal trajectories identification analysis of the sorted Lin-HSPCs has been projected on Lin-CD34/CD164 topology. The labels of the seven FACS-sorted subpopulations have been added.

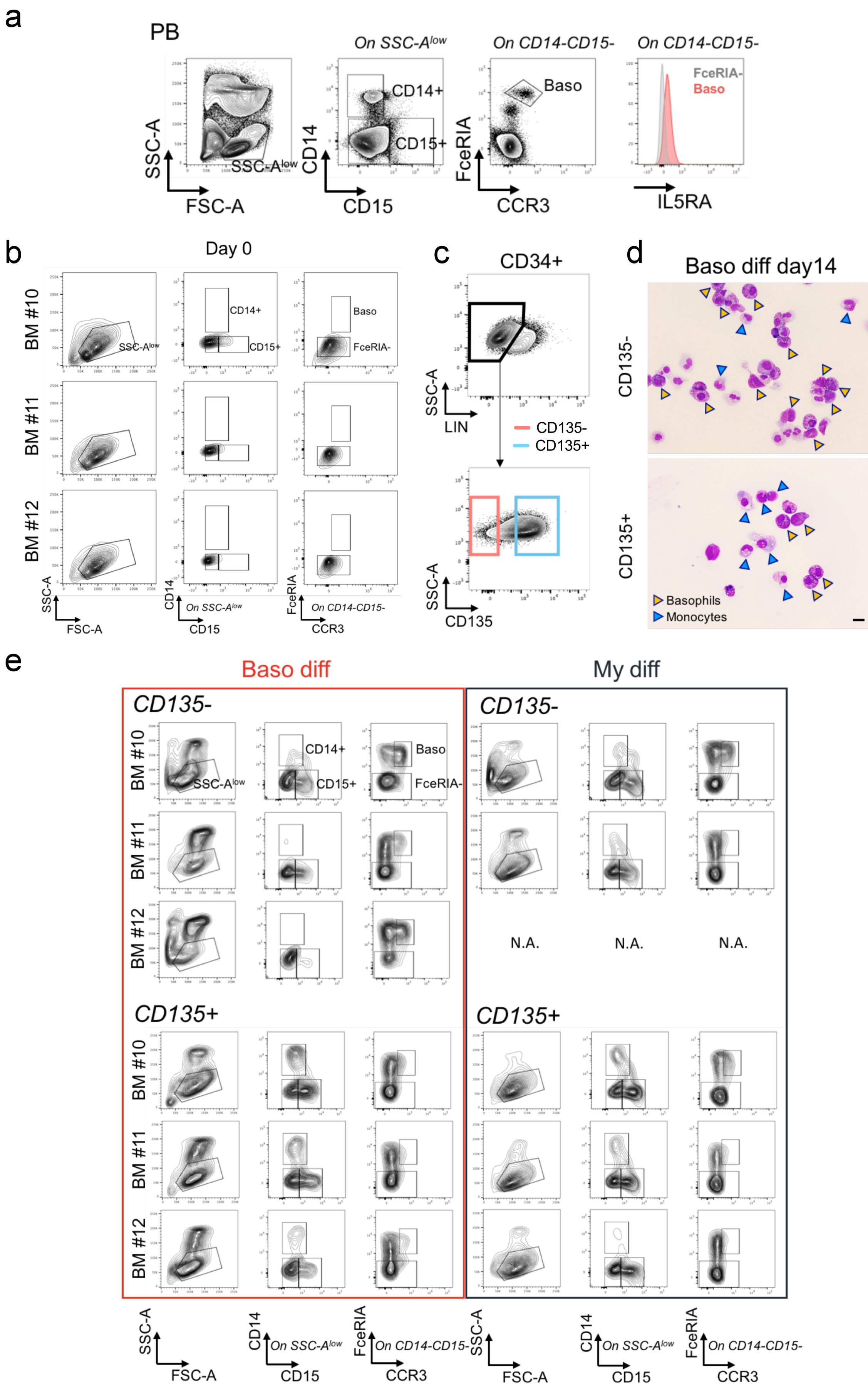
a
PB
On SSC-A^low
On CD14-CD15-
On CD14-CD15-
SSC-A
FSC-A
SSC-A^low
CD14
CD15
CD14+
CD15+
FceRIA
CCR3
Baso
FceRIA-
Baso
IL5RA
b
Day 0
BM #10
BM #11
BM #12
SSC-A^low
CD14+
CD15+
Baso
FceRIA-
SSC-A
FSC-A
CD14
On SSC-A^low
CD15
FceRIA
On CD14-CD15-
CCR3
c
CD34+
SSC-A
LIN
CD135-
CD135+
SSC-A
CD135
d
Baso diff day14
CD135-
CD135+
Basophils
Monocytes
e
Baso diff
My diff
CD135-
CD135-
BM #10
BM #11
BM #12
SSC-A^low
CD14+
CD15+
Baso
FceRIA-
N.A.
N.A.
N.A.
CD135+
CD135+
BM #10
BM #11
BM #12
SSC-A
FSC-A
CD14
On SSC-A^low
CD15
FceRIA
On CD14-CD15-
CCR3
SSC-A
FSC-A
CD14
On SSC-A^low
CD15
FceRIA
On CD14-CD15-
CCR3

Supplementary Figure 9 | Characterization of basophils in human peripheral blood and upon in vitro differentiation. a) Gating strategy developed for the identification and definition of basophils in the human peripheral blood. In the SSC-A low population, the fraction of CD14-CD15- cells was selected and investigated for the expression of FceRIA and CCR3. The double positive FceRIA+CCR3+ population has been defined as Basophils (Baso). This was confirmed by the IL-5RA expression in the Baso (red pick's shift) with respect to the control FceRIA- population (gray pick). b) Cytometric analyses of CD34+ cells from 3 independent BM using the gating strategy described in a). c) Giemsa staining of Lin-CD34+CD135- and Lin-CD34+CD135+ cells FACS-sorted from the 3 BM described in b) and cultured in basophil (Baso) differentiation culture. Basophils and Monocytes are indicated respectively with orange and blue arrow heads. Scale bar, 20µm. d) FACS plots showing the presence of basophils mostly in CD135- cells but CD135+ cells from the 3 BM described in b) upon differentiation in Baso differentiation culture (left panel). As control, the same cytometric analysis was performed upon My differentiation culture (right panel).

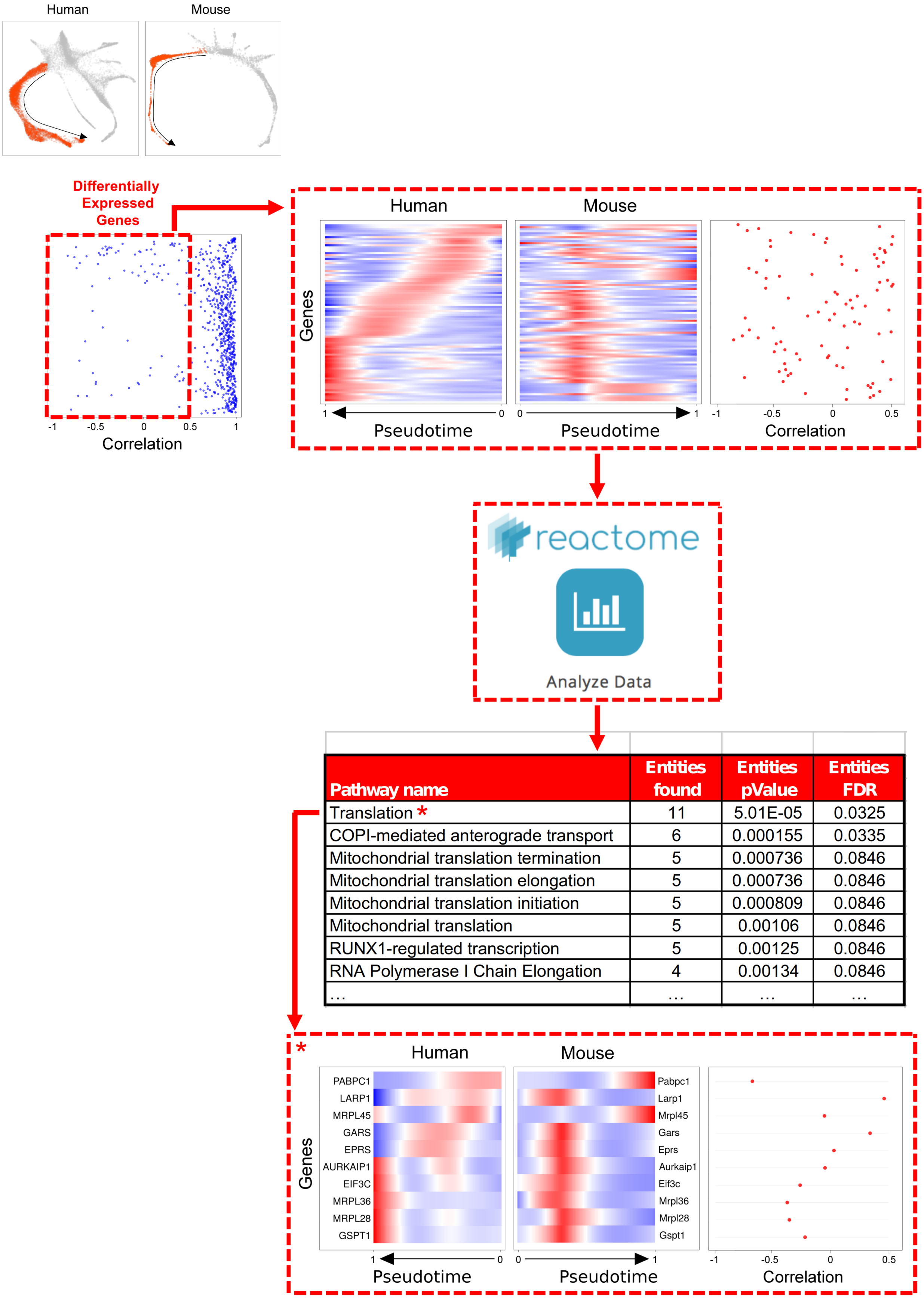

Pathway name	Entities found	Entities pValue	Entities FDR
Translation *	11	5.01E-05	0.0325
COPI-mediated anterograde transport	6	0.000155	0.0335
Mitochondrial translation termination	5	0.000736	0.0846
Mitochondrial translation elongation	5	0.000736	0.0846
Mitochondrial translation initiation	5	0.000809	0.0846
Mitochondrial translation	5	0.00106	0.0846
RUNX1-regulated transcription	5	0.00125	0.0846
RNA Polymerase I Chain Elongation	4	0.00134	0.0846
…	…	…	…

Supplementary Figure 10 | Focus on low-or-negative correlated genes among human and mouse erythropoiesis. Mirror heatmaps representing estimated regression curves for 89 orthologous genes exhibiting a low-or-negative correlation (Pearson correlation<0.5). Performing enrichment analysis by means of the Reactome pathway database tool[4], we found the *Translation* pathway as significantly over-represented (p-value: 5.01E-5). In the bottom dashed rectangle, a specific mirror heatmap for gene hits is shown.

a

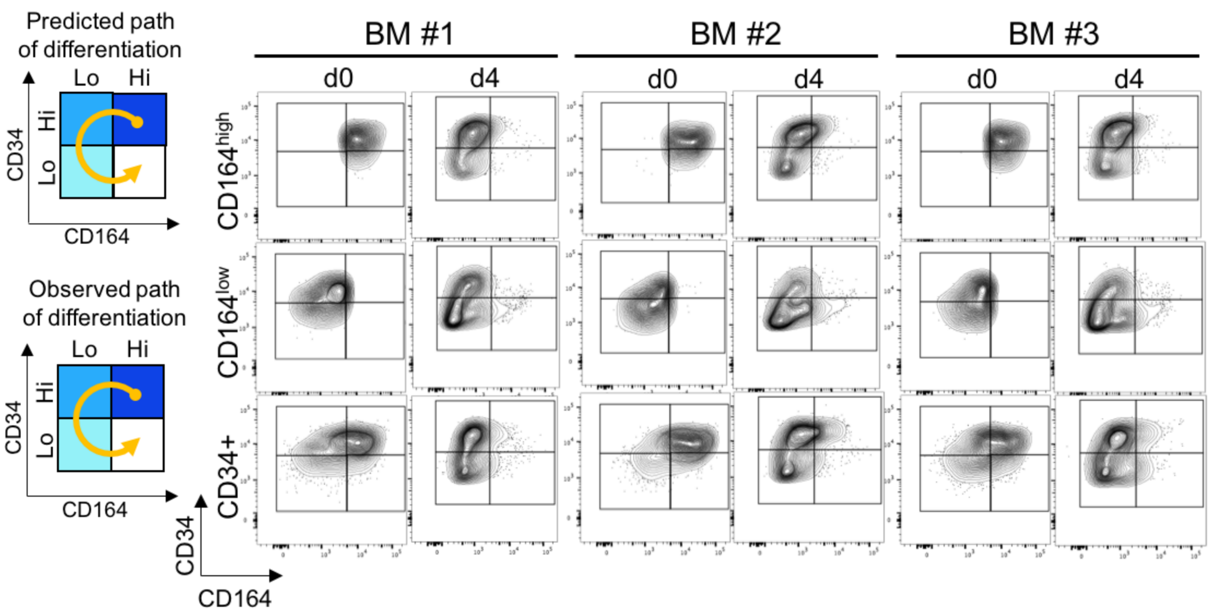

b

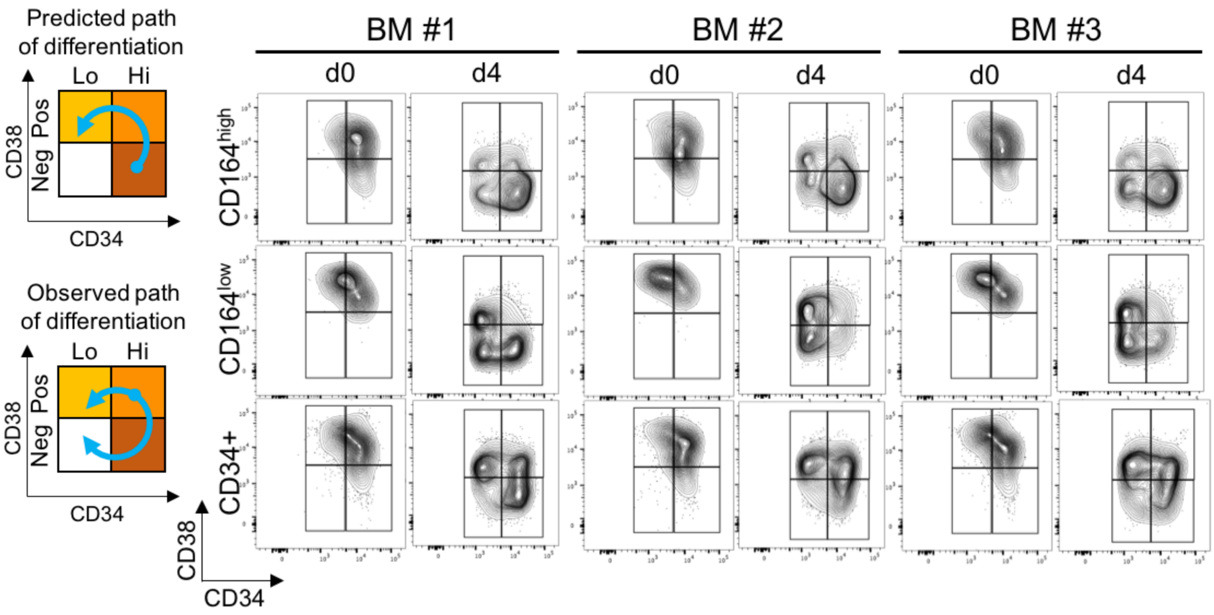

Supplementary Figure 11 | Cytometric analysis of CD38 and CD164 expression upon expansion culture. a) FACS plots showing the expression of CD164 with respect to CD34 and b) of CD38 with respect to CD34 in sorted CD34+CD164^{high} and CD34+CD164^{low} populations, at day 0 and day 4 in expansion culture. CD34+ cells were also analysed. Shown are 3 independent BM. Left schemes represent the predicted path of differentiation from the most primitive fractions of a) CD34^{high}CD164^{high} cells and b) CD34^{high}CD38^{neg} cells. Shown are also the schemes of the observed paths of differentiation.

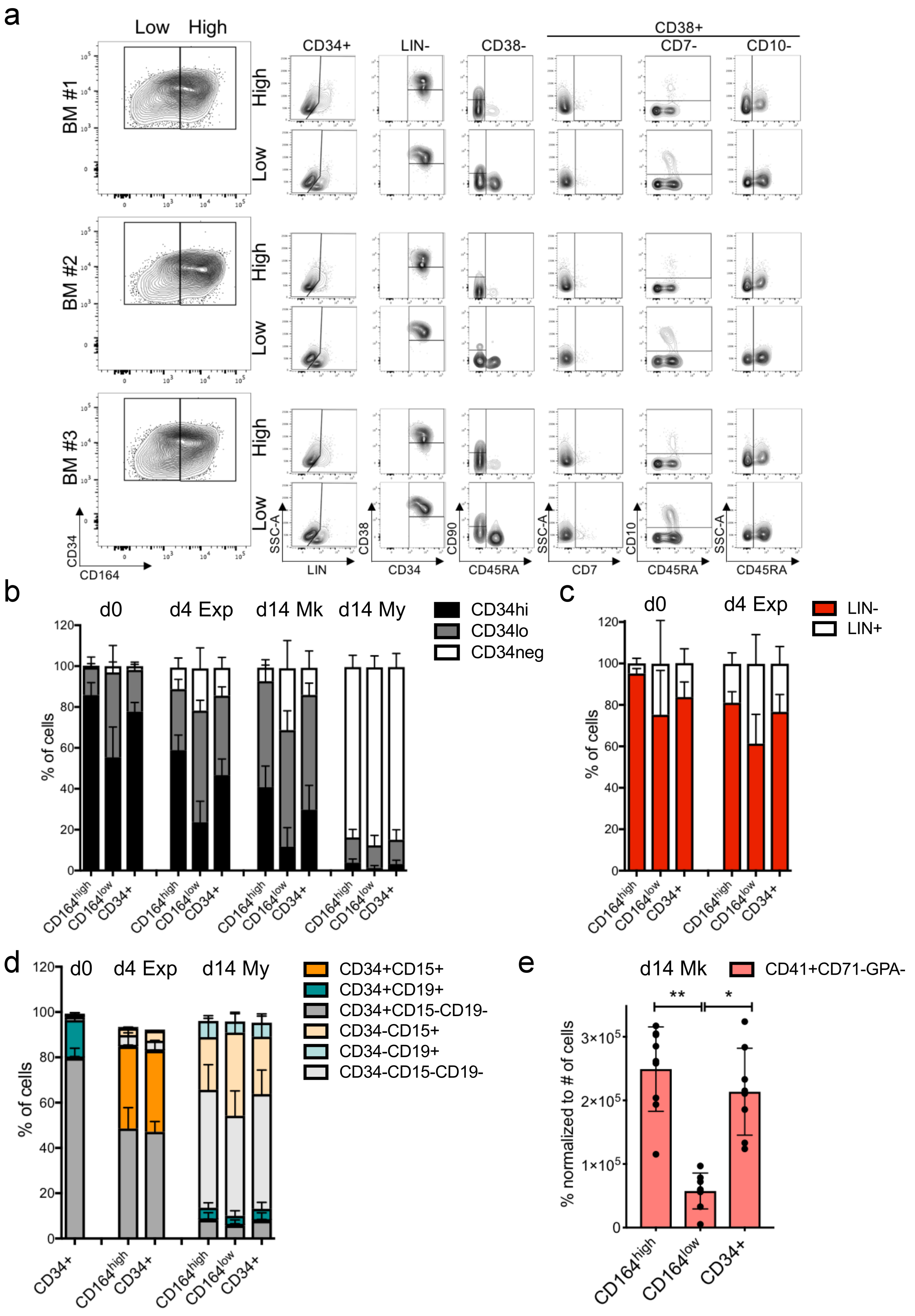
a
Low High
BM #1
BM #2
BM #3
CD34
CD164
CD34+
LIN-
CD38-
CD38+
CD7-
CD10-
High
Low
SSC-A
LIN
CD38
CD34
CD90
CD45RA
CD7
CD10
b
d0
d4 Exp
d14 Mk
d14 My
CD34hi
CD34lo
CD34neg
% of cells
c
LIN-
LIN+
d
CD34+CD15+
CD34+CD19+
CD34+CD15-CD19-
CD34-CD15+
CD34-CD19+
CD34-CD15-CD19-
e
d14 Mk
CD41+CD71-GPA-
% normalized to # of cells
CD164high
CD164low
CD34+

Supplementary Figure 12 | Additional data on immunophenotyping and in vitro functional assays. a) Immunophenotyping. FACS plots showing the content of Lin-/+ cells and HSPC subsets in the CD164high and CD164low fractions of CD34+ cells from 3 independent BM. b-e) In vitro assays on sorted CD164high and CD164low populations, and on CD34+ cells. b) Fractions of CD34high, CD34low and CD34neg at day 0 and in differentiation states at day 4 in Exp culture, or at day 14 in Mk and My culture conditions. Shown are Mean ± SD from 9 independent BM. c) Percentage of Lin- and Lin+ cells at day 0 and day 4 in Exp culture from 6 independent BM. Reported are Mean ± SD. d) Bar graph showing the expression of lineage positive markers CD15 and CD19 in CD34+ and CD34- cell fractions at day 0, at day 4 in Exp culture and day 14 in My culture from 9 independent BM. Shown are Mean ± SD. e) Percentage of CD41+CD71-GPA- cells normalized by the number of cells at the end of the megakaryocyte differentiation culture. Values are Mean ± SD from 9 independent BM. Statistics by Student's t-test (*$p<0.0005$, **$p<0.0001$).

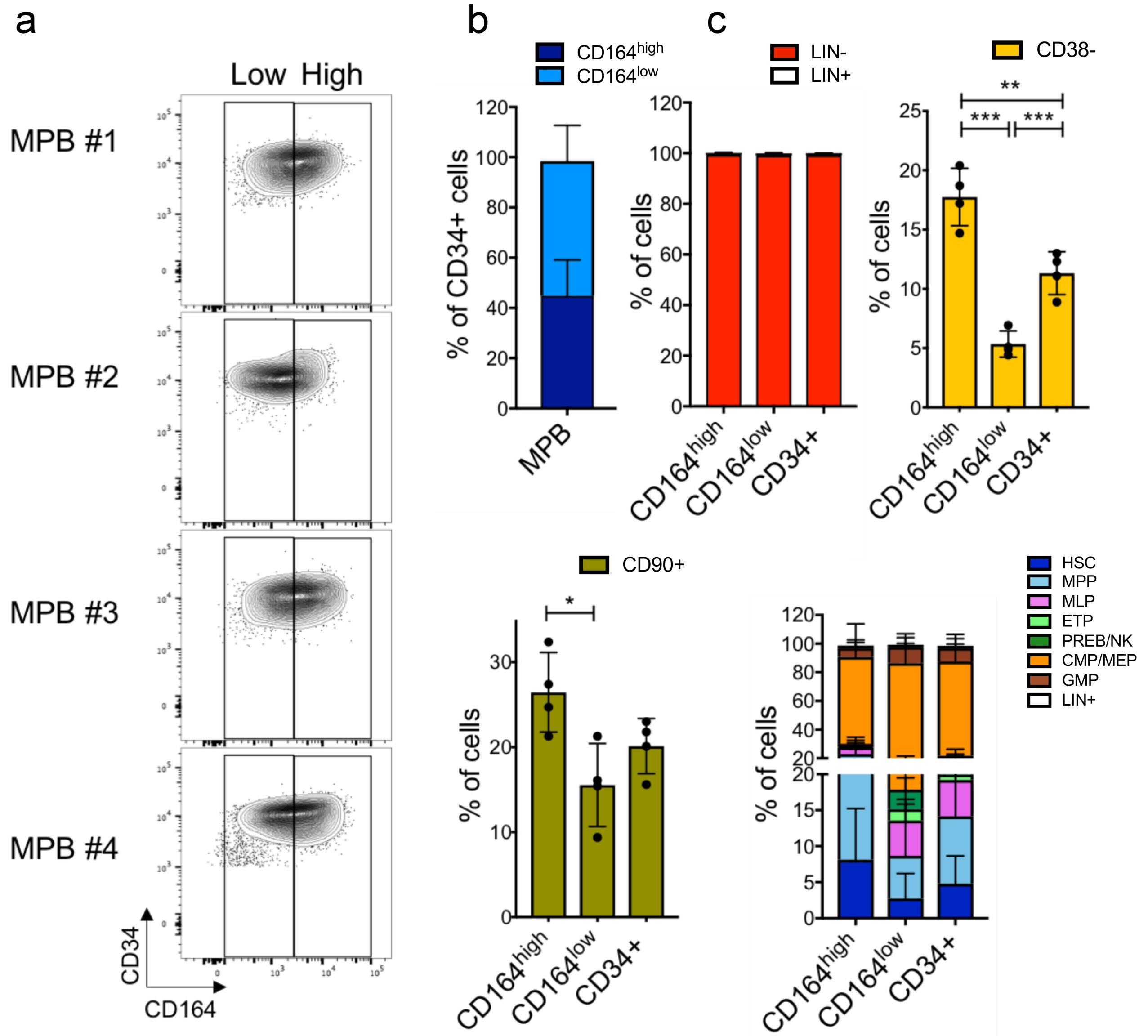

Supplementary Figure 13 | Immunophenotyping of G-CSF MPB CD34+ cells from 4 healthy donors. a) FACS plots showing $CD164^{high}$ and $CD164^{low}$ fractions in CD34+ cells from G-CSF MPB of 4 healthy donors. **b)** Bar graph showing the percentage of $CD164^{high}$ and $CD164^{low}$ fractions in MPB CD34+ cells. Values are Mean ± SD. **c)** Bar graphs showing the content respectively of Lin-/+, CD38-, CD90+ cells and HSPCs in $CD164^{high}$ and $CD164^{low}$ fractions, and in CD34+ cells. Values are Mean ± SD. Statistics by Student t-test (*p< 0.05; **p< 0.01; ***p< 0.005). For the HSPCs bar graph, statistics is provided in Supplementary Table 4.

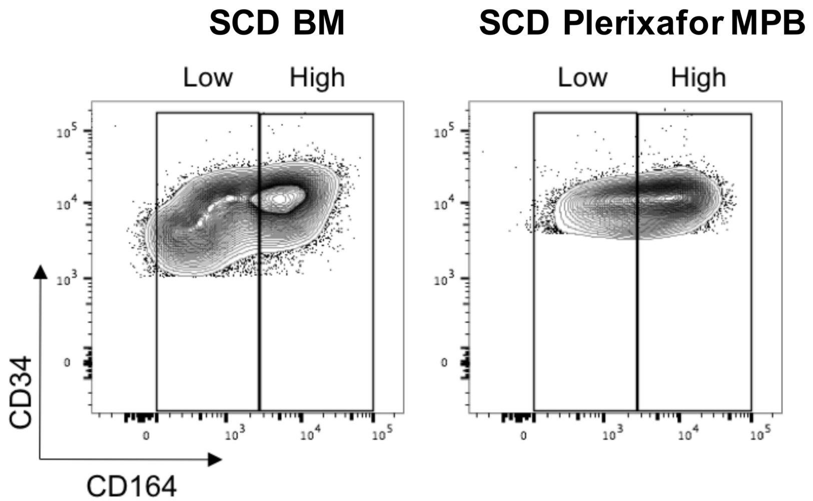

Supplementary Figure 14 | CD164 expression in a Sickle Cell Disease (SCD) patient. FACS plots showing CD164^{high} and CD164^{low} fractions in CD34+ cells from respectively BM (left) and plerixafor MPB (right) of the same SCD patient.

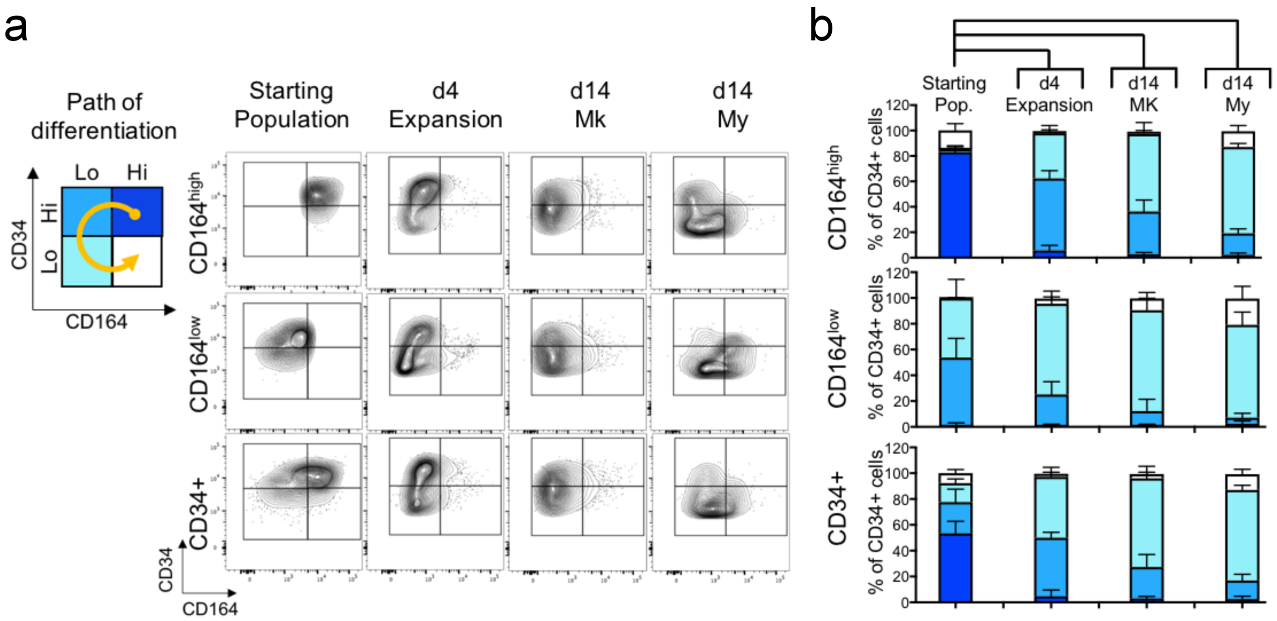

Supplementary Figure 15 | Cytometric analysis of CD164 expression in different culture conditions. a) FACS plots showing the cell phenotype at day 0 (Starting population) and in differentiation states at day 4 in Expansion culture, or at day 14 in Mk and My culture conditions. Top left, scheme representing the path of differentiation from the most primitive fraction of $CD34^{high}CD164^{high}$ cells. **b)** Bar graphs summarizing the cytometric analysis described in a). The colour code used is the same of the scheme in a). Shown are Mean ± SD from 9 independent BM.

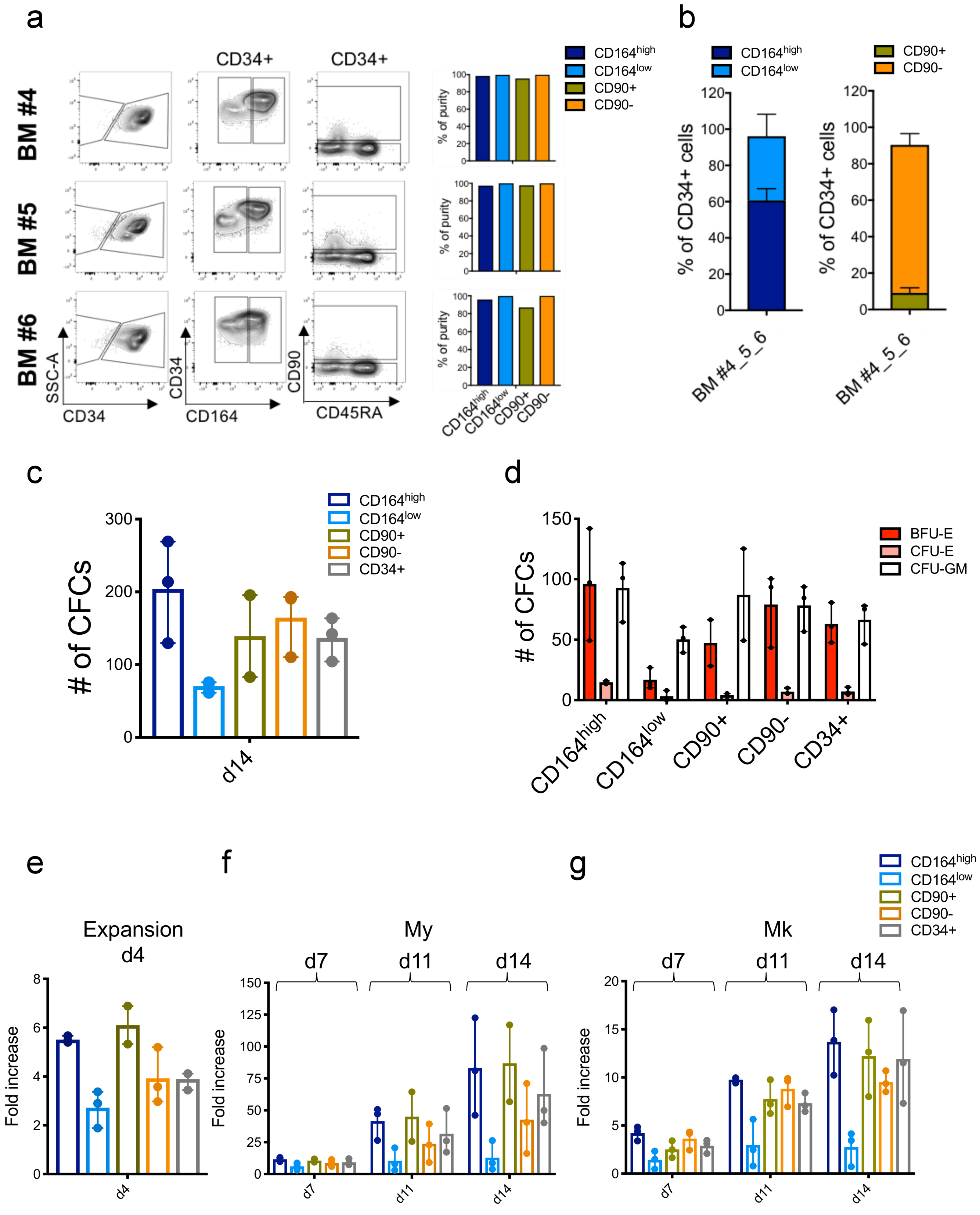
a
b
c
d
e
f
g
CD34+
CD34+
BM #4
BM #5
BM #6
SSC-A
CD34
CD164
CD90
CD45RA
% of purity
CD164high
CD164low
CD90+
CD90-
% of CD34+ cells
BM #4_5_6
# of CFCs
d14
CD34+
BFU-E
CFU-E
CFU-GM
Expansion
d4
My
Mk
d7
d11
d14
Fold increase

Supplementary Figure 16 | In vitro functional assays of CD164 versus CD90 subsets FACS-sorted from BM CD34+ cells of 3 additional healthy donors. a) Left: Gating strategy used to FACS-sort respectively CD164high/low fractions and CD90+/- fractions from BM CD34+ cells of 3 independent healthy donors. Right: Bar graphs showing the percentage of purity of each sorted population. **b)** Percentage of CD164high/low fractions (left) and CD90+/- fractions (right) in CD34+ cells. Shown are Median ± Error. **c)** Bar graphs showing the total number and **d)** the type of CFCs scored at day 14 in a colony-forming assay (CFCs, Colony Forming Cells, BFU-E, Burst-Forming Unit-Erythroid cells, CFU-E, Colony-Forming Unit-Erythroid cells, CFU-GM, Colony-Forming Unit-Granulocyte/Macrophages). Shown are Median ± Error. **e-g)** Bar graphs representing the growth rate of the sorted subsets and CD34+ cells in **e)** Expansion medium, **f)** My, myeloid differentiation medium and **g)** Mk, Megakaryocyte differentiation medium. Values are Median ± Error.

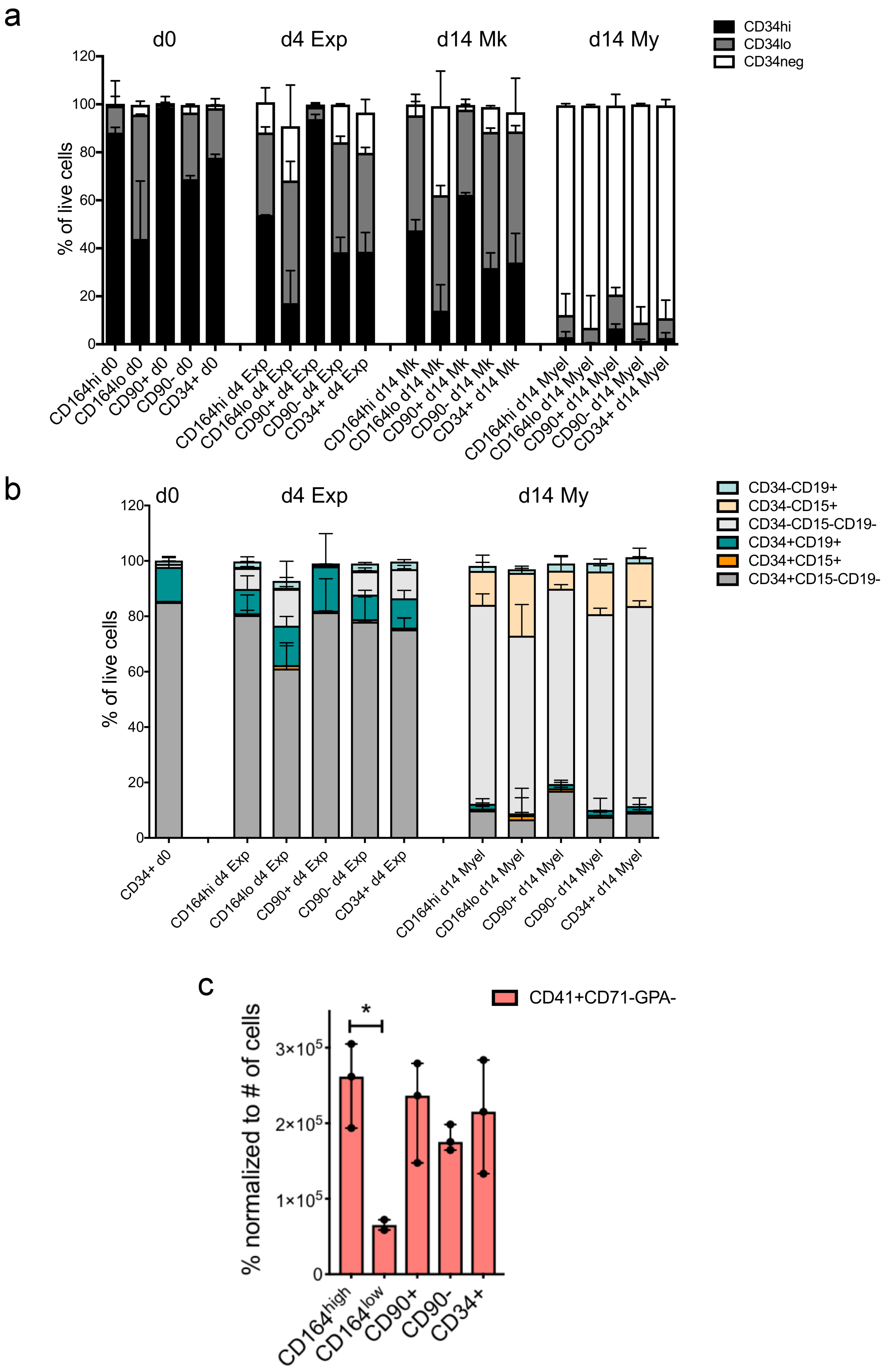
a
d0
d4 Exp
d14 Mk
d14 My
CD34hi
CD34lo
CD34neg
% of live cells
0
20
40
60
80
100
120
CD164hi d0
CD164lo d0
CD90+ d0
CD90- d0
CD34+ d0
CD164hi d4 Exp
CD164lo d4 Exp
CD90+ d4 Exp
CD90- d4 Exp
CD34+ d4 Exp
CD164hi d14 Mk
CD164lo d14 Mk
CD90+ d14 Mk
CD90- d14 Mk
CD34+ d14 Mk
CD164hi d14 Myel
CD164lo d14 Myel
CD90+ d14 Myel
CD90- d14 Myel
CD34+ d14 Myel
b
d0
d4 Exp
d14 My
CD34-CD19+
CD34-CD15+
CD34-CD15-CD19-
CD34+CD19+
CD34+CD15+
CD34+CD15-CD19-
% of live cells
0
20
40
60
80
100
120
CD34+ d0
CD164hi d4 Exp
CD164lo d4 Exp
CD90+ d4 Exp
CD90- d4 Exp
CD34+ d4 Exp
CD164hi d14 Myel
CD164lo d14 Myel
CD90+ d14 Myel
CD90- d14 Myel
CD34+ d14 Myel
c
CD41+CD71-GPA-
*
% normalized to # of cells
0
1×10^5
2×10^5
3×10^5
CD164^high
CD164^low
CD90+
CD90-
CD34+

Supplementary Figure 17 | Immunophenotypic profile of CD164 versus CD90 subsets upon in vitro functional assays of 3 additional healthy donors. **a)** Percentage of CD34high, CD34low and CD34neg at day 0 and in differentiation states at day 4 in Expansion (Exp) medium, or at day 14 in Megakaryocyte (Mk) and Myeloid (My) culture conditions of the sorted CD164high/low fractions and CD90+/- fractions, and CD34+ cells. **b)** Bar graph showing the expression of lineage positive markers CD15 and CD19 in CD34+ and CD34- cell fractions at day 0, at day 4 in Exp culture and day 14 in My culture. **c)** Percentage of CD41+CD71-GPA- cells normalized to the number of cells at the end of the megakaryocyte differentiation culture. Values are Median ± Error from 3 independent BM. Statistics by Student t-test (*$p < 0.05$).

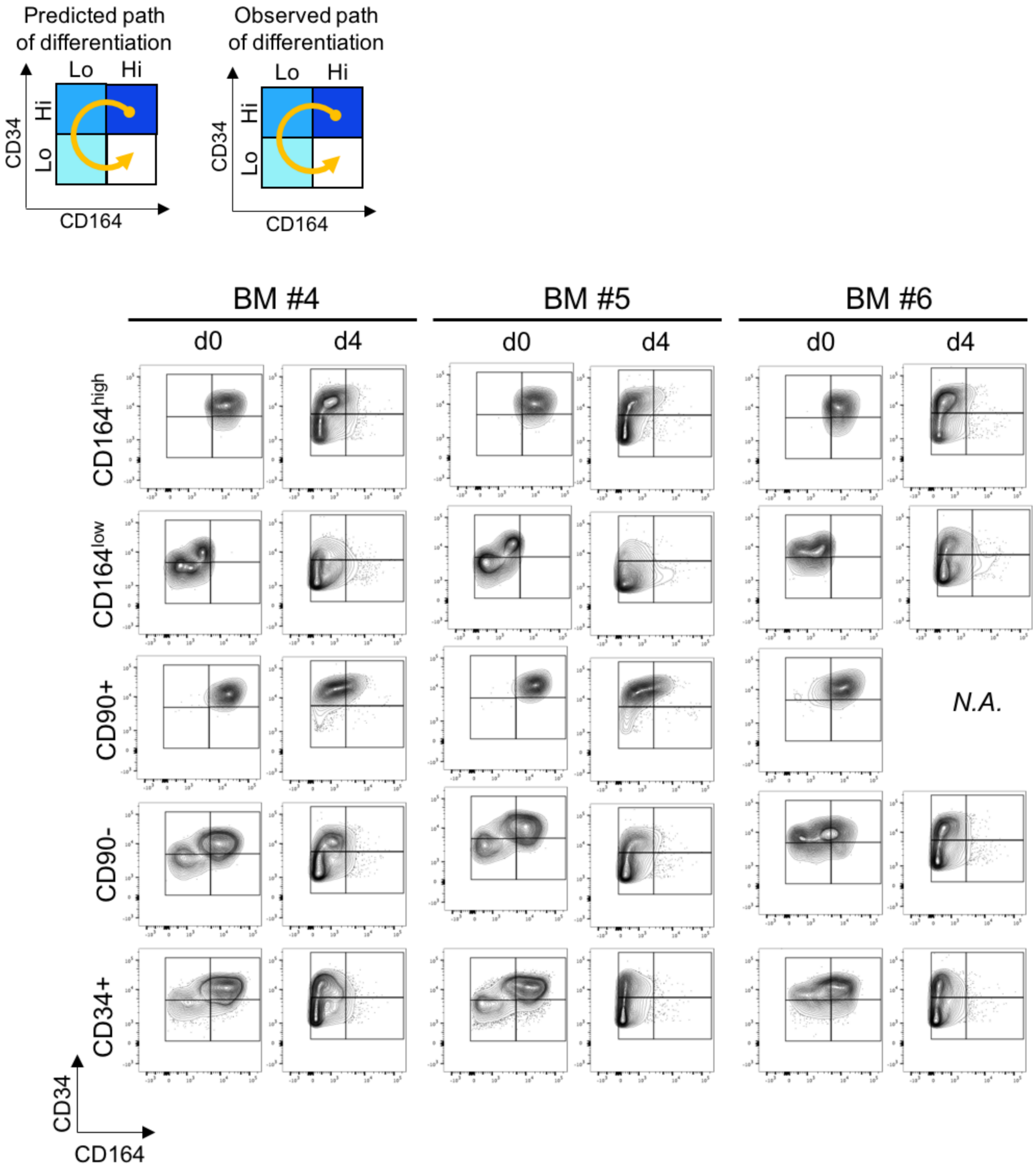

Supplementary Figure 18 | Cytometric analysis of CD164 and CD34 upon expansion culture. a) FACS plots showing the expression of CD164 with respect to CD34 in sorted CD164high/low fractions and CD90+/- fractions, and CD34+ cells at day 0 and day 4 in expansion culture. Shown are 3 independent BM. Left scheme represents the predicted path of differentiation from the most primitive fraction of CD34^{high}CD164^{high} cells. The observed path of differentiation (right scheme) perfectly overlaps.

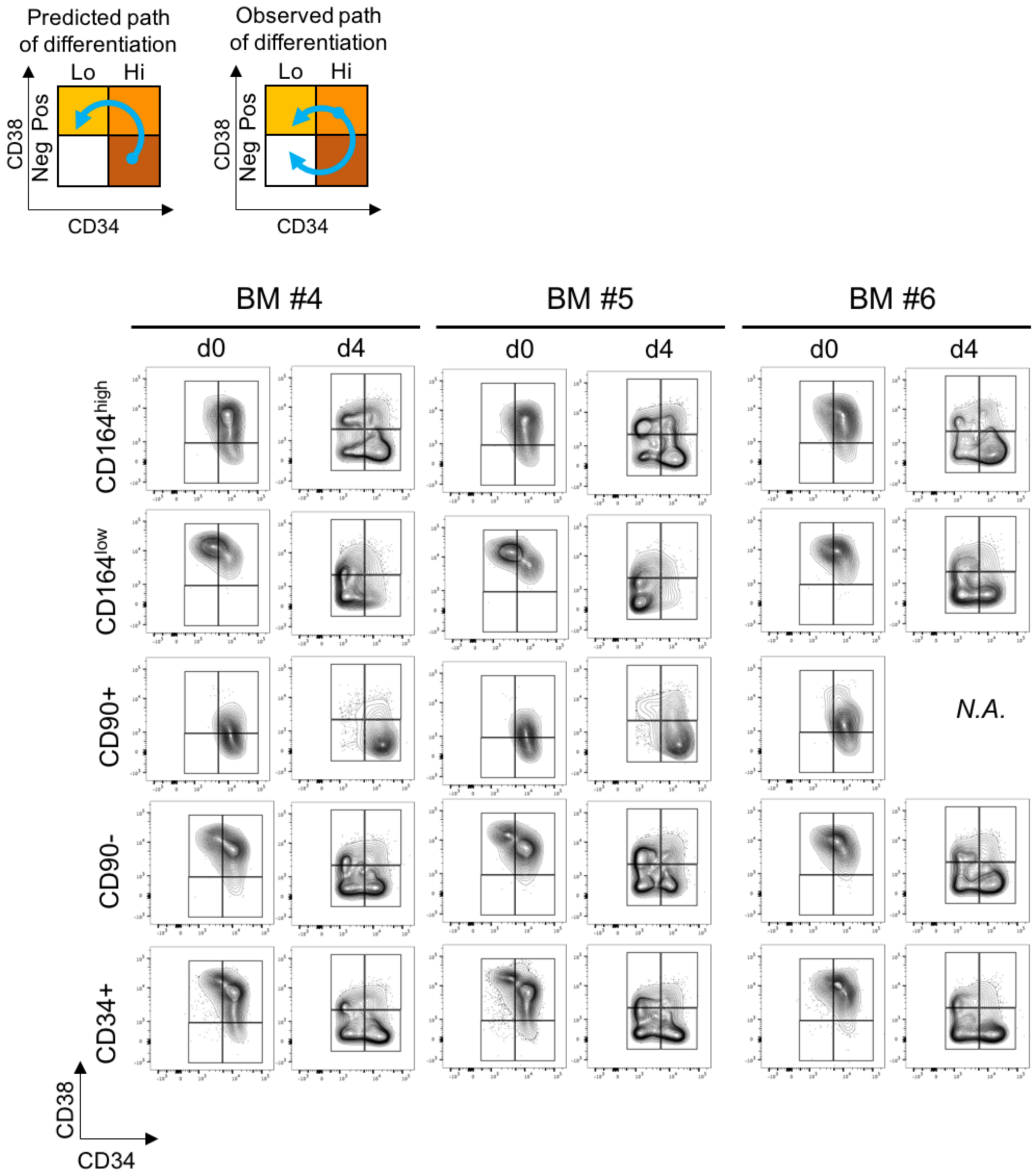

Supplementary Figure 19 | Cytometric analysis of CD38 and CD34 upon expansion culture. a) FACS plots showing the expression of CD38 with respect to CD34 in sorted CD164high/low fractions and CD90+/- fractions, and CD34+ cells at day 0 and day 4 in expansion culture. Shown are 3 independent BM. Left scheme represents the predicted path of differentiation from the most primitive fraction of CD34^{high}CD38^{neg} cells. The observed path of differentiation (right scheme) does not overlap.

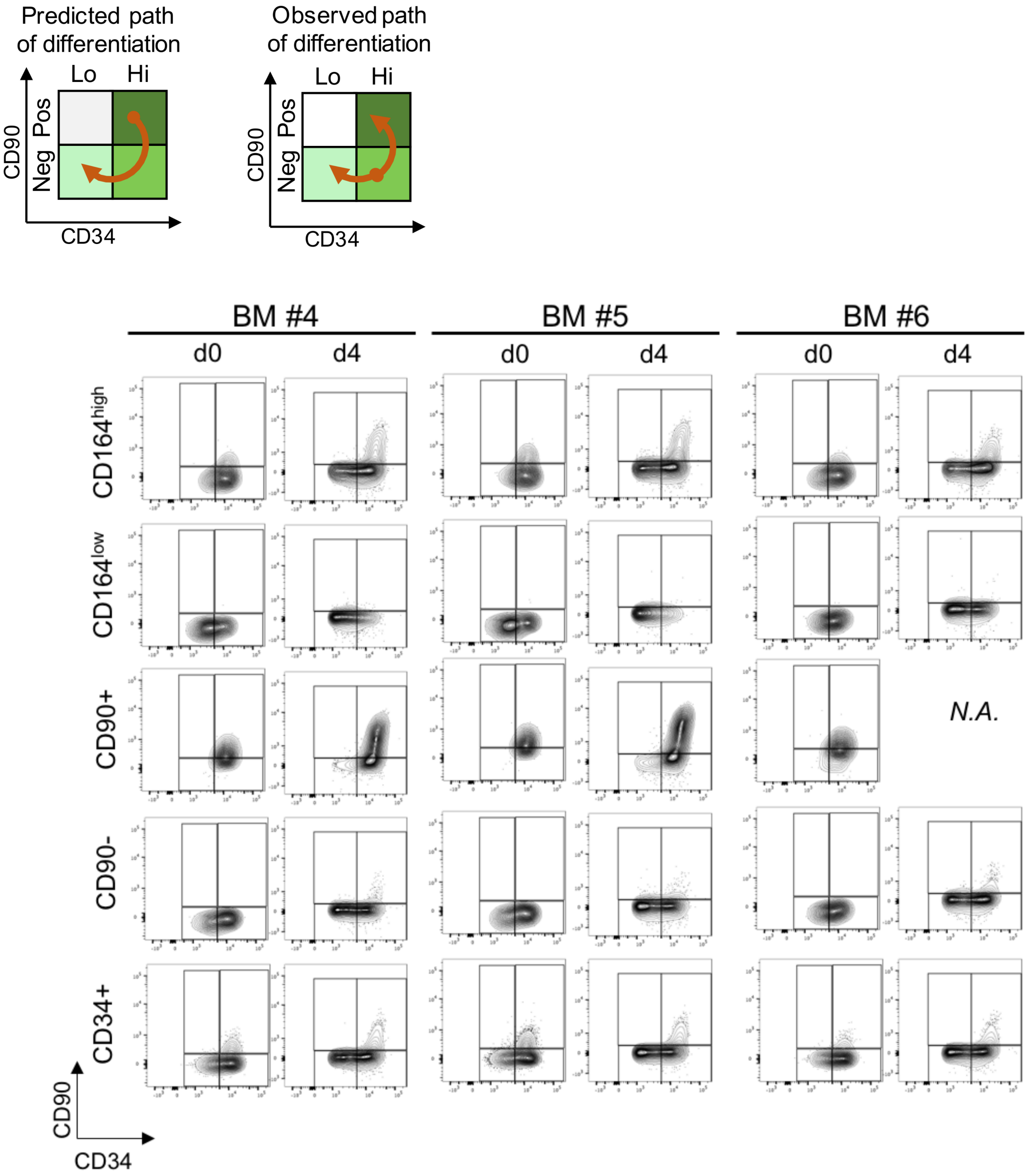

Supplementary Figure 20 | Cytometric analysis of CD90 and CD34 upon expansion culture. a) FACS plots showing the expression of CD90 with respect to CD34 in sorted CD164high/low fractions and CD90+/- fractions, and CD34+ cells at day 0 and day 4 in expansion culture. Shown are 3 independent BM. Left scheme represents the predicted path of differentiation from the most primitive fraction of CD34^{high}CD90^{pos} cells. The observed path of differentiation (right scheme) does not overlap.

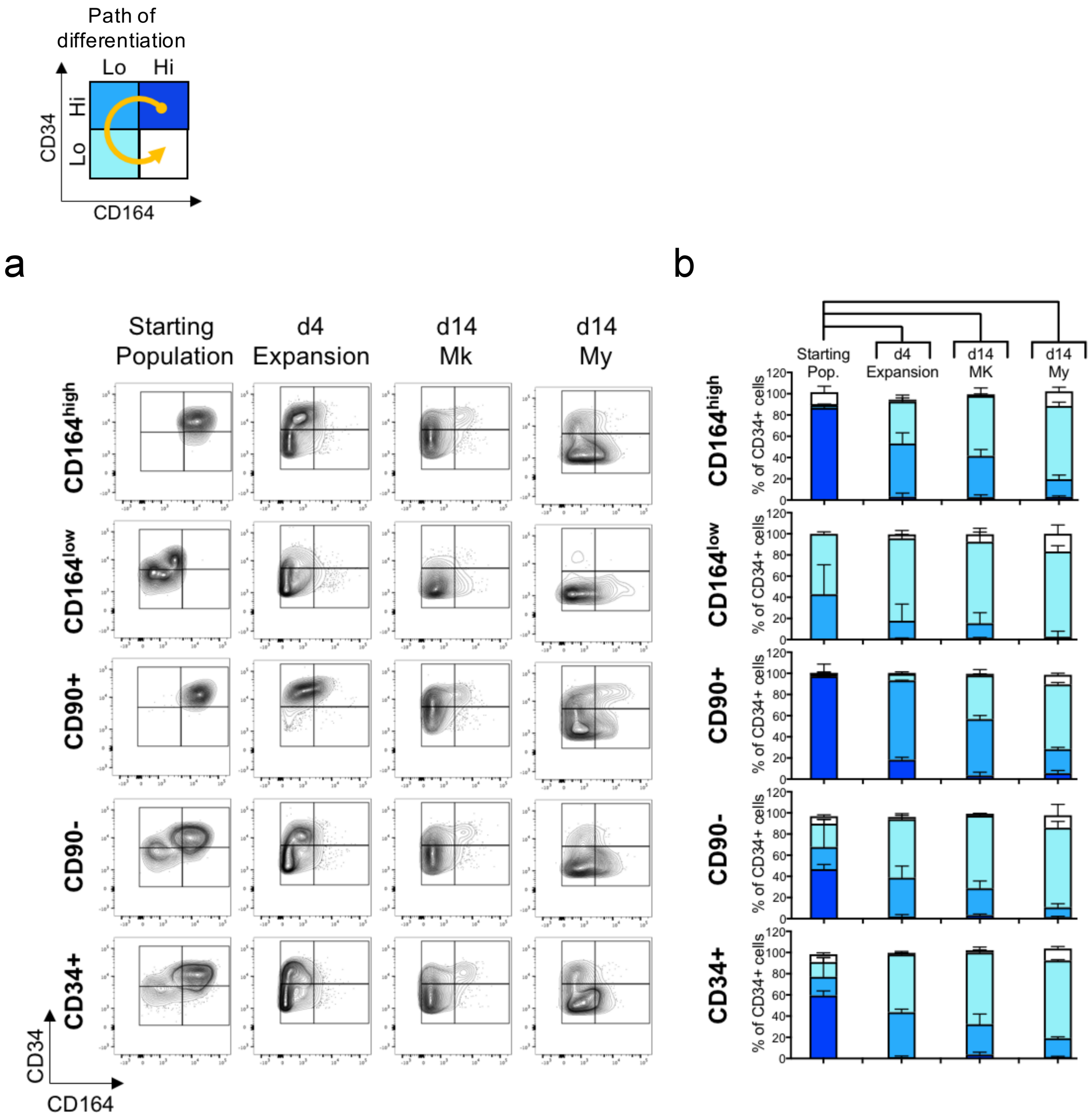

Supplementary Figure 21 | Path of differentiation of the sorted subsets through the analysis of CD164 and CD34 expression. a) FACS plots showing the cell phenotype at day 0 (Starting population) and in differentiation states at day 4 in Expansion culture, or at day 14 in Mk and My culture conditions. Top left, scheme representing the path of differentiation from the most primitive fraction of CD34^{high}CD164^{high} cells. **b)** Bar graphs summarizing the cytometric analysis described in a). The colour code used is the same of the scheme in a). Shown are Median ± Error from 3 independent BM.

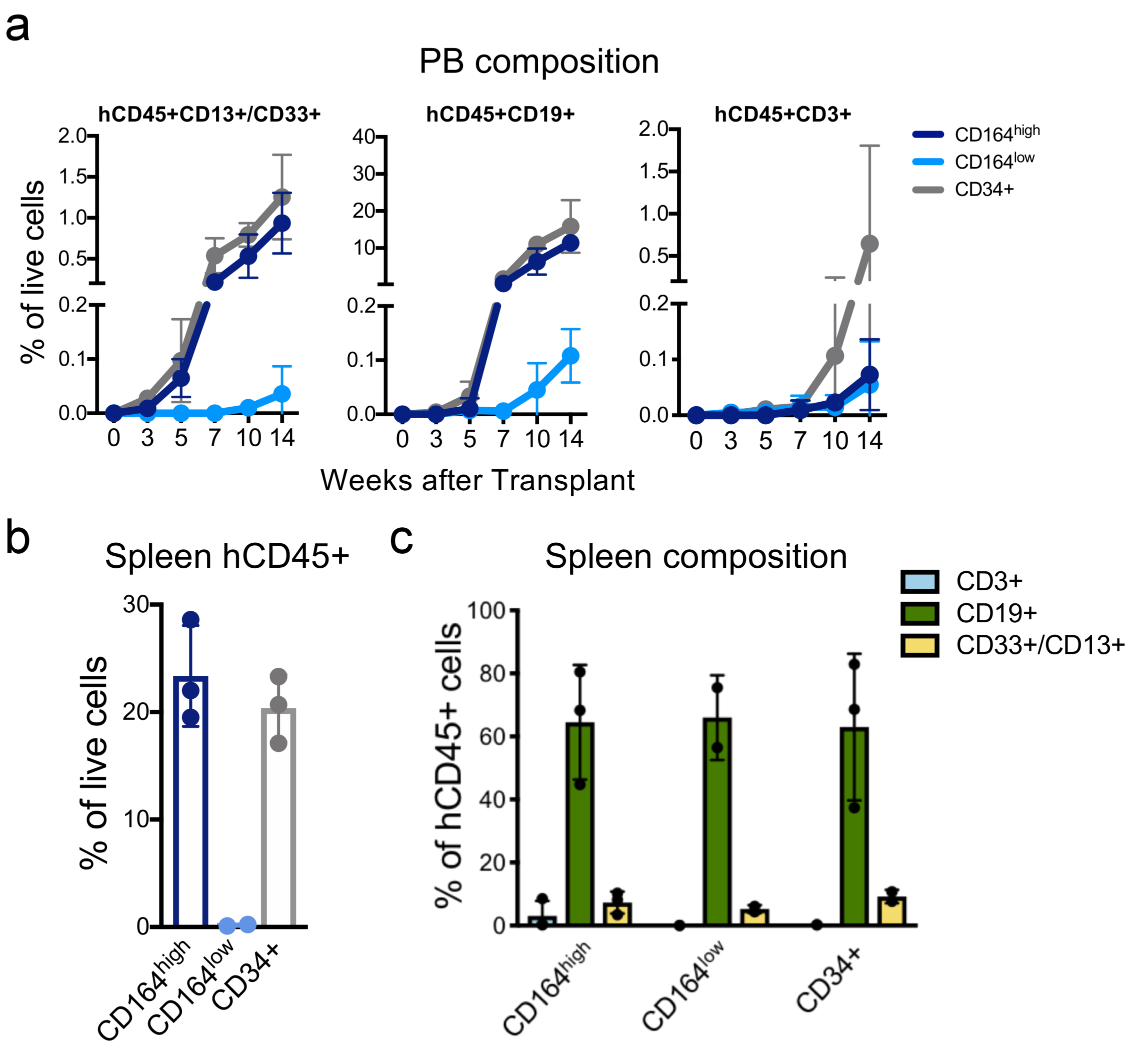

Supplementary Figure 22 | Human engraftment in PB and spleen of transplanted NBSGW mice. **a)** Composition of human CD45+ cells in murine PB at the indicated number of weeks post-transplant. Myeloid and lymphoid reconstitution were analysed within the human CD45+ population. **b)** Human CD45+ cell engraftment in murine spleen at 16 weeks post-transplant. Color legend as in a). **c)** Composition of human CD45+ cells showed in b). N= 3-4 mice per group.

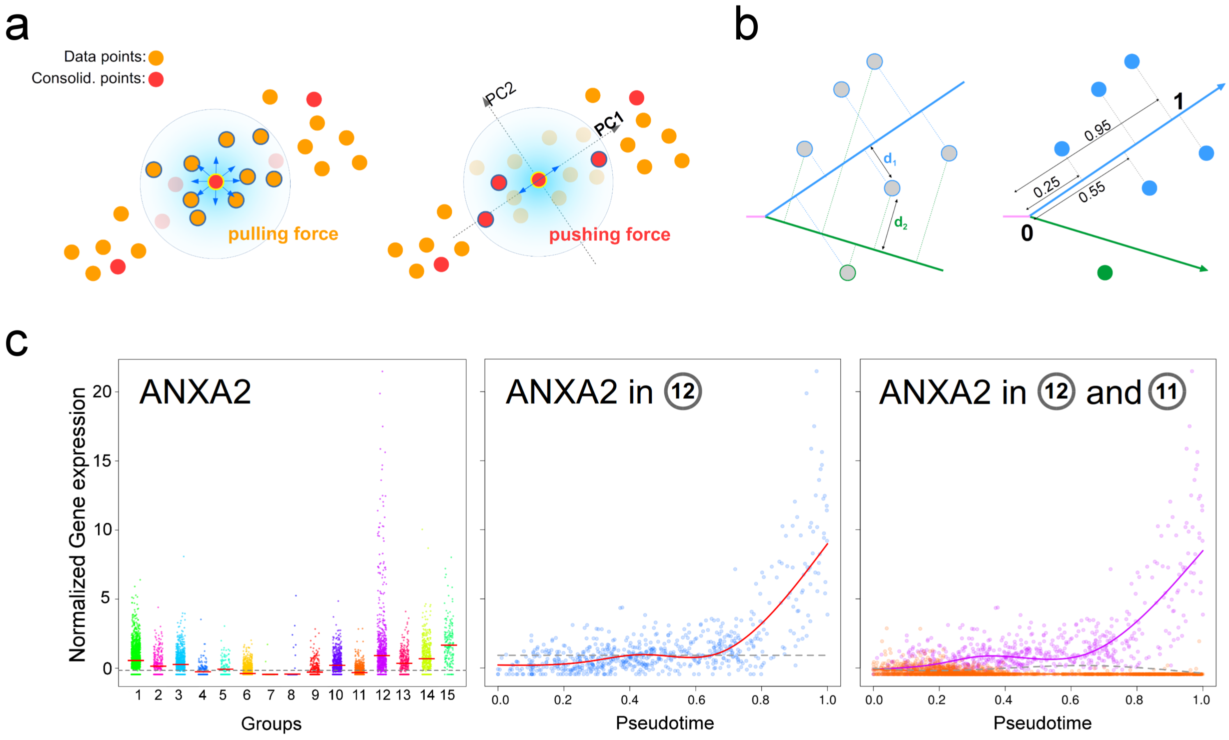

Supplementary Figure 23 | Explanatory figures for structure-aware filtering algorithm and gene expression analyses. a) Structure-aware filtering[5] is based on the iterative update of consolidation points positions according to a velocity field with two components. The pulling force (left) make consolidation points to move towards regions enriched for data points. The repulsion term (right) is the sum of all the pushing forces exerted by neighbour consolidation points and can only move points on a specific, locally optimal, line of action (first principal component). **b)** Each cell is associated to the closest branch by comparing cell-trajectories orthogonal distances (left). Cell pseudotime value, measuring the progression status along the differentiation process, is given by the rescaled distance between cell orthogonal projection onto the associated trajectory and branch stem point (right). **c)** Examples for gene expression analyses performed. Left, ANXA2 group-wise gene expression (adjusted p-value: 9.31E-159). Red lines correspond to groups averages (M_1) and the dashed grey line corresponds to the overall mean (M_0). Centre, ANXA2 association with progression (pseudotime) along branch 12 (Monocytes) in Lin-CD34/CD164 transcriptome map (adjusted p-value: 1.0821E-126). Red line is the spline based regression curve (M_1), whereas dashed grey line represents the restricted model M_0. Right, ANXA2 expression comparison among groups 11 (Neutrophils) and 12 (Monocytes). Group specific regression curves with common intercept (M_1) are shown with solid coloured lines. The dashed grey line corresponds to the nonlinear model fitted without considering group labels (adjusted p-value: 1.3409E-260).

Supplementary Tables

Ref to Figure 1

Population	Sorted events	Estimated barcoded cells	Post-filtering barcoded cells	Gate on CD34+ cells
**HSC**	25237	4000	1282	Lin-CD34+CD38-CD90+CD45RA-
**MPP**	4133	1000	215	Lin-CD34+CD38-CD90-CD45RA-
**MLP**	2186	N.A*	123	Lin-CD34+CD38-CD90-CD45RA+
**PREB/NK**	2541	N.A*	592	Lin-CD34+CD38+CD7-CD10+
**MEP**	21964	4000	1211	Lin-CD34+CD38+CD7-CD10-CD135-CD45RA-
**CMP**	31832	4000	1576	Lin-CD34+CD38+CD7-CD10-CD135+CD45RA-
**GMP**	16065	2000	1012	Lin-CD34+CD38+CD7-CD10-CD135+CD45RA+

Ref to Figure 2

Population	Sorted events	Estimated barcoded cells	Post-filtering barcoded cells	Gate on MNC
**Lin-CD34+CD164+**	426759	5300	6343	Lin-CD34+CD164+
**Lin-CD34lowCD164high**	252909	5000	4266	Lin-CD34lowCD164high
**Lin-CD34-CD164high**	663506	5000	4434	Lin-CD34-CD164high
**Lin-CD34-CD164low**	71220	300	358	Lin-CD34-CD164low

Supplementary Table 1 | Sorted events and estimated/ post-filtering barcoded cells in each population analysed. The information of numbers of sorted events and estimated/post-filtering barcoded cells from each population analysed are provided in this table, together with the surface marker profile of each population.

Population	Post-filtering barcoded cells	FACS gating proportion	Individual barcode weight
HSC	1282	0.132	1.03E-04
MPP	215	0.280	1.30E-03
MLP	123	0.044	3.55E-04
PreB/NK	592	0.019	3.26E-05
MEP	1211	0.006	4.96E-06
CMP	1576	0.325	2.07E-04
GMP	1012	0.193	1.91E-04

Population	Post-filtering barcoded cells	FACS gating proportion	Individual barcode weight
Lin-CD34+CD164+	6343	0.074	1.30E-05
Lin-CD34lowCD164high	4266	0.073	1.78E-05
Lin-CD34-CD164high	4434	0.728	1.78E-04
Lin-CD34-CD164low	358	0.048	1.45E-04

Supplementary Table 2 | Details for the generation of the observed and predicted cell density estimations shown in Supplementary Figure 1.

Subpopulation	Parameter [P(Subpop)]	CD135- Parameter Est. [MoM]	CD135- Parameter Est. S.E. [MoM]	CD135+ Parameter Est. [MoM]	CD135+ Parameter Est. S.E. [MoM]	T-test P-value	CD135-	CD135+
HSC	pi:HSC	2.420E-02	2.171E-02	3.077E-02	1.942E-02	7.16E-01	-	-
MPP	pi:MPP	5.827E-02	6.947E-02	3.728E-02	3.861E-02	6.77E-01	-	-
MLP	pi:MLP	1.091E-02	7.681E-03	9.381E-03	3.809E-03	7.78E-01	-	-
ETP	pi:ETP	3.408E-02	2.237E-03	2.629E-02	1.481E-02	4.59E-01	-	-
PREB/NK	pi:PREB/NK	3.289E-01	1.700E-01	4.136E-02	1.742E-02	9.80E-02	-	-
MEP	pi:MEP	4.480E-01	1.103E-01	NA	NA	NA	▲	▼
CD10-CD45+	pi:CD10-CD45+	7.306E-02	3.282E-02	NA	NA	NA	▲	▼
CMP	pi:CMP	1.989E-02	8.857E-03	4.570E-01	3.234E-02	**9.49E-04**	▼	▲
GMP	pi:GMP	2.694E-03	2.332E-03	3.979E-01	6.380E-02	**8.51E-03**	▼	▲

Supplementary Table 3 | Statistical analysis on the HSPC bar graph of Figure 3d. The method of moment estimation of each HSPC subpopulation proportion with related standard deviation are provided in these tables. The comparison of proportion estimates in the CD135- and CD135+ fractions have been calculated by means of the Student's t-test, under different variances hypothesis (BM #10,11,12).

Figure 5d

BM #1,2,3,4,5,6,7,8,9

Subpopulation	Parameter [P(Subpop)]	CD164high Parameter Est. [MoM]	CD164high Parameter Est. S.E. [MoM]	CD164low Parameter Est. [MoM]	CD164low Parameter Est. S.E. [MoM]	T-test P-value	CD164high	CD164low
HSC	pi:HSC	3.420E-02	2.270E-02	7.590E-04	1.283E-03	**2.21E-03**	▲	▼
MPP	pi:MPP	5.441E-02	4.562E-02	6.130E-03	7.878E-03	**1.31E-02**	▲	▼
MLP	pi:MLP	8.778E-03	4.213E-03	5.018E-03	5.737E-03	1.34E-01	-	-
ETP	pi:ETP	1.935E-02	7.883E-03	2.042E-02	7.231E-03	7.67E-01	-	-
PREB/NK	pi:PREB	1.999E-02	6.904E-03	1.094E-01	4.410E-02	**2.65E-04**	▼	▲
CMP/MEP	pi:CMP/MEP	4.849E-01	4.284E-02	1.081E-01	7.906E-02	**2.16E-08**	▲	▼
GMP	pi:GMP	2.969E-01	5.665E-02	3.205E-01	4.973E-02	3.63E-01	-	-
LINp	pi:LINp	8.148E-02	3.837E-02	4.297E-01	1.263E-01	**1.79E-05**	▼	▲

Subpopulation	Parameter [P(Subpop)]	CD164high Parameter Est. [MoM]	CD164high Parameter Est. S.E. [MoM]	CD34+ Parameter Est. [MoM]	CD34+ Parameter Est. S.E. [MoM]	T-test P-value	CD164high	CD34
HSC	pi:HSC	3.420E-02	2.270E-02	2.510E-02	1.906E-02	3.71E-01	-	-
MPP	pi:MPP	5.441E-02	4.562E-02	3.750E-02	2.945E-02	3.66E-01	-	-
MLP	pi:MLP	8.778E-03	4.213E-03	8.361E-03	4.455E-03	8.41E-01	-	-
ETP	pi:ETP	1.935E-02	7.883E-03	1.975E-02	7.595E-03	9.14E-01	-	-
PREB/NK	pi:PREB	1.999E-02	6.904E-03	4.238E-02	1.808E-02	**5.79E-03**	▼	▲
CMP/MEP	pi:CMP/MEP	4.849E-01	4.284E-02	3.818E-01	4.371E-02	**1.18E-04**	▲	▼
GMP	pi:GMP	2.969E-01	5.665E-02	3.086E-01	3.643E-02	6.12E-01	-	-
LINp	pi:LINp	8.148E-02	3.837E-02	1.765E-01	3.925E-02	**8.87E-05**	▼	▲

Subpopulation	Parameter [P(Subpop)]	CD164low Parameter Est. [MoM]	CD164low Parameter Est. S.E. [MoM]	CD34+ Parameter Est. [MoM]	CD34+ Parameter Est. S.E. [MoM]	T-test P-value	CD164low	CD34
HSC	pi:HSC	7.590E-04	1.283E-03	2.510E-02	1.906E-02	**4.98E-03**	▼	▲
MPP	pi:MPP	6.130E-03	7.878E-03	3.750E-02	2.945E-02	**1.28E-02**	▼	▲
MLP	pi:MLP	5.018E-03	5.737E-03	8.361E-03	4.455E-03	1.88E-01	-	-
ETP	pi:ETP	2.042E-02	7.231E-03	1.975E-02	7.595E-03	8.50E-01	-	-
PREB/NK	pi:PREB	1.094E-01	4.410E-02	4.238E-02	1.808E-02	**1.56E-03**	▲	▼
CMP/MEP	pi:CMP/MEP	1.081E-01	7.906E-02	3.818E-01	4.371E-02	**7.42E-07**	▼	▲
GMP	pi:GMP	3.205E-01	4.973E-02	3.086E-01	3.643E-02	5.71E-01	-	-
LINp	pi:LINp	4.297E-01	1.263E-01	1.765E-01	3.925E-02	**2.24E-04**	▲	▼

Supplementary Figure 13c

MPB #1,2,3,4

Subpopulation	Parameter [P(Subpop)]	CD164high Parameter Est. [MoM]	CD164high Parameter Est. S.E. [MoM]	CD164low Parameter Est. [MoM]	CD164low Parameter Est. S.E. [MoM]	T-test P-value	CD164high	CD164low
HSC	pi:HSC	5.166E-02	2.289E-02	1.233E-02	2.596E-03	9.50E-02	-	-
MPP	pi:MPP	9.830E-02	2.687E-02	2.739E-02	8.725E-03	**3.45E-02**	▲	▼
MLP	pi:MLP	2.815E-02	2.133E-02	1.324E-02	7.789E-03	3.52E-01	-	-
ETP	pi:ETP	6.949E-03	9.757E-03	1.073E-02	8.859E-03	6.46E-01	-	-
PREB/NK	pi:PREB	1.400E-02	1.266E-02	2.975E-02	1.803E-02	2.91E-01	-	-
CMP/MEP	pi:CMP/MEP	7.487E-02	5.858E-02	1.235E-01	7.267E-02	4.20E-01	-	-
GMP	pi:GMP	7.209E-01	5.484E-02	7.759E-01	8.693E-02	4.15E-01	-	-
LINp	pi:LINp	5.199E-03	3.574E-03	7.126E-03	5.662E-03	6.49E-01	-	-

		CD164^{high}		CD34+				
**Subpopulation**	**Parameter [P(Subpop)]**	**Parameter Est.** [MoM]	**Parameter Est. S.E. [MoM]**	**Parameter Est.** [MoM]	**Parameter Est. S.E. [MoM]**	**T-test P-value**	**CD164^{high}**	**CD34**
HSC	pi:HSC	5.166E-02	2.289E-02	3.219E-02	1.480E-02	2.94E-01	-	-
MPP	pi:MPP	9.830E-02	2.687E-02	6.261E-02	1.798E-02	1.39E-01	-	-
MLP	pi:MLP	2.815E-02	2.133E-02	1.897E-02	1.081E-02	5.54E-01	-	-
ETP	pi:ETP	6.949E-03	9.757E-03	7.226E-03	9.422E-03	9.73E-01	-	-
PREB/NK	pi:PREB	1.400E-02	1.266E-02	1.961E-02	1.139E-02	5.99E-01	-	-
CMP/MEP	pi:CMP/MEP	7.487E-02	5.858E-02	1.039E-01	7.494E-02	6.27E-01	-	-
GMP	pi:GMP	7.209E-01	5.484E-02	7.495E-01	6.642E-02	5.97E-01	-	-
LINp	pi:LINp	5.199E-03	3.574E-03	5.999E-03	3.254E-03	7.89E-01	-	-

		CD164^{low}		CD34+				
**Subpopulation**	**Parameter [P(Subpop)]**	**Parameter Est.** [MoM]	**Parameter Est. S.E. [MoM]**	**Parameter Est.** [MoM]	**Parameter Est. S.E. [MoM]**	**T-test P-value**	**CD164^{low}**	**CD34**
HSC	pi:HSC	1.233E-02	2.596E-03	3.219E-02	1.480E-02	1.42E-01	-	-
MPP	pi:MPP	2.739E-02	8.725E-03	6.261E-02	1.798E-02	5.80E-02	-	-
MLP	pi:MLP	1.324E-02	7.789E-03	1.897E-02	1.081E-02	5.01E-01	-	-
ETP	pi:ETP	1.073E-02	8.859E-03	7.226E-03	9.422E-03	6.64E-01	-	-
PREB/NK	pi:PREB	2.975E-02	1.803E-02	1.961E-02	1.139E-02	4.65E-01	-	-
CMP/MEP	pi:CMP/MEP	1.235E-01	7.267E-02	1.039E-01	7.494E-02	7.61E-01	-	-
GMP	pi:GMP	7.759E-01	8.693E-02	7.495E-01	6.642E-02	6.99E-01	-	-
LINp	pi:LINp	7.126E-03	5.662E-03	5.999E-03	3.254E-03	7.83E-01	-	-

Supplementary Table 4 | Statistical analysis on the HSPC bar graphs of Figure 5d and Supplementary Figure 13c. The method of moment estimation of each HSPC subpopulation proportion with related standard deviation are provided in these tables. The comparison of proportion estimates in the CD164high and CD164low fractions, and CD34+ cells have been calculated by means of the Student's t-test, under different variance hypothesis.

Supplementary References

1. Haghverdi, L., Buettner, F. & Theis, F. J. Diffusion maps for high-dimensional single-cell analysis of differentiation data. *Bioinformatics* **31,** 2989–2998 (2015).
2. Uhlen, Mathias, et al. The human protein atlas. *Retrived from: http://www. proteinatlas. Org* (2015).
3. Forbes, Simon A., et al. COSMIC: somatic cancer genetics at high-resolution. Nucleic acids research 45.D1 (2016): D777-D783.
4. Croft, David, et al. The Reactome pathway knowledgebase. Nucleic acids research 42.D1 (2013): D472-D477.
5. Wu, Shihao, et al. Structure-aware Data Consolidation. *IEEE transactions on pattern analysis and machine intelligence* (2017).
