# Supplementary material for: A comprehensive single cell transcriptional landscape of human hematopoietic progenitors
Source: Nat Commun. 2019 Jun 3;10:2395. doi: 10.1038/s41467-019-10291-0 (PMC6546699; doi:10.1038/s41467-019-10291-0)
Supplement: Supplementary file 2 — Reporting Summary [file 41467_2019_10291_MOESM2_ESM.pdf]

nature research

Corresponding author(s): *Double-blind peer review submissions: write DBPR and your manuscript number here instead of author names.*

Last updated by author(s): *YYYY-MM-DD*

Reporting Summary

Nature Research wishes to improve the reproducibility of the work that we publish. This form provides structure for consistency and transparency in reporting. For further information on Nature Research policies, see Authors & Referees and the Editorial Policy Checklist.

Statistics

For all statistical analyses, confirm that the following items are present in the figure legend, table legend, main text, or Methods section.

n/a	Confirmed	
☐	☒	The exact sample size (n) for each experimental group/condition, given as a discrete number and unit of measurement
☐	☒	A statement on whether measurements were taken from distinct samples or whether the same sample was measured repeatedly
☐	☒	The statistical test(s) used AND whether they are one- or two-sided<br>*Only common tests should be described solely by name; describe more complex techniques in the Methods section.*
☐	☒	A description of all covariates tested
☐	☒	A description of any assumptions or corrections, such as tests of normality and adjustment for multiple comparisons
☐	☒	A full description of the statistical parameters including central tendency (e.g. means) or other basic estimates (e.g. regression coefficient) AND variation (e.g. standard deviation) or associated estimates of uncertainty (e.g. confidence intervals)
☐	☒	For null hypothesis testing, the test statistic (e.g. F, t, r) with confidence intervals, effect sizes, degrees of freedom and P value noted<br>*Give P values as exact values whenever suitable.*
☒	☐	For Bayesian analysis, information on the choice of priors and Markov chain Monte Carlo settings
☐	☒	For hierarchical and complex designs, identification of the appropriate level for tests and full reporting of outcomes
☐	☒	Estimates of effect sizes (e.g. Cohen's d, Pearson's r), indicating how they were calculated

Our web collection on statistics for biologists contains articles on many of the points above.

Software and code

Policy information about availability of computer code

Data collection	Illumina NextSeq 500 output were analyzed using indrops suite (https://github.com/indrops/indrops).
Data analysis	Knn graphs were generated using SPRING (https://github.com/AllonKleinLab/SPRING). PBA analysis were performed using PBA scripts (https://github.com/AllonKleinLab/PBA). Structure aware data consolidation algorithm described in Wu, Shihao et al (2017) has been implemented in R software (https://github.com/BiascoLab/PrincipalDevelopementalTrajectories)

For manuscripts utilizing custom algorithms or software that are central to the research but not yet described in published literature, software must be made available to editors/reviewers. We strongly encourage code deposition in a community repository (e.g. GitHub). See the Nature Research guidelines for submitting code & software for further information.

Data

Policy information about availability of data

All manuscripts must include a data availability statement. This statement should provide the following information, where applicable:

- Accession codes, unique identifiers, or web links for publicly available datasets
- A list of figures that have associated raw data
- A description of any restrictions on data availability

Data Availability
Raw data are available with GEO accession code GSE117498 [https://www.ncbi.nlm.nih.gov/geo/query/acc.cgi?acc=GSE117498]. SPRING plots are available for inspection at the following links: Mouse Kit+, [https://kleintools.hms.harvard.edu/tools/springViewer_1_6_dev.html?datasets/mouse_HPCs/basal_bone_marrow/full] ; Human LIN- CD164/CD34, [https://kleintools.hms.harvard.edu/tools/springViewer_1_6_dev.html?datasets/CD34_CD164/CD34_CD164]; Human sorted HSPC, [https://kleintools.hms.harvard.edu/tools/springViewer_1_6_dev.html?datasets/sortedHSPC/sortedHSPC]. The source data underlying Figures 1g, 2e, 3, 4b,c, 5, Supplementary Figures 7, 10, 12, 13, 15-17, 21-23 are provided as a Source Data File

Field-specific reporting

Please select the one below that is the best fit for your research. If you are not sure, read the appropriate sections before making your selection.

☒ Life sciences ☐ Behavioural & social sciences ☐ Ecological, evolutionary & environmental sciences

For a reference copy of the document with all sections, see nature.com/documents/nr-reporting-summary-flat.pdf

Life sciences study design

All studies must disclose on these points even when the disclosure is negative.

Sample size	For sufficient statistical power, a minimum sample size of three healthy donor bone marrow CD34+ cells was chosen to perform the immunophenotyping analyses and the in vitro functional assays. Where indicated, a larger sample size was used.
Data exclusions	No data were excluded from the immunophenotyping analyses and the in vitro/in vivo functional assays.
Replication	Experimental findings from the immunophenotyping analyses and the in vitro functional assays were reproduced.
Randomization	The human bone marrow samples used in this work were randomly allocated into the experimental groups. Mice were randomized in the different transplantation groups.
Blinding	No blinding was performed during data collection and analysis.

Reporting for specific materials, systems and methods

We require information from authors about some types of materials, experimental systems and methods used in many studies. Here, indicate whether each material, system or method listed is relevant to your study. If you are not sure if a list item applies to your research, read the appropriate section before selecting a response.

Materials & experimental systems

n/a	Involved in the study	
☐	☒	Antibodies
☒	☐	Eukaryotic cell lines
☒	☐	Palaeontology
☐	☒	Animals and other organisms
☒	☐	Human research participants
☒	☐	Clinical data

Methods

n/a	Involved in the study	
☒	☐	ChIP-seq
☐	☒	Flow cytometry
☒	☐	MRI-based neuroimaging

Antibodies

Antibodies used	All antibodies were purchased from Biolegend, BD Biosciences and Miltenyi Biotec. Antibodies used are described in Methods.
Validation	All antibody used were validated by providers.

Animals and other organisms

Policy information about studies involving animals; ARRIVE guidelines recommended for reporting animal research

Laboratory animals	Five week-old NBSGW female mice were purchased from the Jackson Laboratory.
Wild animals	This study did not involve wild animals.
Field-collected samples	This study did not involve samples collected by the field.
Ethics oversight	All animal procedures were performed according to protocols approved by IACUC.

Note that full information on the approval of the study protocol must also be provided in the manuscript.

Flow Cytometry

Plots

Confirm that:

☒ The axis labels state the marker and fluorochrome used (e.g. CD4-FITC).

☒ The axis scales are clearly visible. Include numbers along axes only for bottom left plot of group (a 'group' is an analysis of identical markers).

☒ All plots are contour plots with outliers or pseudocolor plots.

☒ A numerical value for number of cells or percentage (with statistics) is provided.

Methodology

Sample preparation	Cells were prepared from human bone marrow as described in Methods section, or were purchased from commercial sources (AllCells).
Instrument	FACSAria II sorter and LSR Fortessa analyser (all BD Biosciences).
Software	Flow cytometry data were collected with BD FACSDIVA software and analysed with FlowJo (Tree Star).
Cell population abundance	When analysed at the FACSAria II, post sort cell fractions were at least 98% pure.
Gating strategy	This information is provided in Fig. 1 and Fig. 2, and included in the Methods section.

☒ Tick this box to confirm that a figure exemplifying the gating strategy is provided in the Supplementary Information.
